# 1-(Piperidin-3-yl)thymine amides as inhibitors of *M. tuberculosis* thymidylate kinase

**DOI:** 10.1080/14756366.2019.1662790

**Published:** 2019-12-11

**Authors:** Yanlin Jian, Martijn D. P. Risseeuw, Mathy Froeyen, Lijun Song, Davie Cappoen, Paul Cos, Hélène Munier-Lehmann, Serge van Calenbergh

**Affiliations:** a Laboratory for Medicinal Chemistry, Faculty of Pharmaceutical Sciences, Ghent University, Ghent, Belgium;; b Department of Medicinal Chemistry, Rega Institute for Medical Research, KU Leuven, Leuven, Belgium;; c Laboratory for Microbiology, Parasitology and Hygiene (LMPH), Department of Pharmaceutical Sciences, University of Antwerp, Antwerp, Belgium;; d Unit of Chemistry and Biocatalysis, Department of Structural Biology and Chemistry, Institut Pasteur, CNRS UMR3523, Paris, France

**Keywords:** Thymidylate kinase, inhibitors, *Mycobacterium tuberculosis*, modelling

## Abstract

A series of readily accessible 1-(piperidin-3-yl)thymine amides was designed, synthesised and evaluated as *Mycobacterium tuberculosis* TMPK (*Mtb*TMPK) inhibitors. In line with the modelling results, most inhibitors showed reasonable *Mtb*TMPK inhibitory activity. Compounds **4b** and **4i** were slightly more potent than the parent compound **3**. Moreover, contrary to the latter, amide analogue **4g** was active against the avirulent *M. tuberculosis* H37Ra strain (MIC_50_=35 µM). This finding opens avenues for future modifications.

## 1. Introduction

Tuberculosis (TB) is a severe air transmitted infectious disease mainly caused by *Mycobacterium tuberculosis* (*Mtb*) and causes significant morbidity and mortality worldwide^1^. It is estimated that 10.4 million people fell ill with TB in 2017, and 1.6 million people succumbed the infection^2^. Standard treatment of drug-sensitive TB normally lasts 6 months, including a 2-month course with at least three first-line drugs and a 4-month course with at least Isoniazid (INH) and Rifampicin (RIF), resulting in poor treatment compliance and a high financial burden. Moreover, the increasing number of multidrug-resistant TB (MDR-TB), extensively drug-resistant TB (XDR-TB) and rifampicin-resistant TB (RR-TB) makes the treatment even more challenging^3^^,^^4^. New drugs acting on yet unexplored targets/pathways are desirable to achieve worldwide TB control.

**Figure 1.**
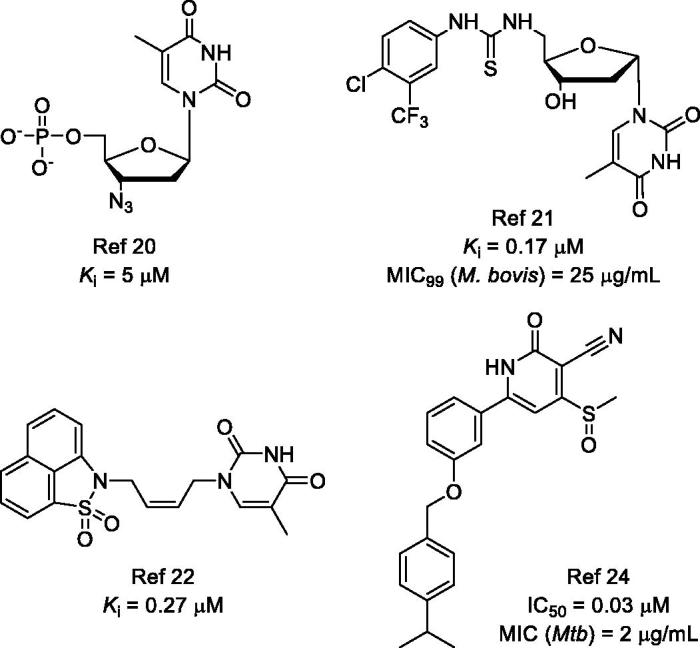
Representative thymidine-like and non-nucleoside *Mtb*TMPK inhibitors.

Thymidine monophosphate kinase (TMPK), an enzyme at the junction of the *de novo* (involving thymidylate synthase) and salvage pathway, is responsible for the conversion of thymidine monophosphate (TMP) to thymidine diphosphate (TDP)^5^^,^^6^. Further phosphorylation leads to the formation of thymidine triphosphate (TTP), which is indispensable for DNA synthesis^7^. *Mycobacterium tuberculosis* TMPK (*Mtb*TMPK) shows low (22%) sequence identity with the human isozyme and has a unique catalytic mechanism^8^^,^^9^, further supporting it as an attractive target for developing new anti-TB drugs. Both industrial and academic efforts have afforded several potent *Mtb*TMPK inhibitors in the past two decennia^10–19^, including thymidine-like and non-nucleoside inhibitors (Figure 1)^20–24^.

**Figure 2.**
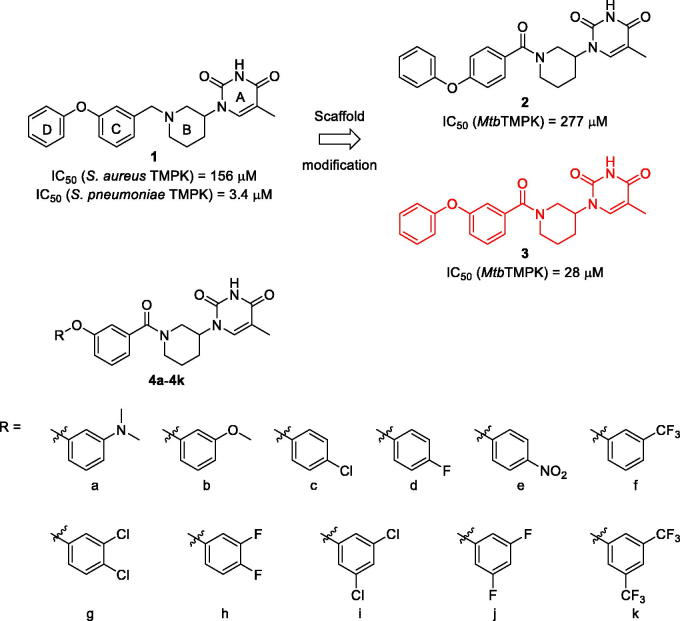
Structures of regiomeric racemates **2** and **3** derived from Gram-positive bacterial thymidylate kinase inhibitor **1** and amide analogues **4a**–**4k** investigated in this study.

Using the Gram-positive bacterial thymidylate kinase inhibitor **1** (Figure 2) disclosed by Astra Zeneca^7^ as a starting point, our research group is trying to identify potent non-nucleoside *Mtb*TMPK inhibitors^25^. These efforts resulted in the identification of the regiomeric racemates **2** and **3**, of which the latter showed the best *Mtb*TMPK inhibitory activity and was therefore selected for further optimisation. In this study, we synthesised a series of D-ring substituted analogues of **3** and evaluated these as *Mtb*TMPK inhibitors (Figure 2).

## 2. Materials and methods

### 2.1. Chemistry

All synthetic compounds mentioned in this study were visualised under UV light at 254 nm or stained by corresponding reagents, and purified by column chromatography (CC) on a Reveleris X2 (Grace) automated flash unit. ^1^H and ^13^C NMR spectral data were obtained from a Varian Mercury 300/75 MHz spectrometer at 300 K using TMS as an internal standard. Structural assignment was confirmed with the assistance of COSY, HSQC and HMBC. High-resolution mass spectrometry was performed on a Waters LCT Premier XETM time of flight (TOF) mass spectrometer equipped with a standard electrospray ionisation (ESI) and modular LockSpray TM interface. The purity of the tested compounds was determined by LC-MS analysis (Waters Alliance 2695 XE separation module).

*3-((benzyloxy)methyl)-1–(1-(3-iodobenzoyl)piperidin-3-yl)-5-methylpyrimidine-2,4(1H,3H)-dione* (**6**) Intermediate **8** was synthesised as previously reported^25^. To a solution of **8** (200.00 mg, 0.61 mmol) in dichloromethane (12.00 ml) were added N,N-bis(isopropyl) carbondiimide (153.72 mg, 1.22 mmol), 3-iodobenzoic acid (225.88 mg, 0.91 mmol) and 4-dimethylaminopyridine (7.45 mg, 0.06 mmol), the reaction mixture was stirred at room temperature for overnight. After complete consumption of **8** checked with TLC, the reaction mixture was diluted with dichloromethane (60.00 ml), and washed with 1 M HCl (60.00 ml), saturated NaHCO_3_ (60.00 ml) and brine (60.00 ml). The organic layer was collected and dried over anhydrous Na_2_SO_4_, followed by filtered and concentrated *in vacuo*. The resulting residue was purified by column chromatography (petroleum ether—EtOAc, 4: 1 v/v) to give solid **6** (230.96 mg, 68.00% yield), which was characterised by ^1^H and ^13 ^C-NMR, and ESI-HRMS. ^1^H NMR: *δ_H_* (300 MHz, CDCl_3_, TMS) 1.53–2.02 (m, 7 H, piperidin-4-yl, piperidin-5-yl, CH_3_), 2.83–3.25 (m, 2 H, piperidin-2a-yl, piperidin-6a-yl), 3.66–4.04 (m, 2 H, piperidin-2b-yl, piperidin-6b-yl), 4.34–4.50 (m, 1 H, piperidin-3-yl), 4.70 (s, 2 H, O-CH_2_-phenyl), 5.50 (s, 2 H, N-CH_2_-O), 6.93 (s, 1 H), 7.12–7.20 (m, 1 H), 7.23–7.45 (m, 6 H), 7.71–7.82 (m, 2 H). ^13^C NMR: *δ_C_* (75 MHz, CDCl_3_, TMS) 13.22 (5-CH_3_), 24.84 (piperidin-5-yl), 29.07 (piperidin-4-yl), 53.74 (piperidin-3-yl), 60.38 (piperidin-2-yl), 70.94 (N-CH_2_-O), 72.33 (O-CH_2_-phenyl), 94.30 (I-C), 110.41 (C-5), 126.11 (Ph), 127.51 (2 C, Ph), 127.57 (Ph), 128.22 (2 C, Ph), 130.29 (Ph), 135.83 (Ph), 137.20 (Ph), 138.08 (Ph), 139.02 (2 C, C-5, Ph), 151.22 (C-2), 163.02 (C-4), 168.88 (CO, benzoyl). C (piperidin-6-yl) cannot be found. HRMS (ESI): m/z [M + H]^+^ Calcd. for [C_25_H_26_IN_3_O_4_+H] ^+^ 560.1041, found 560.1047.

*3-((benzyloxy)methyl)-1–(1-(3-hydroxybenzoyl)piperidin-3-yl)-5-methylpyrimidine-2,4(1H,3H)-dione* (**10**) To a solution of **8** (1.19 g, 3.62 mmol) in dichloromethane (72 ml) were added N,N-bis(isopropyl) carbondiimide (913.60 mg, 7.24 mmol), 3-hydroxybenzoic acid (749.99 mg, 5.43 mmol) and 4-dimethylaminopyridine (43.98 mg, 0.36 mmol), the reaction mixture was stirred at room temperature for overnight. After complete consumption of **8** checked with TLC, the reaction mixture was diluted with dichloromethane (200 ml), and washed with 1 M HCl (100 ml), saturated NaHCO_3_ (100 ml) and brine (100 ml). The organic layer was collected and dried over anhydrous Na_2_SO_4_, followed by filtered and concentrated *in vacuo*. The resulting residue was purified by column chromatography (petroleum ether—EtOAc, 3: 1 v/v) to give colourless liquid **10** (1.01 g, 62.12% yield), which was characterised by ^1^H and ^13 ^C-NMR, and ESI-HRMS. ^1^H NMR: *δ_H_* (300 MHz, CDCl_3_, TMS) 1.59–2.10 (m, 7 H, piperidin-4-yl, piperidin-5-yl, CH_3_), 2.64–2.83 (m, 1 H, piperidin-6a-yl) 2.88–3.12 (m, 1 H, piperidin-2a-yl), 3.59–4.15 (m, 2 H, piperidin-2b-yl, piperidin-6b-yl), 4.32–4.53 (m, 1 H, piperidin-3-yl), 4.69 (s, 2 H, O-CH_2_-phenyl), 5.50 (s, 2 H, N-CH_2_-O), 6.74–7.08 (m, 4 H), 7.12–7.44 (m, 6 H), 7.68 (br. s., 1 H, OH). ^13 ^C NMR: *δ_C_* (75 MHz, CDCl_3_, TMS) 13.18 (5-CH_3_), 24.66 (piperidin-5-yl), 29.04 (piperidin-4-yl), 47.63 (piperidin-6-yl), 53.64 (piperidin-3-yl), 70.99 (N-CH_2_-O), 72.33 (O-CH_2_-phenyl), 110.54 (C-5), 114.32 (Ph), 117.76 (Ph), 118.47 (Ph), 127.50 (2 C, Ph), 127.59 (Ph), 128.21 (2 C, Ph), 129.97 (Ph), 135.82 (Ph), 137.97 (2 C, C-6, Ph), 151.36 (C-2), 156.70 (Ph), 163.03 (C-4), 170.91 (CO, benzoyl). C (piperidin-2-yl) cannot be found. HRMS (ESI): m/z [M + H]^+^ Calcd. for [C_25_H_27_N_3_O_5_+H]^+^ 450.2024, found 450.2036.

*General procedure for substituted 3-((benzyloxy)methyl)-5-methyl-1–(1-(3 phenoxybenzoyl)piperidin-3-yl)pyrimidine-2,4(1H,3H)-dione* (**13b–13k**). Substituted iodobenzene (0.66 mmol) was added to a stirred solution of **10** (0.44 mmol), copper iodide (0.18 mmol), potassium phosphate tribasic (0.89 mmol), picolinic acid (0.27 mmol) in DMSO under nitrogen and heated at 90 °C for overnight. The reaction mixture was cooled to room temperature, quenched with water (50 ml) and extracted with CH_2_Cl_2_ (100 ml). The separated organic layer was further washed with 1 M HCl (50 ml), saturated NaHCO_3_ (50 ml) and brine (50 ml), dried over anhydrous Na_2_SO_4_, and evaporated *in vacuo*. The concentrated crude was purified by column chromatography (petroleum ether—EtOAc, 1:1 v/v) to give colourless liquid **13a**–**13k** (40.00–70.00% yield), which was characterised by ^1^H and ^13^C-NMR, and ESI-HRMS.

**13a** following the general procedure, starting with 1-iodo-3-dimethylaminobenzene (163.07 mg), **10** (197.78 mg), copper iodide (33.80 mg), potassium phosphate tribasic (189.00 mg), picolinic acid (32.90 mg) in DMSO, **13a** was obtained (242.72 mg, 71.00% yield). ^1^H NMR: *δ_H_* (300 MHz, CDCl_3_, TMS) 1.72–2.12 (m, 7 H, piperidin-4-yl, piperidin-5-yl, CH_3_), 2.68–2.97 (m, 7 H, piperidin-6a-yl, N(CH_3_)_2_), 2.97–3.23 (m, 1 H, piperidin-2a-yl,), 3.68–4.03 (m, 2 H, piperidin-2b-yl, piperidin-6b-yl), 4.32–4.51 (m, 1 H, piperidin-3-yl), 4.70 (s, 2 H, O-CH_2_-phenyl), 5.50 (s, 2 H, N-CH_2_-O) , 6.33–6.42 (m, 1 H), 6.47 (s, 1 H), 6.53–6.61 (m, 1 H), 6.94 (br. s., 1 H), 7.05 (br. s., 1 H), 7.07–7.15 (m, 2 H), 7.16–7.30 (m, 4 H), 7.31–7.45 (m, 3 H). ^13 ^C NMR: *δ_C_* (75 MHz, CDCl_3_, TMS) 13.60 (5-CH_3_), 23.86 (piperidin-5-yl), 29.54 (piperidin-4-yl), 41.16 (2 C, N(CH_3_)_2_), 54.01 (piperidin-3-yl), 71.31 (N-CH_2_-O), 72.71 (O-CH_2_-phenyl), 104.69 (Ph), 108.28 (Ph), 109.06 (Ph), 110.70 (C-5), 117.01 (Ph), 120.09 (Ph), 121.42 (Ph), 127.92 (2 C, Ph), 127.97 (Ph), 128.63 (2 C, Ph), 130.41 (Ph), 130.58 (Ph), 136.23 (Ph), 137.16 (Ph), 138.47 (C-6), 151.60 (C-2), 157.63 (Ph), 158.41 (Ph), 163.48 (C-4), 170.48 (CO, benzoyl). C (piperidin-2-yl), C (piperidin-6-yl) and C (CN(CH_3_)_2_) cannot be found. HRMS (ESI): m/z [M + H]^+^ Calcd. for [C_33_H_36_N_4_O_5_+H]^+^ 569.2758, found 569.2773.

**13b** following the general procedure, starting with 1-iodo-3-methoxybenzene (154.46 mg), **10** (197.78 mg), copper iodide (33.80 mg), potassium phosphate tribasic (189.00 mg), picolinic acid (32.90 mg) in DMSO, **13b** was obtained (230.47 mg, 69.00% yield). ^1^H NMR: *δ_H_* (300 MHz, CDCl_3_, TMS) 1.54–1.73 (m, 1 H, piperidin-5a-yl), 1.79–2.18 (m, 6 H, piperidin-4-yl, piperidin-5b-yl, CH_3_), 2.75–3.20 (m, 2 H, piperidin-2a-yl, piperidin-6a-yl), 3.64–4.11 (m, 5 H, piperidin-2b-yl, piperidin-6b-yl, OCH_3_), 4.35–4.54 (m, 1 H, piperidin-3-yl), 4.70 (s, 2 H, O-CH_2_-phenyl), 5.50 (s, 2 H, N-CH_2_-O), 6.57–6.65 (m, 2 H), 6.65–6.75 (m, 1 H), 6.94 (br. s., 1 H), 7.00–7.13 (m, 2 H), 7.16 (d, *J* = 6.44 Hz, 1 H), 7.20–7.47 (m, 7 H). ^13 ^C NMR: *δ_C_* (75 MHz, CDCl_3_, TMS) 13.64 (5-CH_3_), 25.27 (piperidin-5-yl), 29.56 (piperidin-4-yl), 54.04 (piperidin-3-yl), 55.80 (OCH_3_), 71.31 (N-CH_2_-O), 72.71 (O-CH_2_-phenyl), 105.88 (Ph), 109.96 (Ph), 110.74 (C-5), 111.87 (Ph), 117.48 (Ph), 120.47 (Ph), 121.91 (Ph), 127.92 (2 C, Ph), 127.97 (Ph), 128.63 (2 C, Ph), 130.52 (Ph), 130.70 (Ph), 137.28 (2 C, Ph), 138.47 (C-6), 151.60 (C-2), 157.93 (2 C, Ph), 161.43 (Ph), 163.46 (C-4), 170.36 (CO, benzoyl). C (piperidin-2-yl) and C (piperidin-6-yl) cannot be found. HRMS (ESI): m/z [M + H]^+^ Calcd. for [C_32_H_33_N_3_O_6_+H]^+^ 556.2442, found 556.2465.

**13c** following the general procedure, starting with 1-iodo-4-chlorobenzene (157.38 mg), **10** (197.78 mg), copper iodide (33.80 mg), potassium phosphate tribasic (189.00 mg), picolinic acid (32.90 mg) in DMSO, **13c** was obtained (198.63 mg, 59.00% yield). ^1^H NMR: *δ_H_* (300 MHz, CDCl_3_, TMS) 1.57–1.77 (m, 1 H, piperidin-5a-yl), 1.84–2.12 (m, 6 H, piperidin-4-yl, piperidin-5b-yl, CH_3_), 2.71–3.19 (m, 2 H, piperidin-2a-yl, piperidin-6a-yl), 3.64–4.14 (m, 2 H, piperidin-2b-yl, piperidin-6b-yl), 4.35–4.54 (m, 1 H, piperidin-3-yl), 4.70 (s, 2 H, O-CH_2_-phenyl), 5.49 (s, 2 H, N-CH_2_-O), 6.84–7.01 (m, 3 H), 7.04 (d, *J* = 6.15 Hz, 2 H), 7.11–7.20 (m, 1 H), 7.21–7.60 (m, 8 H). ^13 ^C NMR: *δ_C_* (75 MHz, CDCl_3_, TMS) 13.15 (5-CH_3_), 24.78 (piperidin-5-yl), 29.02 (piperidin-4-yl), 70.85 (N-CH_2_-O), 72.25 (O-CH_2_-phenyl), 110.28 (C-5), 116.92 (Ph), 119.91 (Ph), 120.60 (2 C, Ph), 121.63 (Ph), 127.45 (2 C, Ph), 127.53 (Ph), 128.17 (3 C, Ph), 129.86 (2 C, Ph), 130.17 (Ph), 136.96 (2 C, Ph), 138.00 (C-6), 151.14 (C-2), 154.96 (C, Ph), 157.35 (Ph), 163.00 (C-4), 169.80 (CO, benzoyl). C (piperidin-2-yl), C (piperidin-3-yl) and C (piperidin-6-yl) cannot be found. HRMS (ESI): m/z [M + H]^+^ Calcd. for [C_31_H_30_ClN_3_O_5_+H]^+^ 560.1947, found 560.1954.

**13d** following the general procedure, starting with 1-iodo-4-fluorobenzene (146.52 mg), **10** (197.78 mg), copper iodide (33.80 mg), potassium phosphate tribasic (189.00 mg), picolinic acid (32.90 mg) in DMSO, **13d** was obtained (205.87 mg, 63.00% yield). ^1^H NMR: *δ_H_* (300 MHz, CDCl_3_, TMS) 1.62–1.73 (m, 1 H, piperidin-5a-yl), 1.86–1.93(m, 4 H, piperidin-5b-yl, CH_3_), 2.00–2.17 (m, 2 H, piperidin-4-yl), 2.68–2.94 (m, 1 H, piperidin-6a-yl), 2.99–3.13 (m, 1 H, piperidin-2a-yl), 3.63–4.12 (m, 2 H, piperidin-2b-yl, piperidin-6b-yl), 4.36–4.51 (m, 1 H, piperidin-3-yl), 4.70 (s, 2 H, O-CH_2_-phenyl), 5.50 (s, 2 H, N-CH_2_-O), 6.97–7.08 (m, 7 H), 7.10–7.17 (m, 1 H), 7.23–7.27 (m, 1 H), 7.28–7.52 (m, 5 H). ^13 ^C NMR: *δ_C_* (75 MHz, CDCl_3_, TMS) 13.22 (5-CH_3_), 24.61 (piperidin-5-yl), 29.13 (piperidin-4-yl), 70.91 (N-CH_2_-O), 72.33 (O-CH_2_-phenyl), 110.37 (C-5), 116.37 (2 C, Ph), 116.67 (Ph), 119.34 (Ph), 121.10 (Ph), 121.21 (2 C, Ph), 127.51 (2 C, Ph), 127.57 (Ph), 128.23 (2 C, Ph), 130.14 (Ph), 136.93 (2 C, Ph), 138.06 (C-6), 151.20 (C-2), 151.95 (Ph), 157.57 (Ph), 158.16 (Ph), 163.05 (C-4), 169.96 (CO, benzoyl). C (piperidin-2-yl), C (piperidin-3-yl) and C (piperidin-6-yl) cannot be found. HRMS (ESI): m/z [M + H]^+^ Calcd. for [C_31_H_3__0_FN_3_O_5_+H]^+^ 544.2242, found 544.2219.

**13e** following the general procedure, starting with 1-iodo-4-nitrobenzene (164.34 mg), **10** (197.78 mg), copper iodide (33.80 mg), potassium phosphate tribasic (189.00 mg), picolinic acid (32.90 mg) in DMSO, **13e** was obtained (191.40 mg, 55.80% yield). ^1^H NMR: *δ_H_* (300 MHz, CDCl_3_, TMS) 1.73–2.13 (m, 7 H, piperidin-4-yl, piperidin-5-yl, CH_3_), 2.67–2.90 (m, 1 H, piperidin-6a-yl), 2.95–3.12 (m, 1 H, piperidin-2a-yl), 3.57–4.17 (m, 2 H, piperidin-2b-yl, piperidin-6b-yl), 4.35–4.55 (m, 1 H, piperidin-3-yl), 4.70 (s, 2 H, O-CH_2_-phenyl), 5.50 (s, 2 H, N-CH_2_-O), 6.95 (s, 1 H), 7.01–7.10 (m, 2 H), 7.12–7.20 (m, 2 H), 7.21–7.37 (m, 6 H), 7.43–7.56 (m, 1 H), 8.16–8.30 (m, 2 H). ^13 ^C NMR: *δ_C_* (75 MHz, CDCl_3_, TMS) 12.81 (5-CH_3_), 24.44 (piperidin-5-yl), 28.63 (piperidin-4-yl), 52.91 (piperidin-3-yl), 70.48 (N-CH_2_-O), 72.90 (O-CH_2_-phenyl), 110.02 (C-5), 117.35 (2 C, Ph), 118.51 (Ph), 121.28 (Ph), 123.08 (Ph), 125.60 (2 C, Ph), 127.10 (2 C, Ph), 127.18 (Ph), 127.82 (2 C, Ph), 130.23 (Ph), 137.11 (2 C, Ph), 137.59 (C-6), 142.70 (Ph), 150.81 (C-2), 154.81 (Ph), 162.03 (Ph), 162.58 (C-4), 168.96 (CO, benzoyl). C (piperidin-2-yl) and C (piperidin-6-yl) cannot be found. HRMS (ESI): m/z [M + H]^+^ Calcd. for [C_31_H_30_N_4_O_7_+H]^+^ 571.2187, found 571.2191.

**13f** following the general procedure, starting with 1-iodo-3-(trifluoromethyl)benzene (179.53 mg), **10** (197.78 mg), copper iodide (33.80 mg), potassium phosphate tribasic (189.00 mg), picolinic acid (32.90 mg) in DMSO, **13f** was obtained (180.56 mg, 50.60% yield). ^1^H NMR: *δ_H_* (300 MHz, CDCl_3_, TMS) 1.56–2.13 (m, 7 H, piperidin-4-yl, piperidin-5-yl, CH_3_), 2.63–2.94 (m, 1 H, piperidin-6a-yl), 2.95–3.14 (m, 1 H, piperidin-2a-yl), 3.71–4.09 (m, 2 H, piperidin-2b-yl, piperidin-6b-yl), 4.37–4.52 (m, 1 H, piperidin-3-yl), 4.70 (s, 2 H, O-CH_2_-phenyl), 5.50 (s, 2 H, N-CH_2_-O), 6.93 (br. s., 1 H), 7.08 (d, *J* = 6.15 Hz, 2 H), 7.16–7.37 (m, 7 H), 7.37–7.58 (m, 4 H). ^13 ^C NMR: *δ_C_* (75 MHz, CDCl_3_, TMS) 13.21 (5-CH_3_), 23.36 (piperidin-5-yl), 29.11 (piperidin-4-yl), 53.54 (piperidin-3-yl), 70.91 (N-CH_2_-O), 72.31 (O-CH_2_-phenyl), 110.38 (C-5), 115.97 (Ph), 117.53 (Ph), 120.34 (Ph), 120.38 (Ph), 120.46 (2 C, Ph), 122.25 (Ph), 122.34 (Ph), 127.51 (2 C, Ph), 127.57 (Ph), 128.23 (2 C, Ph), 130.43 (Ph), 130.55 (Ph), 137.22 (2 C, Ph), 138.06 (C-6), 151.22 (C-2), 156.96 (Ph), 156.97 (Ph), 163.05 (C-4), 169.67 (CO, benzoyl). C (piperidin-2-yl) and C (piperidin-6-yl) cannot be found. HRMS (ESI): m/z [M + H]^+^ Calcd. for [C_32_H_30_F_3_N_3_O_5_+H]^+^ 594.2211, found 594.2208.

**13g** following the general procedure, starting with 1,2-dichloro-4-iodobenzene (180.11 mg), **10** (197.78 mg), copper iodide (33.80 mg), potassium phosphate tribasic (189.00 mg), picolinic acid (32.90 mg) in DMSO, **13 g** was obtained (173.32 mg, 48.50% yield). ^1^H NMR: *δ_H_* (300 MHz, CDCl_3_, TMS) 1.58–2.05 (m, 7 H, piperidin-4-yl, piperidin-5-yl, CH_3_), 2.57–3.08 (m, 2 H, piperidin-6a-yl, piperidin-2a-yl), 3.55–4.13 (m, 2 H, piperidin-2b-yl, piperidin-6b-yl), 4.32–4.48 (m, 1 H, piperidin-3-yl), 4.65 (s, 2 H, O-CH_2_-phenyl), 5.45 (s, 2 H, N-CH_2_-O), 6.77–6.89 (m, 1 H), 6.95 (br. s., 1 H), 6.98–7.14 (m, 3 H), 7.14–7.46 (m, 8 H). ^13 ^C NMR: *δ_C_* (75 MHz, CDCl_3_, TMS) 12.98 (5-CH_3_), 24.61 (piperidin-5-yl), 28.79 (piperidin-4-yl), 47.28 (piperidin-6-yl), 53.06 (piperidin-3-yl), 60.15 (piperidin-2-yl), 70.71 (N-CH_2_-O), 72.07 (O-CH_2_-phenyl), 109.99 (C-5), 117.28 (Ph), 118.38 (Ph), 120.20 (Ph), 120.72 (Ph), 122.20 (2 C, Ph), 126.90 (Ph), 127.27 (2 C, Ph), 127.36 (Ph), 128.00 (2 C, Ph), 130.21 (Ph), 133.01 (Ph), 137.07 (2 C, Ph), 137.86 (C-6), 151.00 (C-2), 155.51 (Ph), 156.36 (Ph), 162.82 (C-4), 169.32 (CO, benzoyl). HRMS (ESI): m/z [M + H]^+^ Calcd. for [C_31_H_29_Cl_2_N_3_O_5_+H]^+^ 594.1557, found 594.1588.

**13h** following the general procedure, starting with 1,2-difluoro-4-iodobenzene (158.39 mg), **10** (197.78 mg), copper iodide (33.80 mg), potassium phosphate tribasic (189.00 mg), picolinic acid (32.90 mg) in DMSO, **13 h** was obtained (195.13 mg, 57.80% yield). ^1^H NMR: *δ_H_* (300 MHz, CDCl_3_, TMS) 1.48–2.13 (m, 7 H, piperidin-4-yl, piperidin-5-yl, CH_3_), 2.81–3.10 (m, 2 H, piperidin-6a-yl, piperidin-2a-yl), 3.78–4.15 (m, 2 H, piperidin-2b-yl, piperidin-6b-yl), 4.30–4.37 (m, 1 H, piperidin-3-yl), 4.71 (s, 2 H, O-CH_2_-phenyl), 5.45 (s, 2 H, N-CH_2_-O), 6.98–7.06 (m, 2 H), 7.09–7.21 (m, 8 H), 7.33–7.56 (m, 3 H). ^13 ^C NMR: *δ_C_* (75 MHz, CDCl_3_, TMS) 12.61 (5-CH_3_), 24.89 (piperidin-5-yl), 28.53 (piperidin-4-yl), 53.13 (piperidin-3-yl), 70.92 (N-CH_2_-O), 72.19 (O-CH_2_-phenyl), 107.53 (Ph), 109.41 (C-5), 114.86 (Ph), 115.07 (Ph), 117.32 (Ph), 119.78 (Ph), 120.86 (Ph), 127.44 (2 C, Ph), 127.56 (Ph), 128.18 (2 C, Ph), 130.94 (Ph), 137.87 (2 C, Ph), 138.21 (C-6), 145.17 (Ph), 148.35 (Ph), 151.15 (C-2), 152.86 (Ph), 156.78 (Ph), 164.13 (C-4), 168.97 (CO, benzoyl). C (piperidin-2-yl) and C (piperidin-6-yl) cannot be found. HRMS (ESI): m/z [M + H]^+^ Calcd. for [C_31_H_29_F_2_N_3_O_5_+H]^+^ 562.2148, found 562.2122.

**13i** following the general procedure, starting with 1,3-dichloro-5-iodobenzene (180.11 mg), **10** (197.78 mg), copper iodide (33.80 mg), potassium phosphate tribasic (189.00 mg), picolinic acid (32.90 mg) in DMSO, **13i** was obtained (162.96 mg, 45.60% yield). ^1^H NMR: *δ_H_* (300 MHz, CDCl_3_, TMS) 1.68–2.10 (m, 7 H, piperidin-4-yl, piperidin-5-yl, CH_3_), 2.67–2.90 (m, 1 H, piperidin-6a-yl), 2.95–3.17 (m, 1 H, piperidin-2a-yl), 3.63–4.14 (m, 2 H, piperidin-2b-yl, piperidin-6b-yl), 4.33–4.57 (m, 1 H, piperidin-3-yl), 4.71 (s, 2 H, O-CH_2_-phenyl), 5.49 (s, 2 H, N-CH_2_-O), 6.82–7.00 (m, 3 H) 7.05–7.17 (m, 3 H), 7.18–7.39 (m, 6 H) 7.45 (dd, *J* = 8.49, 7.62 Hz, 1 H). ^13 ^C NMR: *δ_C_* (75 MHz, CDCl_3_, TMS) 13.65 (5-CH_3_), 25.30 (piperidin-5-yl), 29.54 (piperidin-4-yl), 53.92 (piperidin-3-yl), 71.34 (N-CH_2_-O), 72.76 (O-CH_2_-phenyl), 110.84 (C-5), 117.91 (2 C, Ph), 118.46 (Ph), 121.36 (Ph), 123.39 (Ph), 124.26 (Ph), 127.95 (2 C, Ph), 128.02 (Ph), 128.66 (2 C, Ph), 130.95 (Ph), 136.18 (2 C, Ph), 137.66 (2 C, Ph), 138.49 (C-6), 151.65 (C-2), 156.42 (Ph), 158.47 (Ph), 163.48 (C-4), 169.96 (CO, benzoyl). C (piperidin-2-yl), C (piperidin-6-yl) cannot be found. HRMS (ESI): m/z [M + H]^+^ Calcd. for [C_31_H_29_Cl_2_N_3_O_5_+H]^+^ 594.2211, found 594.1580.

**13j** following the general procedure, starting with 1,3-difluoro-5-iodobenzene (158.39 mg), **10** (197.78 mg), copper iodide (33.80 mg), potassium phosphate tribasic (189.00 mg), picolinic acid (32.90 mg) in DMSO, **13j** was obtained (154.62 mg, 45.80% yield). ^1^H NMR: *δ_H_* (300 MHz, CDCl_3_, TMS) 1.75–2.10 (m, 7 H, piperidin-4-yl, piperidin-5-yl, CH_3_), 2.62–2.92 (m, 1 H, piperidin-6a-yl), 2.94–3.16 (m, 1 H, piperidin-2a-yl), 3.63–4.16 (m, 2 H, piperidin-2b-yl, piperidin-6b-yl), 4.34–4.55 (m, 1 H, piperidin-3-yl), 4.70 (s, 2 H, O-CH_2_-phenyl), 5.50 (s, 2 H, N-CH_2_-O), 6.45–6.62 (m, 3 H), 6.93 (br. s., 1 H), 7.05–7.17 (m, 2 H), 7.21–7.37 (m, 6 H), 7.38–7.53 (m, 1 H). ^13 ^C NMR: *δ_C_* (75 MHz, CDCl_3_, TMS) 13.21 (5-CH_3_), 24.85 (piperidin-5-yl), 29.08 (piperidin-4-yl), 53.58 (piperidin-3-yl), 70.89 (N-CH_2_-O), 72.30 (O-CH_2_-phenyl), 99.04 (Ph), 101.10 (Ph), 102.25 (Ph) 110.40 (C-5), 118.29 (Ph), 121.15 (Ph), 122.99 (Ph), 127.50 (2 C, Ph), 127.56 (Ph), 128.21 (2 C, Ph), 130.46 (Ph), 137.25 (2 C, Ph), 138.04 (C-6), 151.22 (C-2), 155.84 (Ph), 161.84 (Ph), 163.02 (C-4), 165.14 (Ph), 165.34 (Ph), 169.54 (CO, benzoyl). C (piperidin-2-yl), C (piperidin-6-yl) cannot be found. HRMS (ESI): m/z [M + H]^+^ Calcd. for [C_31_H_29_F_2_N_3_O_5_+H]^+^ 562.2148, found 562.2155.

**13k** following the general procedure, starting with 1-iodo-3,5-bis(trifluoromethyl)benzene (224.40 mg), **10** (197.78 mg), copper iodide (33.80 mg), potassium phosphate tribasic (189.00 mg), picolinic acid (32.90 mg) in DMSO, **13k** was obtained (165.45 mg, 41.60% yield). ^1^H NMR: *δ_H_* (300 MHz, CDCl_3_, TMS) 1.63–2.12 (m, 7 H, piperidin-4-yl, piperidin-5-yl, CH_3_), 2.64–2.89 (m, 1 H, piperidin-6a-yl), 2.96–3.19 (m, 1 H, piperidin-2a-yl), 3.66–4.17 (m, 2 H, piperidin-2b-yl, piperidin-6b-yl), 4.34–4.55 (m, 1 H, piperidin-3-yl), 4.70 (s, 2 H, O-CH_2_-phenyl), 5.49 (s, 2 H, N-CH_2_-O), 6.94 (s, 1 H), 7.07–7.17 (m, 2 H), 7.17–7.37 (m, 6 H), 7.43 (s, 2 H), 7.44–7.53 (m, 1 H), 7.61 (s, 1 H). ^13 ^C NMR: *δ_C_* (75 MHz, CDCl_3_, TMS) 13.13 (5-CH_3_), 24.85 (piperidin-5-yl), 29.01 (piperidin-4-yl), 53.28 (piperidin-3-yl), 70.85 (N-CH_2_-O), 72.25 (O-CH_2_-phenyl), 110.35 (C-5), 116.89 (Ph), 118.18 (Ph), 118.58 (2 C, Ph), 120.89 (2 C, Ph), 123.41 (2 C, Ph), 127.47 (2 C, Ph), 127.53 (Ph), 128.17 (2 C, Ph), 130.73 (Ph), 133.08 (Ph), 133.73 (Ph), 137.57 (2 C, Ph), 138.01 (C-6), 151.19 (C-2), 155.52 (Ph), 157.87 (Ph), 162.97 (C-4), 169.28 (CO, benzoyl). C (piperidin-2-yl) and C (piperidin-6-yl) cannot be found. HRMS (ESI): m/z [M + H]^+^ Calcd. for [C_33_H_29_F_6_N_3_O_5_+H]^+^ 662.2084, found 662.2086.

*General procedure for substituted 5-methyl-1–(1-(3-phenoxybenzoyl)piperidin-3-yl)pyrimidine-2,4(1H,3H)-dione* (**4a**–**4b**). **13a**–**13k** was dissolved in 2–4 ml trifluoroacetic acid and heated at 73 °C for 4 h. The reaction mixture was cooled to room temperature and the trifluoroacetic acid was removed *in vacuo*. The residue was dissolved in 2 ml methanol, and the mixture was neutralised with saturated NaHCO_3_ to pH 5–6. The generated solid was filtered and the filtrate was concentrated and purified by column chromatography (CH_2_Cl_2_–MeOH, 20: 1 v/v) to give solid **4a**–**4k** (70–85% yield), which was characterised by ^1^H and ^13 ^C-NMR, and ESI-HRMS.

**4a** ^1^H NMR: *δ_H_* (300 MHz, DMSO-*d6*, TMS) 1.47–1.60 (m, 1 H, piperidin-5a-yl), 1.74–1.93 (m, 6 H, piperidin-4-yl, piperidin-5b-yl, CH_3_), 2.79–2.98 (m, 7 H, piperidin-6a-yl, N(CH_3_)_2_), 3.07–3.19 (m, 1 H, piperidin-2a-yl), 3.90–4.14 (m, 2 H, piperidin-2b-yl, piperidin-6b-yl), 4.26–4.36 (m, 1 H, piperidin-3-yl), 6.27 (dd, *J* = 7.76, 1.90 Hz, 1 H), 6.37 (t, *J* = 2.34 Hz, 1 H), 6.52 (dd, *J* = 8.20, 2.05 Hz, 1 H), 6.92–6.96 (m, 1 H), 7.00–7.07 (m, 1 H), 7.10 (d, *J* = 7.62 Hz, 1 H), 7.16 (t, *J* = 8.20 Hz, 1 H), 7.37–7.42 (m, 1 H), 7.50 (s, 1 H), 10.92 (br. s., 1 H, 3-NH). ^13 ^C NMR: *δ_C_* (75 MHz, DMSO-*d6*, TMS) 12.09 (5-CH_3_), 24.54 (piperidin-5-yl), 28.14 (piperidin-4-yl), 50.64 (piperidin-3-yl), 103.32 (Ph), 106.57 (Ph), 108.25 (Ph), 109.03 (C-5), 115.97 (Ph), 118.98 (Ph), 120.95 (Ph), 128.40 (Ph), 130.17 (Ph), 135.54 (Ph), 137.51 (C-6), 150.71 (Ph), 151.98 (C-2), 156.77 (Ph), 157.28 (Ph), 163.58 (C-4), 168.44 (CO, benzoyl). C (N(CH_3_)_2_), C (piperidin-2-yl) and C (piperidin-2-yl) cannot be found. HRMS (ESI): m/z [M + H]^+^ Calcd. for [C_25_H_28_N_4_O_4_+H]^+^ 449.2184, found 449.2208.

**4b** ^1^H NMR: *δ_H_* (300 MHz, DMSO-*d6*, TMS) 1.44–1.62 (m, 1 H, piperidin-5a-yl), 1.70–2.05 (m, 6 H, piperidin-4-yl, piperidin-5b-yl, CH_3_), 2.80–2.96 (m, 1 H, piperidin-6a-yl), 3.09–3.24 (m, 1 H, piperidin-2a-yl), 3.73 (s, 3 H, OCH_3_), 3.82–4.18 (m, 2 H, piperidin-2b-yl, piperidin-6b-yl), 4.24–4.38 (m, 1 H, piperidin-3-yl), 6.53–6.63 (m, 2 H), 6.69–6.77 (m, 1 H), 6.96–7.02 (m, 1 H), 7.04–7.11 (m, 1 H), 7.11–7.18 (m, 1 H), 7.27 (t, *J* = 8.05 Hz, 1 H), 7.38–7.47 (m, 1 H), 7.50 (d, *J* = 0.88 Hz, 1 H), 10.92 (br. s., 1 H, 3-NH). ^13^C NMR: *δ_C_* (75 MHz, DMSO-*d6*, TMS) 12.11 (5-CH_3_), 24.72 (piperidin-5-yl), 28.12 (piperidin-4-yl), 50.41 (piperidin-3-yl), 55.29 (OCH_3_), 105.10 (Ph), 109.04 (C-5), 109.68 (Ph), 110.95 (Ph), 116.60 (Ph), 119.50 (Ph), 121.57 (Ph), 130.26 (Ph), 130.63 (Ph), 137.63 (C-6), 150.73 (C-2), 156.67 (Ph), 157.25 (Ph), 160.76 (Ph), 163.60 (C-4), 168.33 (CO, benzoyl). C (piperidin-2-yl), C (piperidin-6-yl) and C (Ph-CO-N) cannot be found. HRMS (ESI): m/z [M + H]^+^ Calcd. for [C_24_H_25_N_3_O_5_+H]^+^ 436.1867, found 436.1899.

**4c** ^1^H NMR: *δ_H_* (300 MHz, DMSO-*d6*, TMS) 1.45–1.66 (m, 1 H, piperidin-5a-yl), 1.74–2.02 (m, 6 H, piperidin-4-yl, piperidin-5b-yl, CH_3_), 2.80–2.97 (m, 1 H, piperidin-6a-yl), 3.07–3.20 (m, 1 H, piperidin-2a-yl), 3.82–4.18 (m, 2 H, piperidin-2b-yl, piperidin-6b-yl), 4.25–4.39 (m, 1 H, piperidin-3-yl), 6.98–7.02 (m, 1 H), 7.03–7.12 (m, 3 H), 7.16 (d, *J* = 7.61 Hz, 1 H), 7.37–7.47 (m, 3 H), 7.50 (s, 1 H), 10.93 (br. s., 1 H, 3-NH). ^13 ^C NMR: *δ_C_* (75 MHz, DMSO-*d6*, TMS) 12.09 (5-CH_3_), 24.43 (piperidin-5-yl), 28.05 (piperidin-4-yl), 50.56 (piperidin-3-yl), 109.01 (C-5), 116.78 (Ph), 119.68 (Ph), 120.66 (2 C, Ph), 121.91 (Ph), 127.60 (Ph), 129.98 (2 C, Ph), 130.38 (Ph), 137.46 (Ph), 137.80 (C-6), 150.71 (C-2), 155.19 (Ph), 156.36 (Ph), 163.58 (C-4), 168.22 (CO, benzoyl). C (piperidin-2-yl) and C (piperidin-6-yl) cannot be found. HRMS (ESI): m/z [M + H]^+^ Calcd. for [C_23_H_22_ClN_3_O_4_+H]^+^ 440.1372, found 440.1380.

**4d** ^1^H NMR: *δ_H_* (300 MHz, DMSO-*d6*, TMS) 1.45–1.61 (m, 1 H, piperidin-5a-yl), 1.73–2.00 (m, 6 H, piperidin-4-yl, piperidin-5b-yl, CH_3_), 2.80–2.94 (m, 1 H, piperidin-6a-yl), 3.09–3.21 (m, 1 H, piperidin-2a-yl), 3.84–4.16 (m, 2 H, piperidin-2b-yl, piperidin-6b-yl), 4.22–4.39 (m, 1 H, piperidin-3-yl), 6.93–6.98 (m, 1 H), 7.01–7.12 (m, 3 H), 7.14 (d, *J* = 1.17 Hz, 1 H), 7.15–7.23 (m, 2 H), 7.41 (t, *J* = 11.72 Hz, 1 H), 7.50 (d, *J* = 0.88 Hz, 1 H), 10.93 (s, 1 H, 3-NH). ^13 ^C NMR: *δ_C_* (75 MHz, DMSO-*d6*, TMS) 12.12 (5-CH_3_), 24.69 (piperidin-5-yl), 28.15 (piperidin-4-yl), 50.56 (piperidin-3-yl), 109.06 (C-5), 116.05 (Ph), 116.57 (Ph), 116.89 (Ph), 118.89 (Ph), 121.13 (Ph), 121.25 (Ph), 121.39 (Ph), 130.31 (Ph), 137.50 (Ph), 137.69 (C-6), 150.75 (C-2), 152.07 (Ph), 156.90 (Ph), 157.26 (Ph), 163.61 (C-4), 168.35 (CO, benzoyl). C (piperidin-2-yl) and C (piperidin-6-yl) cannot be found. HRMS (ESI): m/z [M + H]^+^ Calcd. for [C_23_H_22_FN_3_O_4_+H]^+^ 424.1667, found 424.1655.

**4e** ^1^H NMR: *δ_H_* (300 MHz, DMSO-*d6*, TMS) 1.47–1.66 (m, 1 H, piperidin-5a-yl), 1.71–2.08 (m, 6 H, piperidin-4-yl, piperidin-5b-yl, CH_3_), 2.76–2.99 (m, 1 H, piperidin-6a-yl), 3.08–3.22 (m, 1 H, piperidin-2a-yl), 3.80–4.23 (m, 2 H, piperidin-2b-yl, piperidin-6b-yl), 4.24–4.46 (m, 1 H, piperidin-3-yl), 7.11–7.25 (m, 4 H), 7.28 (d, *J* = 7.62 Hz, 1 H), 7.46–7.61 (m, 2 H), 8.18–8.32 (m, 2 H), 10.93 (br. s., 1 H, 3-NH). ^13 ^C NMR: *δ_C_* (75 MHz, DMSO-*d6*, TMS) 12.09 (5-CH_3_), 24.66 (piperidin-5-yl), 28.11 (piperidin-4-yl), 50.50 (piperidin-3-yl), 109.03 (C-5), 117.82 (2 C, Ph), 118.63 (Ph), 121.38 (Ph), 123.53 (Ph), 126.21 (2 C, Ph), 130.79 (Ph), 137.45 (Ph), 138.24 (C-6), 142.53 (Ph), 150.73 (C-2), 154.39 (Ph), 162.45 (Ph), 163.60 (C-4), 167.99 (CO, benzoyl). C (piperidin-2-yl) and C (piperidin-6-yl) cannot be found. HRMS (ESI): m/z [M + H]^+^ Calcd. for [C_23_H_22_N_4_O_6_+H]^+^ 451.1612, found 451.1636.

**4f** ^1^H NMR: *δ_H_* (300 MHz, DMSO-*d6*, TMS) 1.44–1.65 (m, 1 H, piperidin-5a-yl), 1.69–2.07 (m, 6 H, piperidin-4-yl, piperidin-5b-yl, CH_3_), 2.81–2.97 (m, 1 H, piperidin-6a-yl), 3.13 (t, *J* = 11.86 Hz, 1 H, piperidin-2a-yl), 3.78–4.21 (m, 2 H, piperidin-2b-yl, piperidin-6b-yl), 4.23–4.43 (m, 1 H, piperidin-3-yl), 7.08 (d, *J* = 1.46 Hz, 1 H), 7.11–7.17 (m, 1 H), 7.22 (d, *J* = 7.91 Hz, 1 H), 7.29–7.37 (m, 2 H), 7.43–7.54 (m, 3 H), 7.62 (t, *J* = 7.83 Hz, 1 H), 10.92 (br. s., 1 H, 3-NH). ^13 ^C NMR: *δ_C_* (75 MHz, DMSO-*d6*, TMS) 12.11 (5-CH_3_), 24.72 (piperidin-5-yl), 28.12 (piperidin-4-yl), 109.04 (C-5), 115.16 (Ph), 117.38 (Ph), 120.18 (Ph), 121.86 (Ph), 122.54 (2 C, Ph), 125.48 (CF_3_), 130.61 (Ph), 130.99 (Ph), 131.51 (Ph), 137.37 (Ph), 137.98 (C-6), 150.73 (C-2), 155.71 (Ph), 157.00 (Ph), 163.60 (C-4), 168.13 (CO, benzoyl). C (piperidin-2-yl), C (piperidin-3-yl) and C (piperidin-6-yl) cannot be found. HRMS (ESI): m/z [M + H]^+^ Calcd. for [C_24_H_22_F_3_N_3_O_4_+H]^+^ 474.1635, found 474.1624.

**4g** ^1^H NMR: *δ_H_* (300 MHz, DMSO-*d6*, TMS) 1.44–1.67 (m, 1 H, piperidin-5a-yl), 1.73–2.00 (m, 6 H, piperidin-4-yl, piperidin-5b-yl, CH_3_), 2.80–2.97 (m, 1 H, piperidin-6a-yl), 3.11–3.21 (m, 1 H, piperidin-2a-yl), 3.73–4.21 (m, 2 H, piperidin-2b-yl, piperidin-6b-yl), 4.25–4.38 (m, 1 H, piperidin-3-yl), 7.03 (dd, *J* = 8.93, 2.78 Hz, 1 H), 7.06–7.09 (m, 1 H), 7.11–7.17 (m, 1 H), 7.17–7.24 (m, 1 H), 7.29 (d, *J* = 2.64 Hz, 1 H), 7.45 (d, *J* = 7.91 Hz, 1 H), 7.48–7.53 (m, 1 H), 7.59 (d, *J* = 9.08 Hz, 1 H), 10.92 (s, 1 H, 3-NH). ^13 ^C NMR: *δ_C_* (75 MHz, DMSO-*d6*, TMS) 12.12 (5-CH_3_), 24.72 (piperidin-5-yl), 28.20 (piperidin-4-yl), 50.15 (piperidin-3-yl), 109.06 (C-5), 117.21 (Ph), 119.04 (Ph), 120.11 (Ph), 120.70 (Ph), 122.57 (Ph), 125.74 (Ph), 130.58(Ph), 131.69 (Ph), 132.06 (Ph), 137.50 (Ph), 137.97 (C-6), 150.75 (C-2), 155.71 (Ph), 156.01 (Ph), 163.63 (C-4), 168.15 (CO, benzoyl). C (piperidin-2-yl) and C (piperidin-6-yl) cannot be found. HRMS (ESI): m/z [M + H]^+^ Calcd. for [C_23_H_21_Cl_2_N_3_O_4_+H]^+^ 474.0982, found 474.0987.

**4h** ^1^H NMR: *δ_H_* (300 MHz, DMSO-*d6*, TMS) 1.46–1.63 (m, 1 H, piperidin-5a-yl), 1.73–2.02 (m, 6 H, piperidin-4-yl, piperidin-5b-yl, CH_3_), 2.89 (t, *J* = 12.30 Hz, 1 H, piperidin-6a-yl), 3.10–3.22 (m, 1 H, piperidin-2a-yl), 3.82–4.17 (m, 2 H, piperidin-2b-yl, piperidin-6b-yl), 4.25–4.41 (m, 1 H, piperidin-3-yl), 6.83–6.91 (m, 1 H), 6.98–7.04 (m, 1 H), 7.05–7.21 (m, 3 H), 7.33–7.56 (m, 3 H) 10.93 (br. s., 1 H, 3-NH). ^13 ^C NMR: *δ_C_* (75 MHz, DMSO-*d6*, TMS) 12.14 (5-CH_3_), 24.73 (piperidin-5-yl), 28.20 (piperidin-4-yl), 50.53 (piperidin-3-yl), 109.10 (C-5), 109.36 (Ph), 115.59 (Ph), 116.58 (Ph), 118.19 (Ph), 118.43 (Ph), 119.47 (Ph), 122.05 (Ph), 130.47 (Ph), 137.53 (Ph), 137.83 (C-6), 148.24 (Ph), 150.78 (C-2), 152.41 (Ph), 156.52 (Ph), 163.66 (C-4), 168.27 (CO, benzoyl). C (piperidin-2-yl) and C (piperidin-6-yl) cannot be found. HRMS (ESI): m/z [M + H]^+^ Calcd. for [C_23_H_21_F_2_N_3_O_4_+H]^+^ 442.1573, found 442.1566.

**4i** ^1^H NMR: *δ_H_* (300 MHz, DMSO-*d6*, TMS) 1.47–1.60 (m, 1 H, piperidin-5a-yl), 1.72–2.02 (m, 6 H, piperidin-4-yl, piperidin-5b-yl, CH_3_), 2.90 (t, *J* = 12.33 Hz, 1 H, piperidin-6a-yl), 3.87–4.12 (m, 2 H, piperidin-2b-yl, piperidin-6b-yl), 4.26–4.32 (m, 1 H, piperidin-3-yl), 7.04 (d, *J* = 2.05 Hz, 1 H), 7.08–7.12 (m, 1 H), 7.12–7.21 (m, 2 H), 7.21–7.27 (m, 1 H), 7.28–7.32 (m, 1 H) 7.49 (t, *J* = 7.98 Hz, 2 H), 10.90 (br. s., 1 H). H (piperidin-2a-yl) was hidden in water peak. ^13 ^C NMR: *δ_C_* (75 MHz, DMSO-*d6*, TMS) 11.48 (5-CH_3_), 24.03 (piperidin-5-yl), 27.82 (piperidin-4-yl), 51.28 (piperidin-3-yl), 108.81 (C-5), 117.09 (2 C, Ph), 117.36 (Ph), 120.14 (Ph), 122.66 (Ph), 122.98 (Ph), 130.26 (Ph), 134.73 (Ph), 137.08 (2 C, Ph), 137.79 (C-6), 150.38 (C-2), 154.94 (Ph), 157.97 (Ph), 163.14 (C-4), 167.92 (CO, benzoyl). C (piperidin-2-yl) and C (piperidin-6-yl) cannot be found. HRMS (ESI): m/z [M + H]^+^ Calcd. for [C_23_H_21_Cl_2_N_3_O_4_+H]^+^ 474.0982, found 474.0022.

**4j** ^1^H NMR: *δ_H_* (300 MHz, DMSO-*d6*, TMS) 1.45–1.65 (m, 1 H, piperidin-5a-yl), 1.72–2.10 (m, 6 H, piperidin-4-yl, piperidin-5b-yl, CH_3_), 2.90 (t, *J* = 12.78 Hz, 1 H, piperidin-6a-yl), 3.08–3.24 (m, 1 H, piperidin-2a-yl), 3.81–4.21 (m, 2 H, piperidin-2b-yl, piperidin-6b-yl), 4.26–4.40 (m, 1 H, piperidin-3-yl), 6.65–6.79 (m, 2 H), 6.91 (tt, *J* = 7.59 Hz, 4.35 Hz, 1 H), 7.07–7.13 (m, 1 H), 7.13–7.21 (m, 1 H), 7.24 (dt, *J* = 7.62, 1.17 Hz, 1 H), 7.46 (s, 1 H), 7.49–7.53 (m, 1 H), 10.92 (br. s., 1 H, 3-NH). ^13 ^C NMR: *δ_C_* (75 MHz, DMSO-*d6*, TMS) 12.11 (5-CH_3_), 24.61 (piperidin-5-yl), 28.23 (piperidin-4-yl), 99.21 (Ph), 102.19 (Ph), 102.57 (Ph), 109.04 (C-5), 117.70 (Ph), 120.49 (Ph), 122.95 (Ph), 130.61 (Ph), 137.59 (Ph), 138.08 (C-6), 150.75 (C-2), 155.11 (Ph), 158.74 (Ph), 161.44 (Ph), 163.60 (C-4), 164.71 (Ph), 168.07 (CO, benzoyl). C (piperidin-2-yl), C (piperidin-3-yl) and C (piperidin-6-yl) cannot be found. HRMS (ESI): m/z [M + H]^+^ Calcd. for [C_23_H_21_F_2_N_3_O_4_+H]^+^ 442.1573, found 442.1604.

**4k** ^1^H NMR: *δ_H_* (300 MHz, DMSO-*d6*, TMS) 1.48–1.65 (m, 1 H, piperidin-5a-yl), 1.67–2.14 (m, 6 H, piperidin-4-yl, piperidin-5b-yl, CH_3_), 2.80–2.96 (m, 1 H, piperidin-6a-yl), 3.11–3.23 (m, 1 H, piperidin-2a-yl), 3.74–4.20 (m, 2 H, piperidin-2b-yl, piperidin-6b-yl), 4.23–4.42 (m, 1 H, piperidin-3-yl), 7.11–7.34 (m, 3 H), 7.43–7.58 (m, 2 H), 7.63 (s, 2 H), 7.78 (s, 1 H), 10.91 (br. s., 1 H, 3- NH). ^13 ^C NMR: *δ_C_* (75 MHz, DMSO-*d6*, TMS) 12.16 (5-CH_3_), 24.47 (piperidin-5-yl), 28.23 (piperidin-4-yl), 50.62 (piperidin-3-yl), 109.18 (C-5), 116.92 (Ph), 117.85 (Ph), 119.15 (2 C, Ph), 120.75 (Ph), 121.12 (Ph), 123.45 (Ph), 124.73 (Ph), 131.02 (Ph), 131.94 (Ph), 132.38 (Ph), 137.53 (Ph), 138.18 (C-6), 150.82 (C-2), 154.97 (Ph), 158.07 (Ph), 163.74 (C-4), 168.15 (CO, benzoyl). C (piperidin-2-yl) and C (piperidin-6-yl) cannot be found. HRMS (ESI): m/z [M + H]^+^ Calcd. for [C_25_H_21_F_6_N_3_O_4_+H]^+^ 542.1509, found 542.1508.

### 2.2. Molecular modelling

The modelling was conducted using AutoDock vina and AutodockTools-1.5.6^26^ with publicly available X-ray structure of the *Mtb*TMPK (PDB entry 1G3U^9^ and 5NQ5^27^). All PDB files of ligand were generated in ChemDraw 3 D 16.0 after being minimised the energy. Centred on *Mtb*TMPK active site PHE70 CE2, the prepared PDBQT files of ligands and receptors were docked using a grid spacing of 0.375 and 60 × 60 × 60 number of grid points. Each ligand was docked three times in autodock vina by Lamarckian 4.2 method, affording total 60 possible conformations. All vinadock results were viewed in Chimaera, further analysed and validated through the predicted free energy in combination with possible interactions in LigPlus.

### 2.3. *Mtb*TMPK assay

*Mtb*TMPK was overexpressed in *Escherichia coli* and purified by a two-step procedure as previously described^8^. *Mtb*TMPK activities of all compounds were measured using the spectrophotometric assay reported by Blondin et al^28^. All compounds were tested at varying concentration from 0.006 to 0.12 mM with constant concentrations of ATP (0.5 mM) and dTMP (0.05 mM). The studies were done at 30 °C in a medium containing 50 mM Tris–HCl, 2 mM MgCl_2_, pH 7.4, 50 mM KCl, 1 mM phosphoenol pyruvate, 0.2 mM NADH and two units each of coupling enzymes (lactate dehydrogenase, pyruvate kinase and nucleoside diphosphate kinase). The absorbance at 334 nm was monitored in an Eppendorf ECOM 6122 photometer. IC_50_ were calculated using Kaleidagraph as previously described^27^.

### 2.4. Whole cell activity against *Mtb*

MIC_50_ against mycobacteria was evaluated by a 2-fold serial dilution of the tested compounds. The *in vitro* antimycobacterial assay was based on a method in which a luminescent *M. tuberculosis* H37Ra strain Lehmann & Neumann (ATCC 25177) transformed with pSMTB1 luciferase reporter plasmid is used. Each compound was dissolved in DMSO (Sigma-Aldrich) to prepare the stock solution at concentration of 10 mM. Serial dilutions were made in Middlebrook 7H9 broth and 10% OADC (oleic acid, albumin, dextrose, catalase). Volumes of 20 μl of the serial dilutions were added in triplicate to flat-bottomed 96-well plates. By thawing and dissolving a frozen Mycobacteria pellet in 7H9-10% OADC, the bacterial suspension was made. To eliminate clumps, the dissolved pellet was passed through a 5.0 μM filter (Millipore) and left for 1 h to recover at 37 °C, 5% CO_2_. Subsequently, the suspension was diluted in complete 7H9 broth to obtain 50,000 Relative Light Units (RLU)/ml and a volume of 180 μl of bacteria was added to each well. After 7 days of incubation, the bacterial replication was analysed by luminometry. The bacterial suspension from each well was collected and transferred to a black 96-well plate to evade cross luminescence between wells. The luminescent signal was evoked by addition of the substrate for the bacterial luciferase, 1% *n*-decanal in ethanol to each well by the Discover multi-plate reader from Promega and the light emission in each well was measured. Using the plot of the % viability as a function of compound concentration, MIC_50_ values were determined.

## 3. Results and discussion

### 3.1. Chemistry

**Scheme 1.**
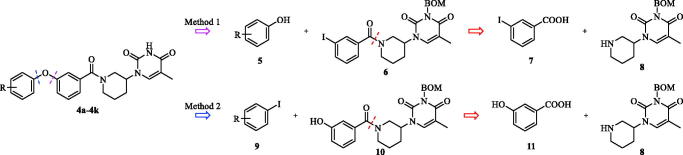
Two divergent routes used for the synthesis of compounds **4a**–**4k**.

For the synthesis of the target compounds, two divergent synthesis routes were considered based on a disconnection of the ether bond between the C and D ring (Scheme 1).

**Scheme 2.**
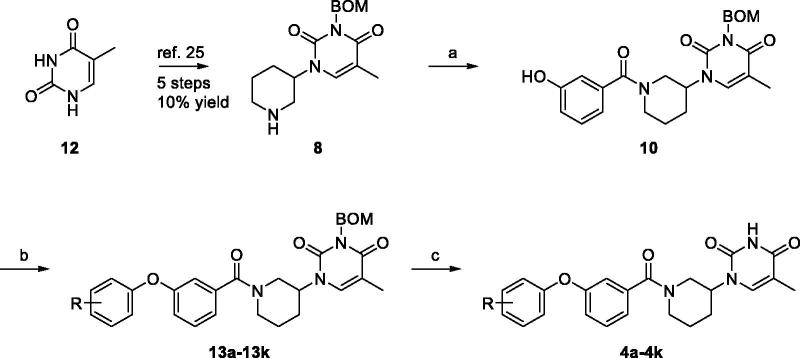
Synthesis of compound **4a**–**4k**. Reagents and conditions: (a) N,N-bis(isopropyl) carbondiimide, DMAP, CH_2_Cl_2_, overnight, 62% yield; (b) CuI, K_3_PO_4_, DMSO, picolinic acid, 90 °C, overnight, 40.00–70.00% yield; (c) TFA, 73 °C, 4 h, 70–85% yield.

The target compounds **4a**–**4k** may be obtained by Ullmann coupling of either substituted phenol (**5**) with 3-iodobenzamide **6** (Method 1) or substituted iodobenzene (**9**) with phenol **10** (Method 2). We experienced that Method 1 failed to produce the coupling product in the case of electron-withdrawing substituents on the phenol ring. Except for analogue **4a**, all analogues were prepared according to Method 2 as detailed Scheme 2.

Intermediate **8** was synthesised as previously reported^25^. Briefly, thymine (**12**) was converted to N3-benzyloxymethyl-protected thymine in three steps. Next, a substitution reaction with tert-butyl 3-((methylsulfonyl)oxy)piperidine-1-carboxylate, followed by BOC-deprotection with trifluoroacetic acid afforded 3-piperidylthymine **8**. Amidation of **8** with 3-hydroxybenzoic acid (**11**) gave compound **10**. Ullmann coupling of **10** with the appropriately substituted iodobenzene, followed by BOM-deprotection afforded the desired substituted (1–(3-phenoxybenzoyl)piperidin-4-yl)thymines **4a**–**4k**.

### 3.2. Docking

**Figure 3.**
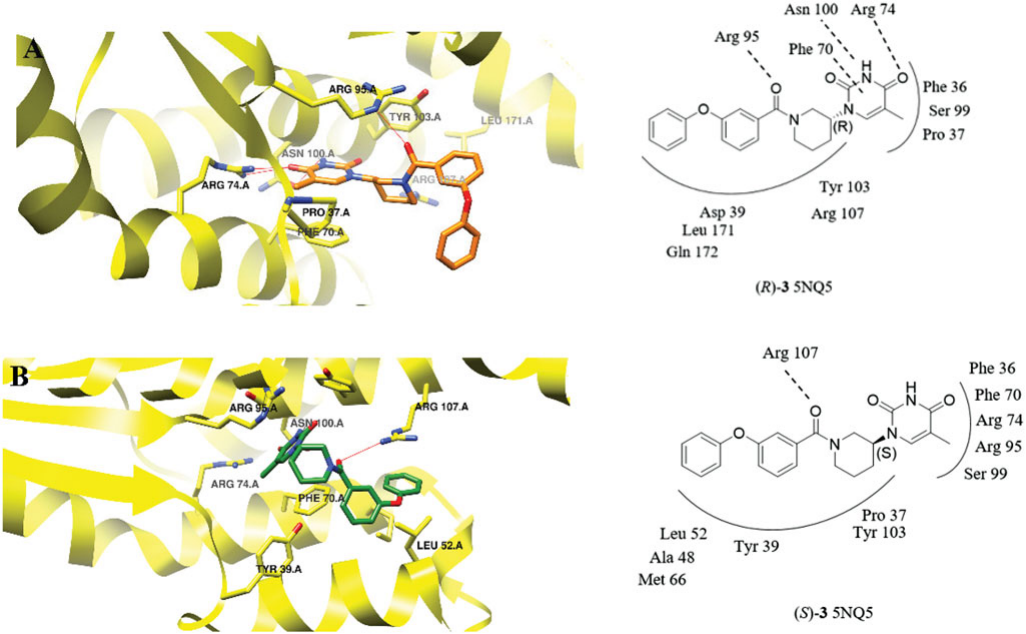
(*R*)- and (*S*)-**3** docked in the active site of *Mtb*TMPK (PDB 5NQ5). *Mtb*TMPK is shown in a yellow cartoon representation with selected side chains labelled and shown as sticks with carbon atoms coloured yellow. Ligands are drawn in stick representation with carbon atoms in orange ((*R*)-3) and green ((*S*)-3), hydrogen-bonding interactions are shown as red lines.

**Figure 4.**
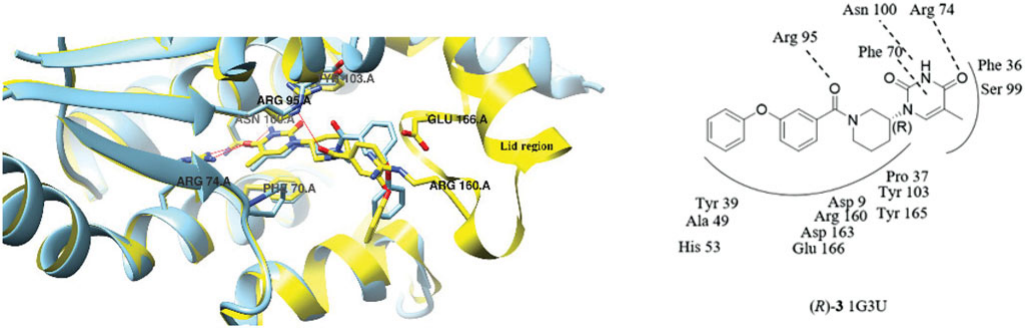
Structure overlay of compound (*R*)-**3** docked into *Mtb*TMPK PDB 1G3U (yellow) and PDB 5NQ5 (blue), hydrogen-bonding interactions are shown as red dashed lines.

To gain insight into their putative binding mode, putative analogues were docked in the target enzyme using the crystal structure of *Mtb*TMK in complex with (*S*)-**1** (PDB code 5NQ5)^27^. Docking indicated that the (*R)*-**3** is better accommodated than its (*S*)-enantiomer (Figure 3). The thymine moiety of (*R)*-**3** adopts an identical pose as that of (*S*)-**1** and is involved in two hydrogen bonds between its NH and C(4)O groups and the side chains of Asn100 and Arg74, as well as a π–π stacking interactions with Phe-70. By protruding out of the active site, the meta-piperidine ring forms a π-alkyl interaction with the aromatic side chain of Tyr 103. Importantly, in the (*R*)-enantiomer the oxygen atom of the carbonyl that connects ring B and ring C is expected to form a hydrogen bond with Arg-95 (Figure 3(A)), which justifies our choice to connect ring C to the piperidine ring B through an amide bond. Furthermore, through various hydrophobic interactions ring C and ring D contribute to the overall affinity. The putative binding mode of the (*S)*-enantiomer is quite different (Figure 3(B)). Although a hydrogen bond may be formed between the oxygen atom of the carbonyl connecting ring B and ring C and Arg-107, the loss of the hydrogen bonds between N^3^ and C^4^ groups of thymine ring and the side chains of Asn100 and Arg7, as well as the π–π stacking interaction between pyrimidine ring and Phe-70 results in a less promising docking score.

(*R*)-**3** was also docked starting from the co-crystal structure of the natural substrate TMP (PDB code 1G3U)^9^. The resulting docking pose of (*R*)-**3** in *Mtb*TMPK is superimposed with the one described above (Figure 4). The *Mtb*TMPK conformation is highly similar except for the flexible Lid region, leading to slightly more encouraging docking score due to extra pronounced hydrophobic interactions exhibited by the C and D ring of (*R*)-**3**. Considering the envisioned analogues are highly related to the structure of (*S*)-**1**, the co-crystal structure of *Mtb*TMPK in complex with (*S*)-**1** was used for modelling.

**Figure 5.**
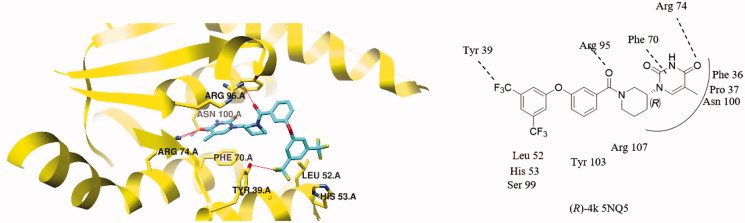
Compound **4k** docked in the active site of *Mtb*TMPK (PDB 5NQ5). The protein is shown in a yellow cartoon representation with selected side chains labelled and shown as sticks and **4k** is drawn in stick representation with carbon atoms in cyan. Hydrogen-bonding interactions are shown as red lines.

Docking analysis starting from the 5NQ5 co-crystal structure further indicated that judicious substituents of the D-ring might lead to extra interactions with the enzyme. A trifluoromethyl substituent in meta position (as in **4k**) may establish a hydrogen bond with Tyr 39 (Figure 5). However, the consequent distortion of the D-ring leads to a less favourable docking score (Table 1).

**Table 1.** Docking results of compounds **4a**–**4k**, as well as their activities against *Mtb*TMPK and avirulent *M. tuberculosis.*


Compound	R	Docking score	IC_50_*_Mtb_*_TMPK_ (μM)	MIC_50 H37Ra_ (μM)^a^
**4a**	3-N(CH_3_)_2_	−8.5	39 ± 3	>64
**4b**	3-OCH_3_	−8.5	15 ± 1	>64
**4c**	4-Cl	−8.8	39 ± 19	>64
**4d**	4-F	−8.8	18 ± 1	>64
**4e**	4-NO_2_	−9.0	30 ± 3	>64
**4f**	3-CF_3_	−9.2	53 ± 12	>64
**4g**	3,4-Cl_2_	−8.8	18 ± 1	35.8
**4h**	3,4-F_2_	−8.8	23 ± 1	>64
**4i**	3,5-Cl_2_	−8.7	13 ± 1	>64
**4j**	3,5-F_2_	−8.8	31 ± 2	>64
**4k**	3,5-(CF_3_)_2_	−8.2	158 ± 89	>64
**3**	—	−8.7	28 ± 2	>64

a Compounds were tested on H37Ra strain, measured as the half-maximal inhibitory concentration (MIC_50_). Using the plot of the % viability as a function of compound concentration, MIC_50_ values were determined.

### 3.3. Biological activity

Compounds **4a**–**4k** were evaluated for their capacity to inhibit *Mtb*TMPK and growth of an avirulent *M. tuberculosis* strain (Table 1). Most compounds exhibited low micromolar enzyme inhibitory activity and for most analogues the nature and the positions of the D-ring substituents have only negligible effect on the activity. This is in line with the docking scores. However, introduction of two trifluoromethyl groups in meta position of ring D (**4k**) significantly lowered the inhibitory potency. This may be attributed to the fact that the large trifluoromethyl groups hinder the tail of molecule to form a stacking interaction with the enzyme, or the tendency of trifluoromethyl to form a hydrogen bond by directional change of last ring, as suggested by the docking analysis. Unfortunately, only analogue **4g** with a 3,4-dichlorophenyl D-ring showed moderate antimycobacterial activity, while all other compounds were virtually inactive. To further validate the antimycobacterial activity, compounds with good enzyme affinity (**4b**, **4g** and **4i**) were screened on virulent strain (H37Rv). Nevertheless, none of them displayed any whole cell antitubercular activity (MIC > 50 μM).

## 4. Conclusion

Based on previous findings, we report herein the synthesis and biological activities of 1-(piperidin-3-yl)thymine amides as *Mtb*TMPK inhibitors. Modelling was conducted to understand the putative binding mode, resulting in similar docking scores with lead compound **3** for most analogues except compound **4k** bearing sterically demanding substituents on the D-ring. Further *Mtb*TMPK inhibitory activities were in line with the modelling results. Compounds **4b** and **4i** were slightly more potent than the parent compound **3**, while compound **4k** was significantly less active. Unfortunately, most analogues failed to exhibit whole cell antitubercular activity, except for **4g**, which demonstrated moderate cellular activity on avirulent strain. We hypothesise that the poor permeability/uptake of these inhibitors in *M. tuberculosis* may be the reason for the disappointing cellular activity. Attempts to improve mycobacterial uptake will be addressed in future work.

## Supplementary Material

Supplemental Material
