# Supplementary material for: 1-(Piperidin-3-yl)thymine amides as inhibitors of M. tuberculosis thymidylate kinase
Source: J Enzyme Inhib Med Chem. 2019 Dec 11;34(1):1730–9. doi: 10.1080/14756366.2019.1662790 (PMC6920704; doi:10.1080/14756366.2019.1662790)

Support information

1-(Piperidin-3-yl)thymine amides as inhibitors of *M. tuberculosis* thymidylate kinase

Yanlin Jian [a], Martijn D. P. Risseeuw [a], Mathy Froeyen [b], Lijun Song [a], Davie Cappoen [c], Paul Cos [c], Hélène Munier-Lehmann [d] and Serge Van Calenbergh [a, *]

[a] Laboratory for Medicinal Chemistry, Faculty of Pharmaceutical Sciences, Ghent University, Ottergemsesteemweg 460, B-9000 Ghent, Belgium

[b] Department of Medicinal Chemistry, Rega Institute for Medical Research, KU Leuven, Herestraat 49, 3000 Leuven, Belgium

[c] Laboratory for Microbiology, Parasitology and Hygiene (LMPH), Department of Pharmaceutical Sciences, University of Antwerp, Campus Drie Eiken, Universiteitsplein 1, Belgium

[d] Unit of Chemistry and Biocatalysis, Department of Structural Biology and Chemistry, Institut Pasteur, CNRS UMR3523, 28 Rue du Dr. Roux, Cedex 15 75724 Paris, France

Table of contents:

^{1}H-NMR spectrum compound **4a** **S3**
^{13}C-NMR spectrum compound **4a** **S4**
HSQC spectrum compound **4a** **S5**
^{1}H-NMR spectrum compound **4b** **S6**
^{13}C-NMR spectrum compound **4b** **S7**
HSQC spectrum compound **4b** **S8**
^{1}H-NMR spectrum compound **4c** **S9**
^{13}C-NMR spectrum compound **4c** **S9**
^{13}C-NMR spectrum compound **4c** **S10**
HSQC spectrum compound **4c** **S11**
^{1}H-NMR spectrum compound **4d** **S12**
^{13}C-NMR spectrum compound **4d** **S13**
HSQC spectrum compound **4d** **S14**
^{1}H-NMR spectrum compound **4e** **S15**
^{13}C-NMR spectrum compound **4e** **S16**
HSQC spectrum compound **4e** **S17**
^{1}H-NMR spectrum compound **4f** **S18**
^{13}C-NMR spectrum compound **4f** **S19**
HSQC spectrum compound **4f** **S20**
^{1}H-NMR spectrum compound **4g** **S21**
^{13}C-NMR spectrum compound **4g** **S22**
HSQC spectrum compound **4g** **S23**
^{1}H-NMR spectrum compound **4h** **S24**
^{13}C-NMR spectrum compound **4h** **S25**
HSQC spectrum compound **4h** **S26**
^{1}H-NMR spectrum compound **4i** **S27**
^{13}C-NMR spectrum compound **4i** **S28**
HSQC spectrum compound **4i** **S29**
^{1}H-NMR spectrum compound **4j** **S30**
^{13}C-NMR spectrum compound **4j** **S31**
HSQC spectrum compound **4j** **S32**
^{1}H-NMR spectrum compound **4k** **S33**
^{13}C-NMR spectrum compound **4k** **S34**
HSQC spectrum compound **4k** **S35**

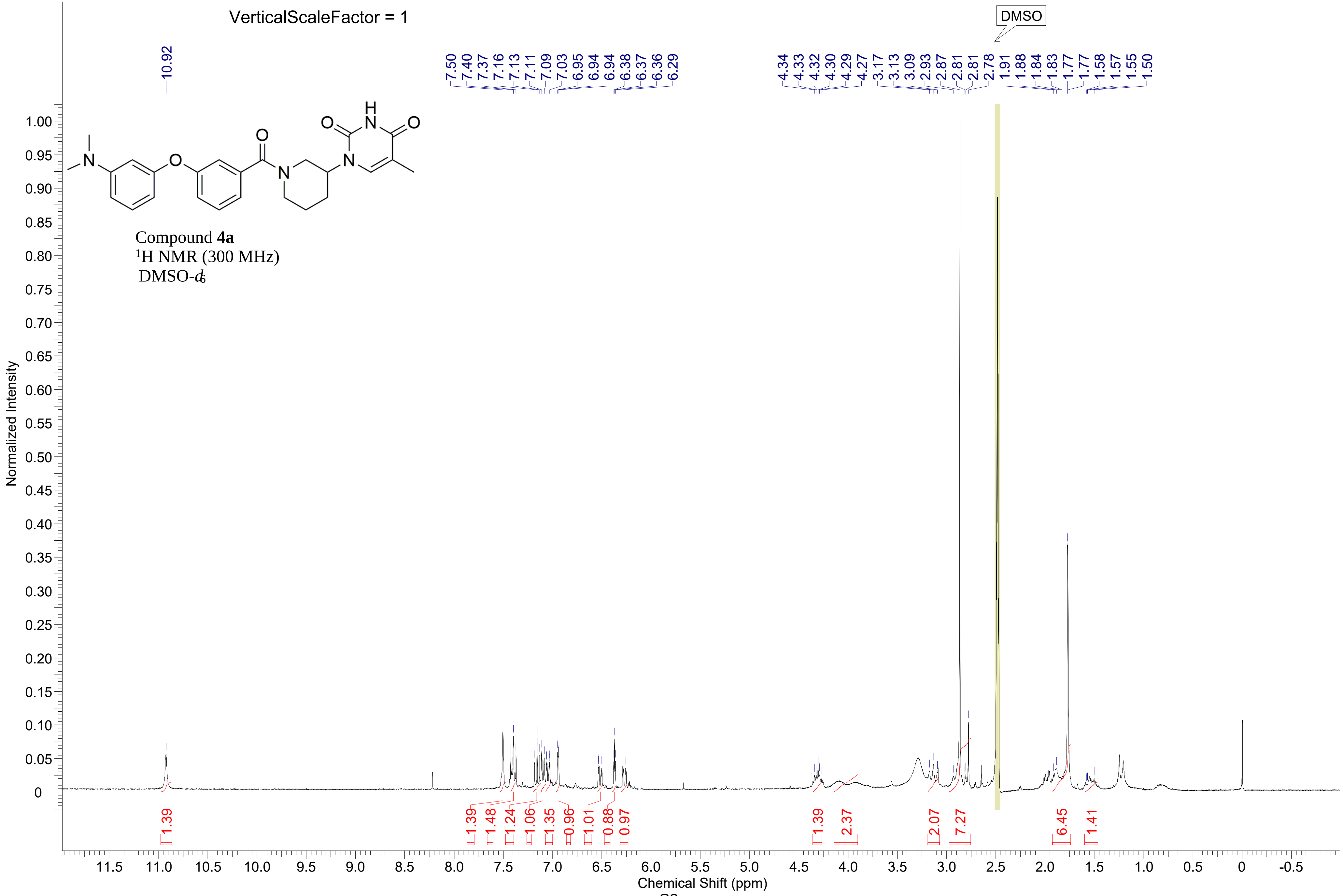
VerticalScaleFactor = 1
DMSO
10.92
7.50 7.40 7.37 7.16 7.13 7.11 7.09 7.03 6.95 6.94 6.94 6.38 6.37 6.36 6.29
4.34 4.33 4.32 4.30 4.29 4.27 3.17 3.13 3.09 2.93 2.87 2.81 2.81 2.78 1.91 1.88 1.84 1.83 1.77 1.77 1.58 1.57 1.55 1.50
Compound 4a
1H NMR (300 MHz)
DMSO-d6
Normalized Intensity
1.39
1.39 1.48 1.24 1.06 1.35 0.96 1.01 0.88 0.97
1.39 2.37 2.07 7.27 6.45 1.41
Chemical Shift (ppm)

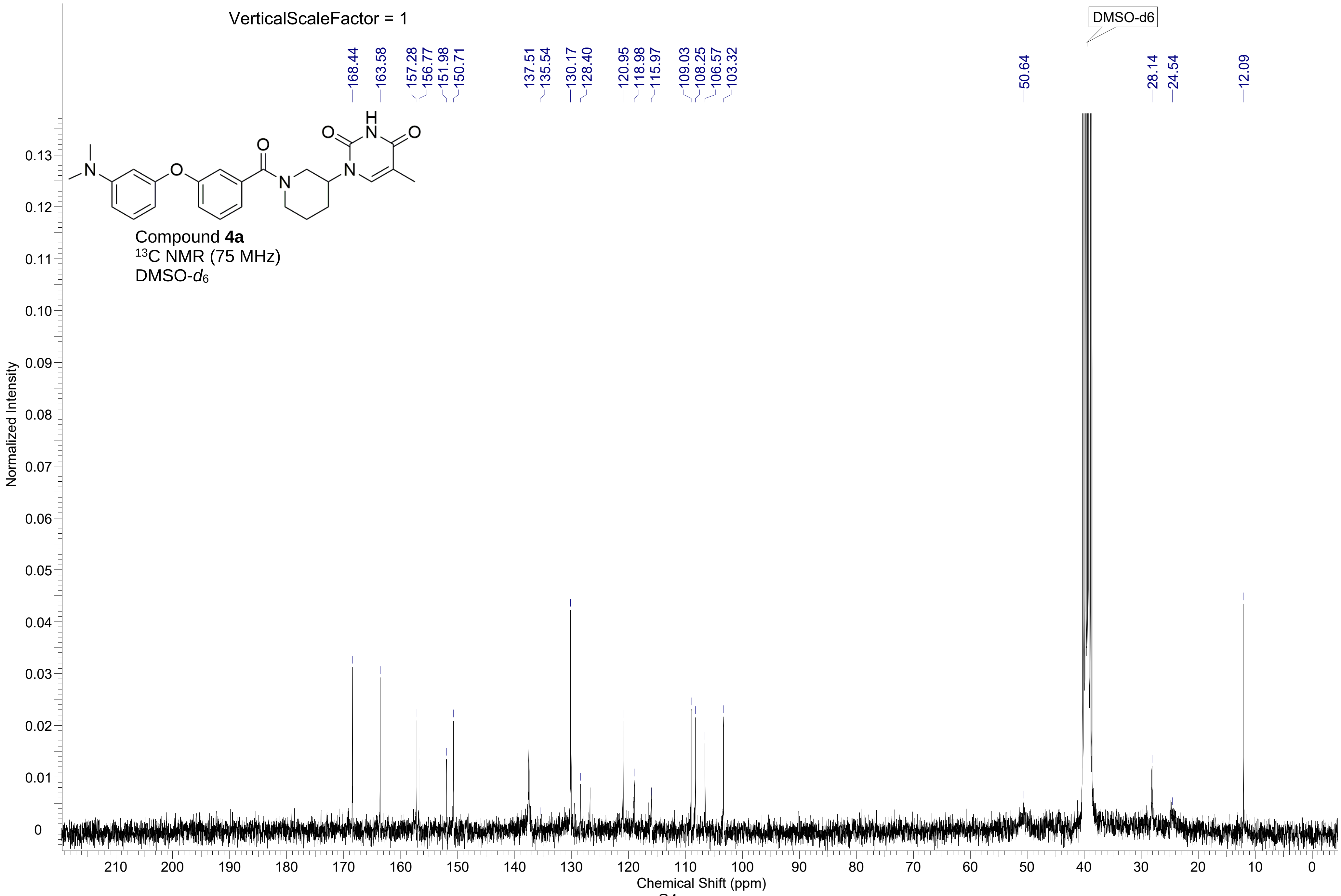
VerticalScaleFactor = 1
DMSO-d6
168.44
163.58
157.28
156.77
151.98
150.71
137.51
135.54
130.17
128.40
120.95
118.98
115.97
109.03
108.25
106.57
103.32
50.64
28.14
24.54
12.09
Compound **4a**
^{13}C NMR (75 MHz)
DMSO-d_6
Normalized Intensity
Chemical Shift (ppm)

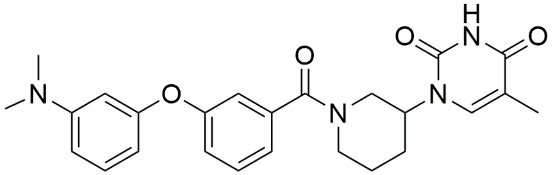

Compound **4a**
HSQC
DMSO-d_6

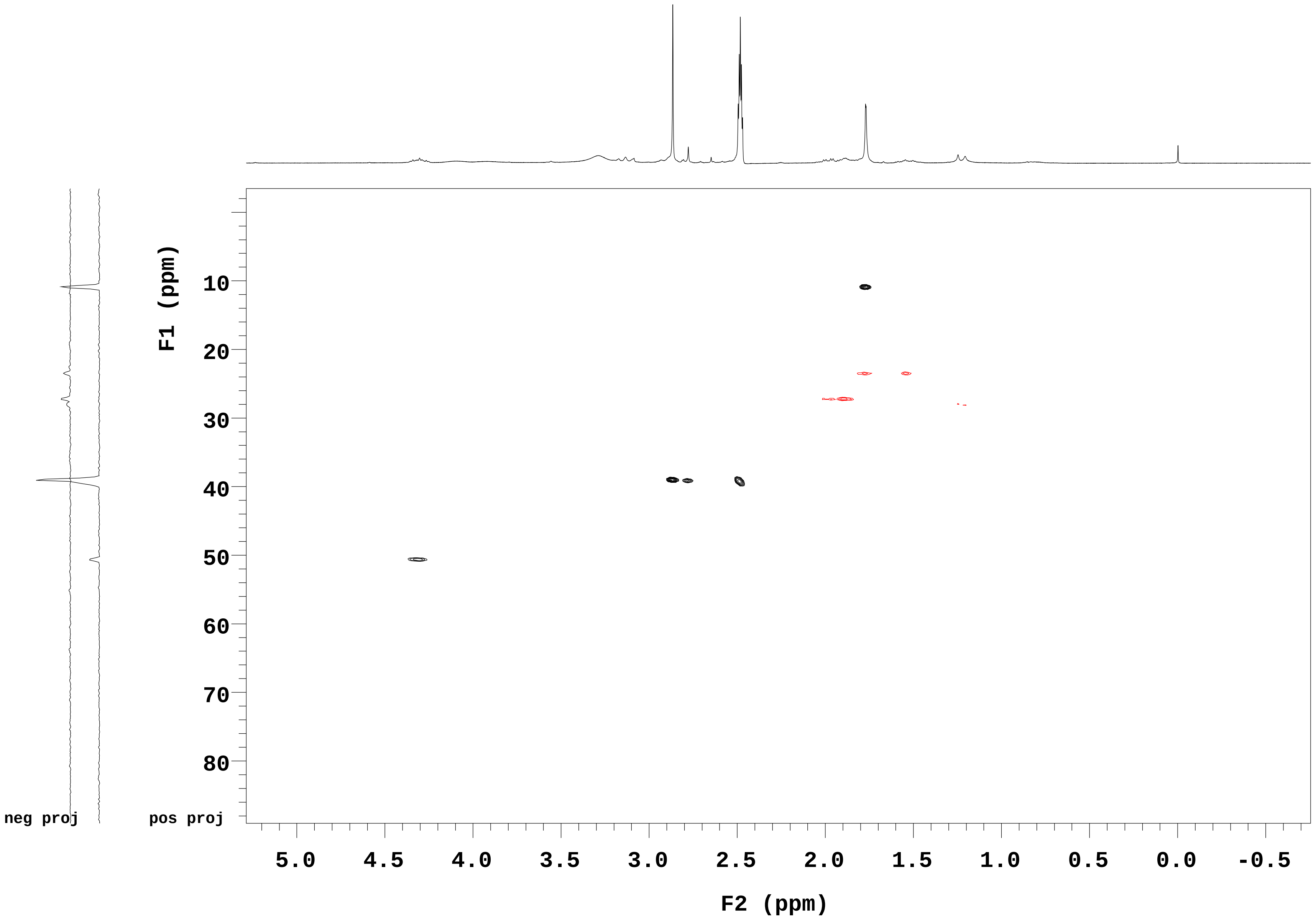

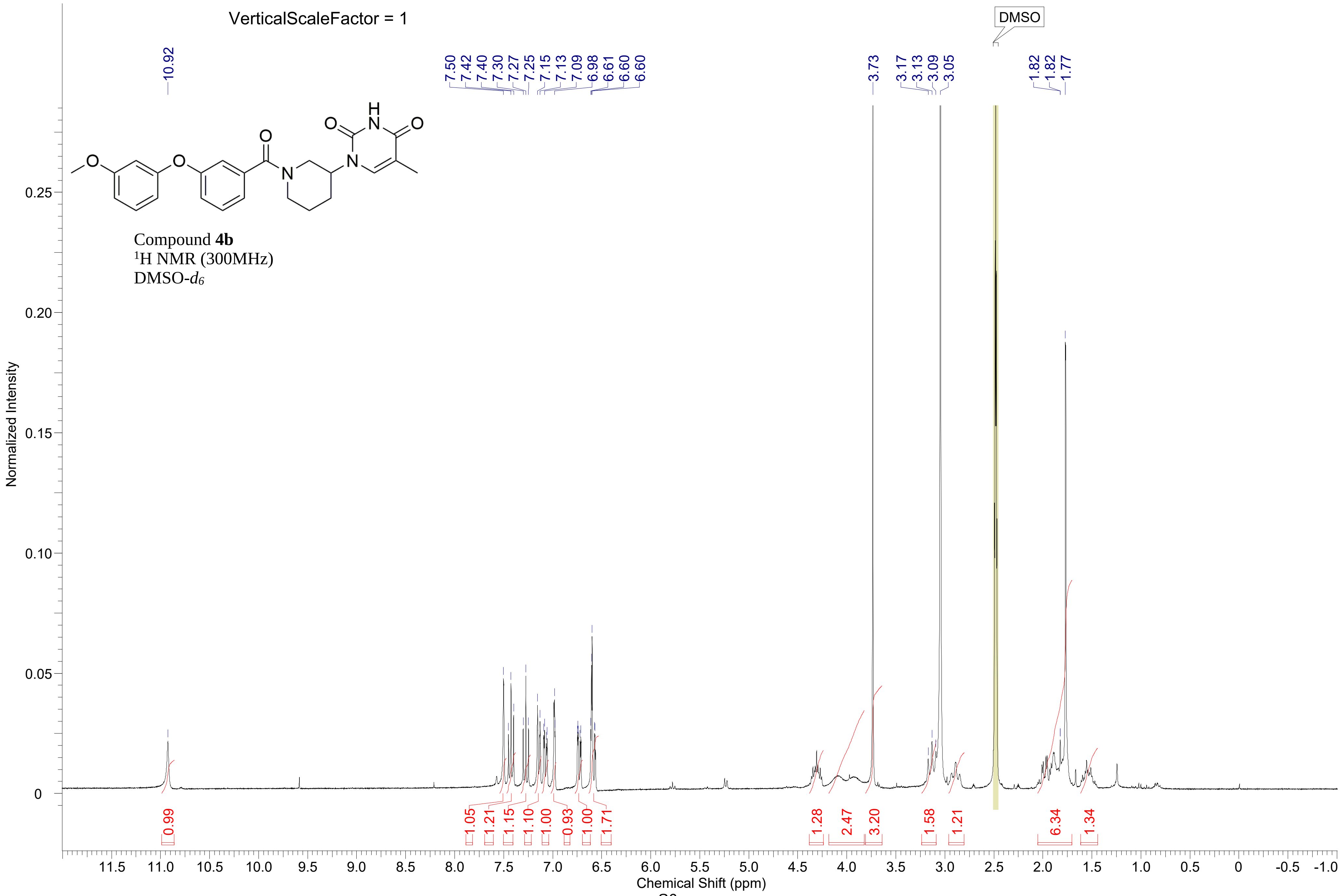
VerticalScaleFactor = 1
DMSO
Compound 4b
¹H NMR (300MHz)
DMSO-d6
10.92
7.50 7.42 7.40 7.30 7.27 7.25 7.15 7.13 7.09 6.98 6.61 6.60 6.60
3.73
3.17 3.13 3.09 3.05
1.82 1.82 1.77
0.99 1.05 1.21 1.15 1.10 1.00 0.93 1.00 1.71 1.28 2.47 3.20 1.58 1.21 6.34 1.34
Normalized Intensity
Chemical Shift (ppm)

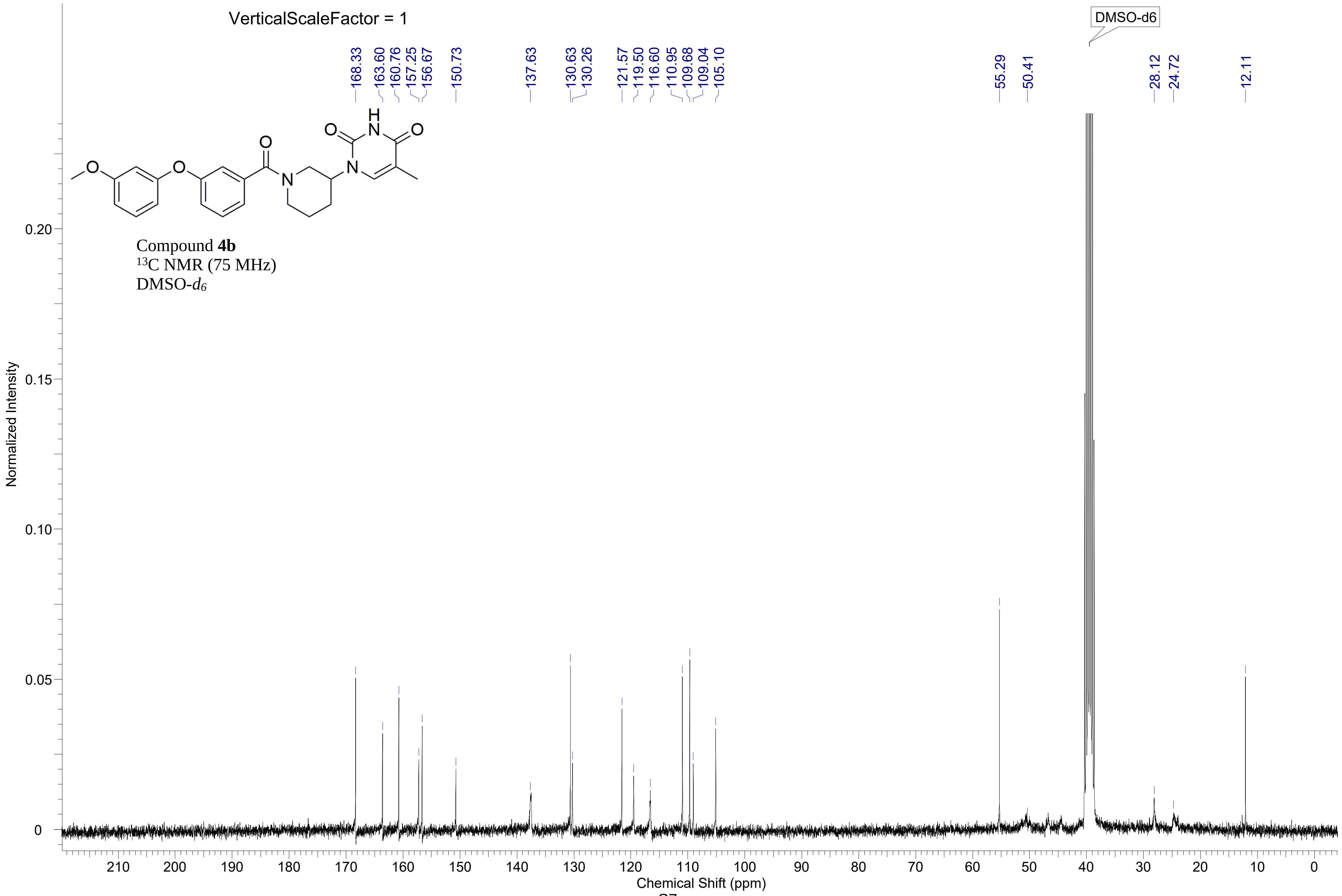
VerticalScaleFactor = 1
DMSO-d6
168.33
163.60
160.76
157.25
156.67
150.73
137.63
130.63
130.26
121.57
119.50
116.60
110.95
109.68
109.04
105.10
55.29
50.41
28.12
24.72
12.11
Compound 4b
13C NMR (75 MHz)
DMSO-d6
Normalized Intensity
Chemical Shift (ppm)

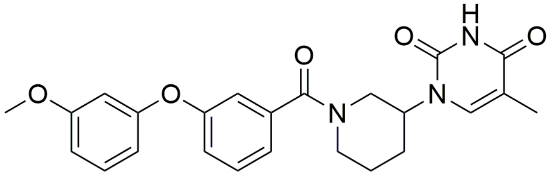

Compound **4b**
HSQC
DMSO-*d*$_6$

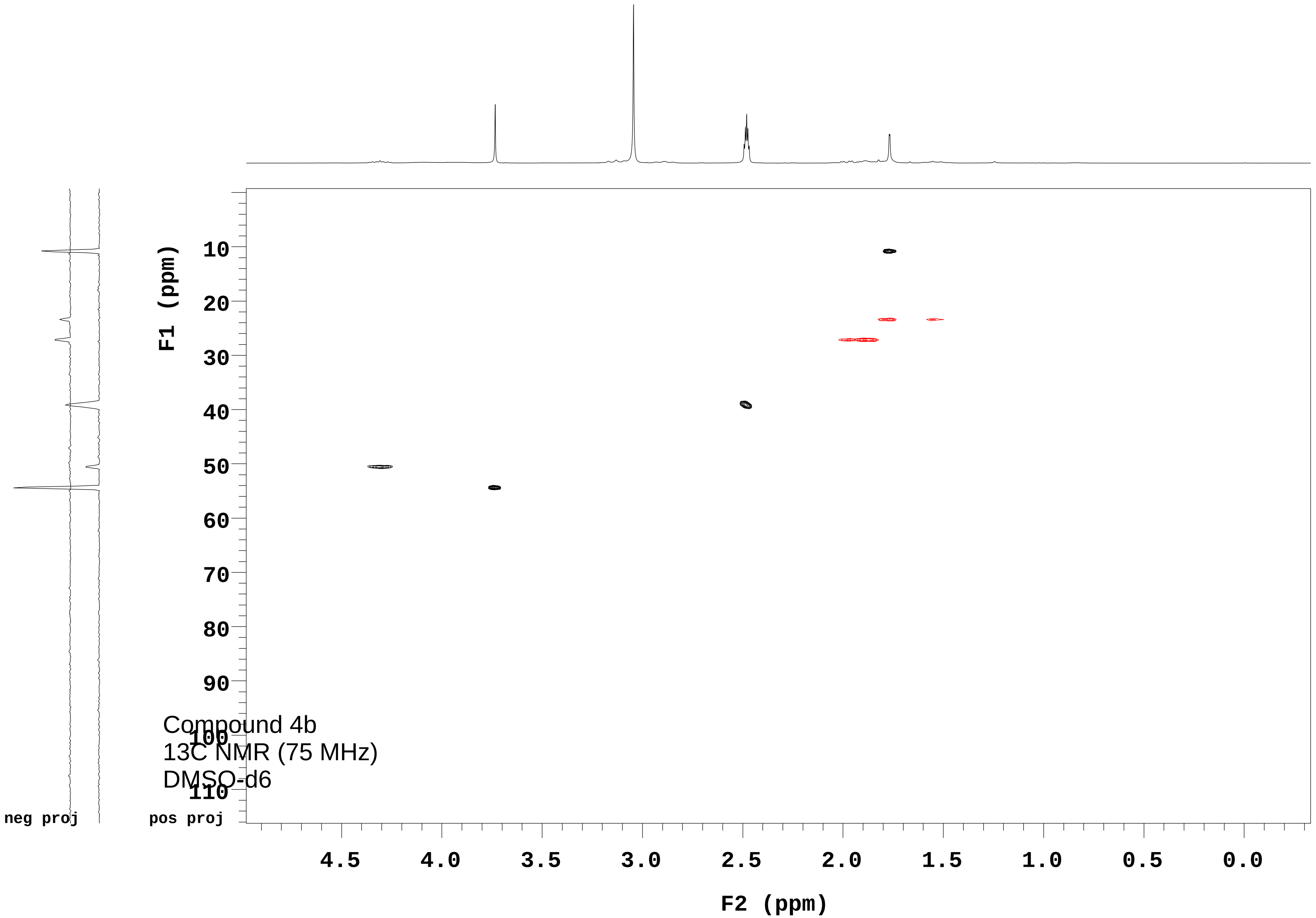

Plotname: YS1045_80TEMP_gHSQC_10Apr2017_01_plot01

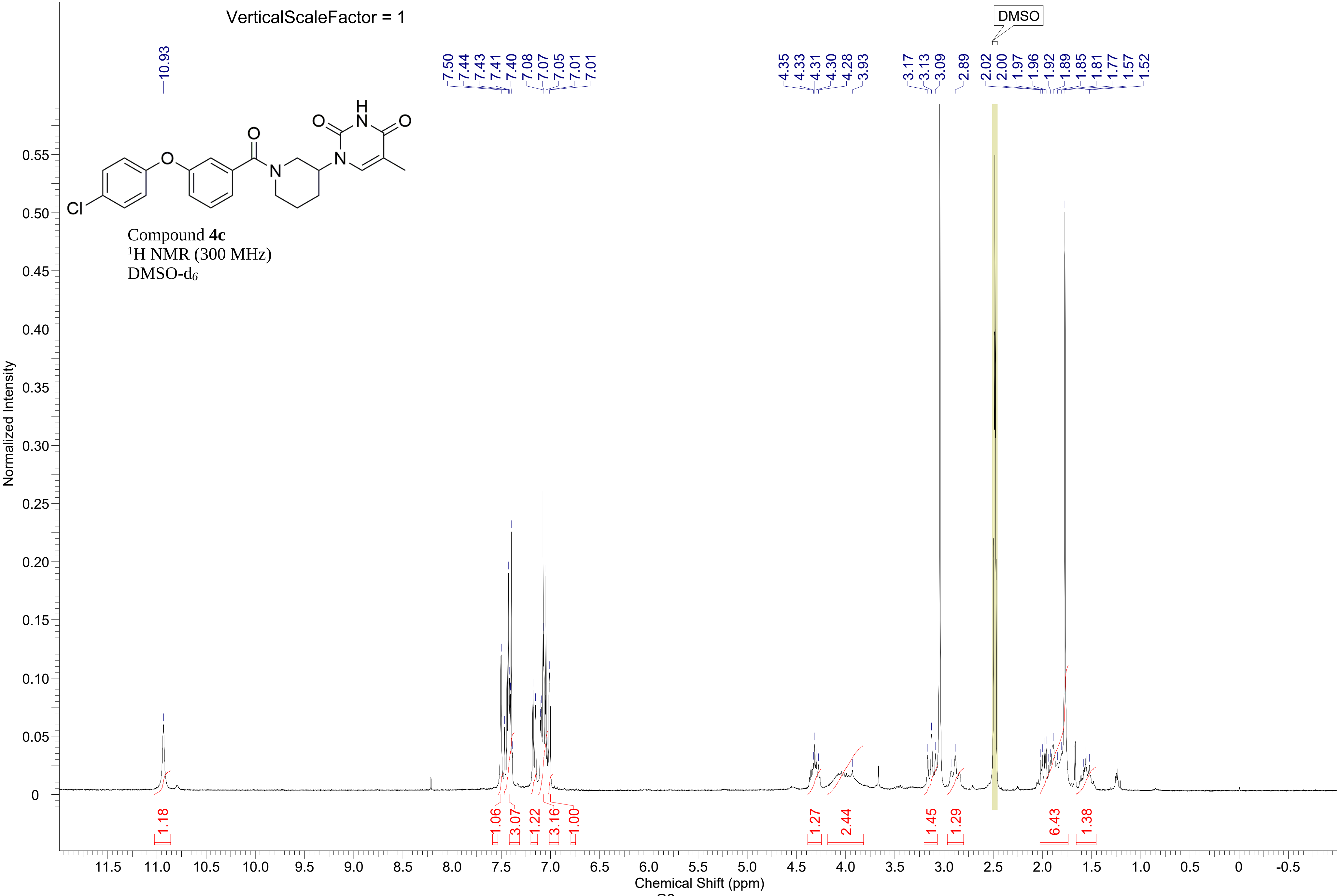
VerticalScaleFactor = 1
DMSO
10.93
7.50 7.44 7.43 7.41 7.40 7.08 7.07 7.05 7.01 7.01
4.35 4.33 4.31 4.30 4.28 3.93
3.17 3.13 3.09 2.89
2.02 2.00 1.97 1.96 1.92 1.89 1.85 1.81 1.77 1.57 1.52
Compound 4c
1H NMR (300 MHz)
DMSO-d6
Cl
O
N
H
1.18
1.06 3.07 1.22 3.16 1.00
1.27 2.44
1.45 1.29
6.43 1.38
Normalized Intensity
Chemical Shift (ppm)

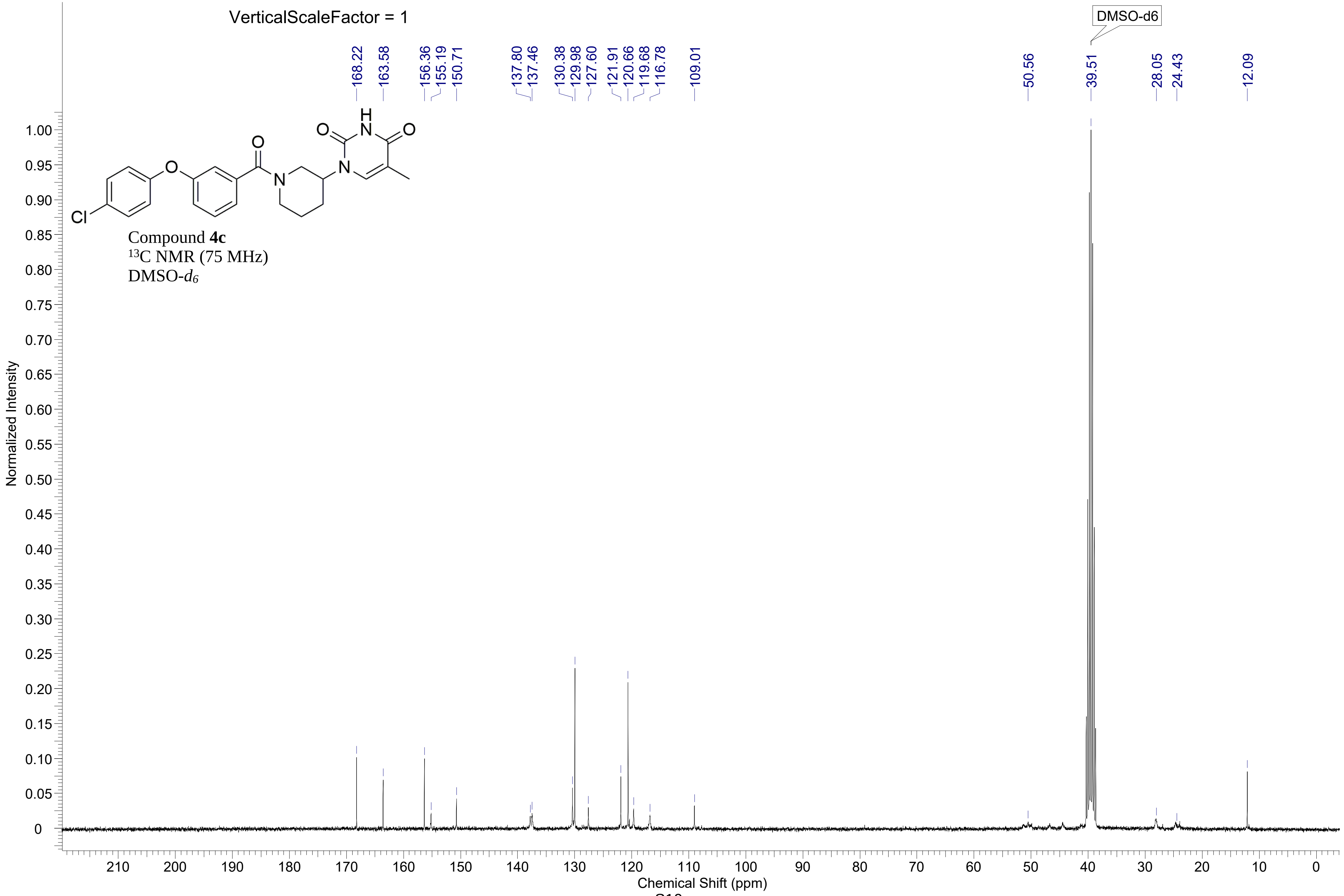
VerticalScaleFactor = 1
DMSO-d6
168.22
163.58
156.36
155.19
150.71
137.80
137.46
130.38
129.98
127.60
121.91
120.66
119.68
116.78
109.01
50.56
39.51
28.05
24.43
12.09
Compound **4c**
^{13}C NMR (75 MHz)
DMSO-d_6
Normalized Intensity
Chemical Shift (ppm)

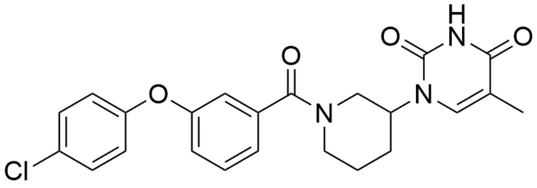

Compound **4c**
HSQC
DMSO-d_6

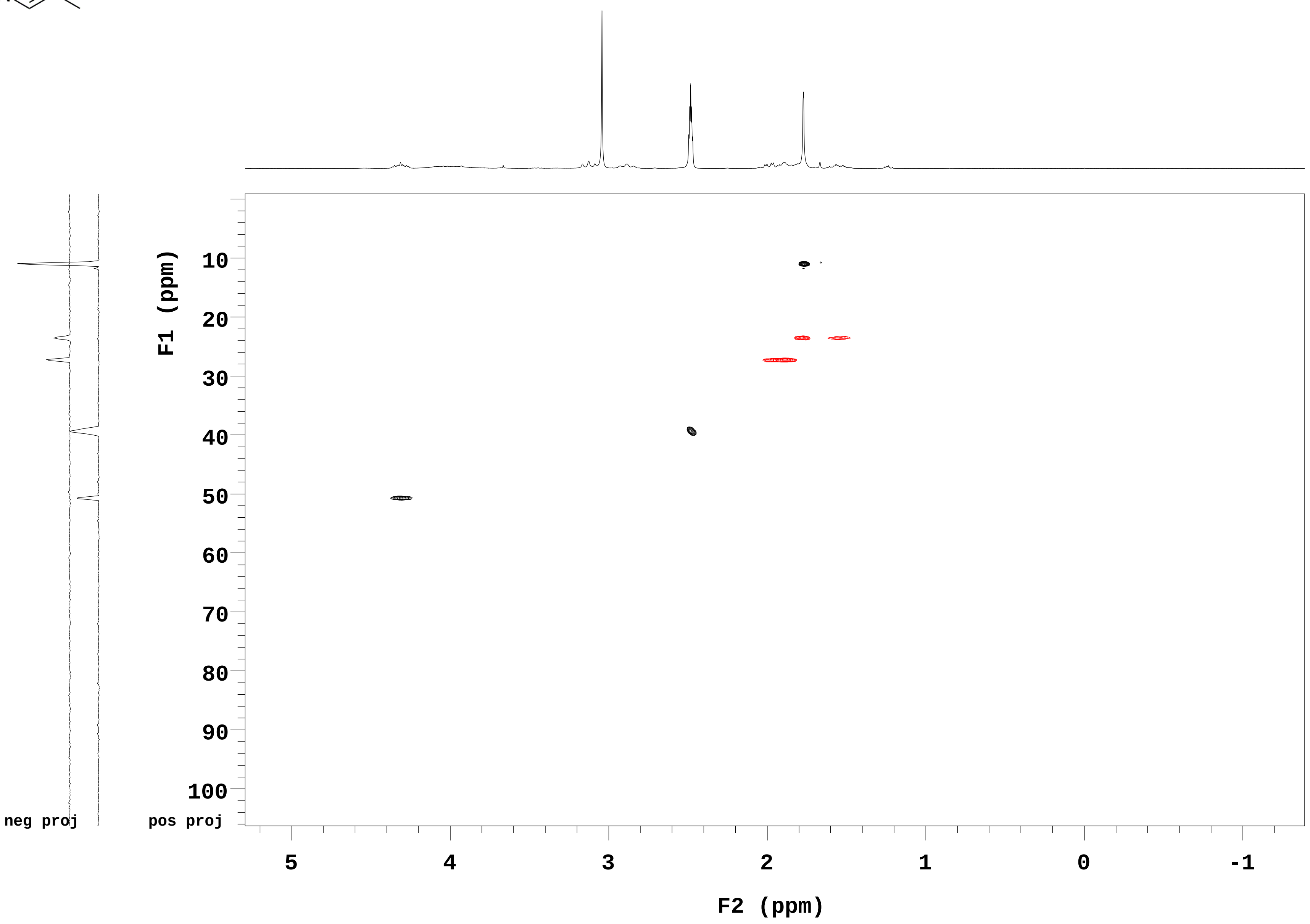

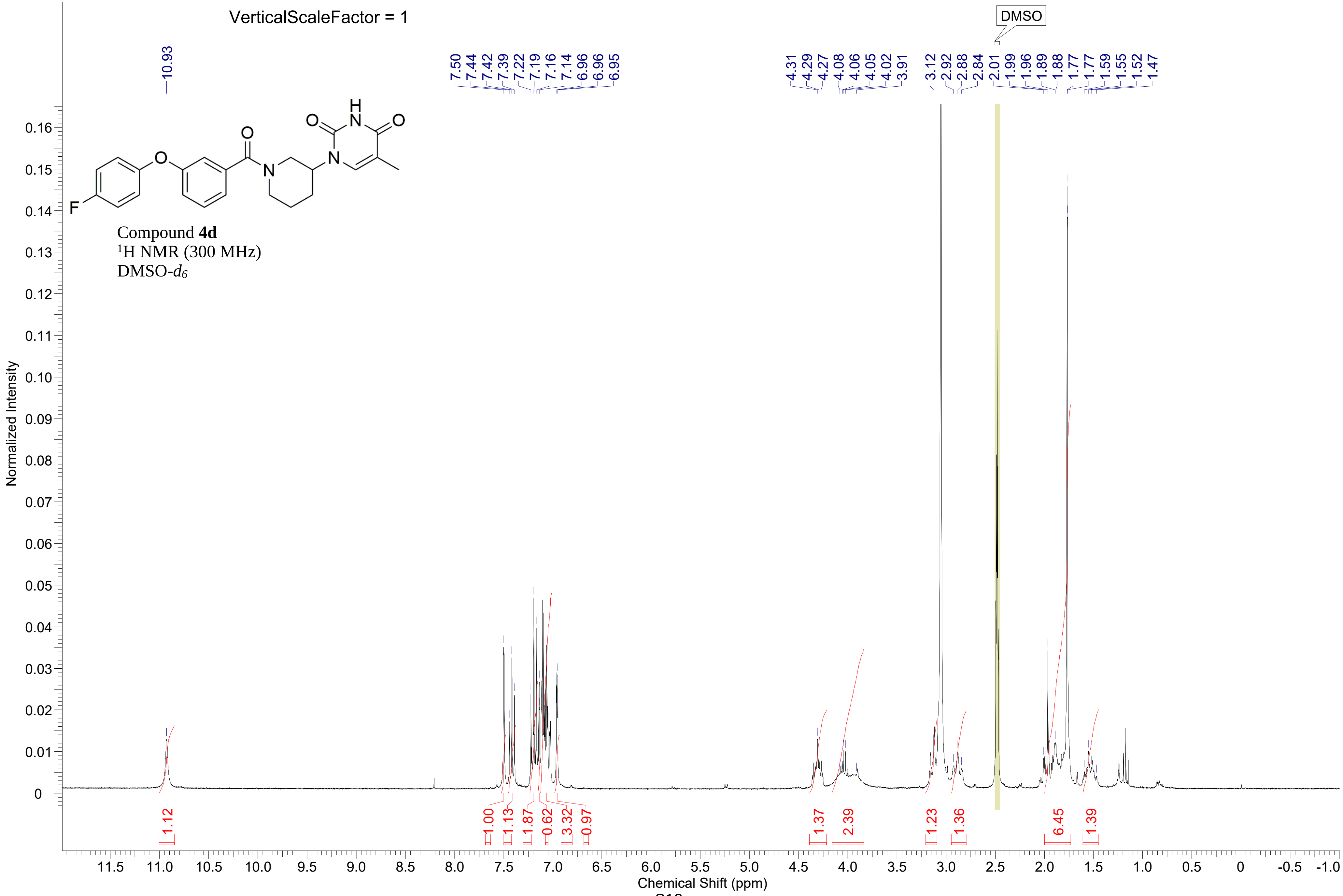

VerticalScaleFactor = 1
DMSO
Compound 4d
1H NMR (300 MHz)
DMSO-d6
10.93
7.50 7.44 7.42 7.39 7.22 7.19 7.16 7.14 6.96 6.96 6.95
4.31 4.29 4.27 4.08 4.06 4.05 4.02 3.91
3.12 2.92 2.88 2.84
2.01 1.99 1.96 1.89 1.88 1.77 1.77 1.59 1.55 1.52 1.47
1.12 1.00 1.13 1.87 0.62 3.32 0.97 1.37 2.39 1.23 1.36 6.45 1.39
Normalized Intensity
Chemical Shift (ppm)

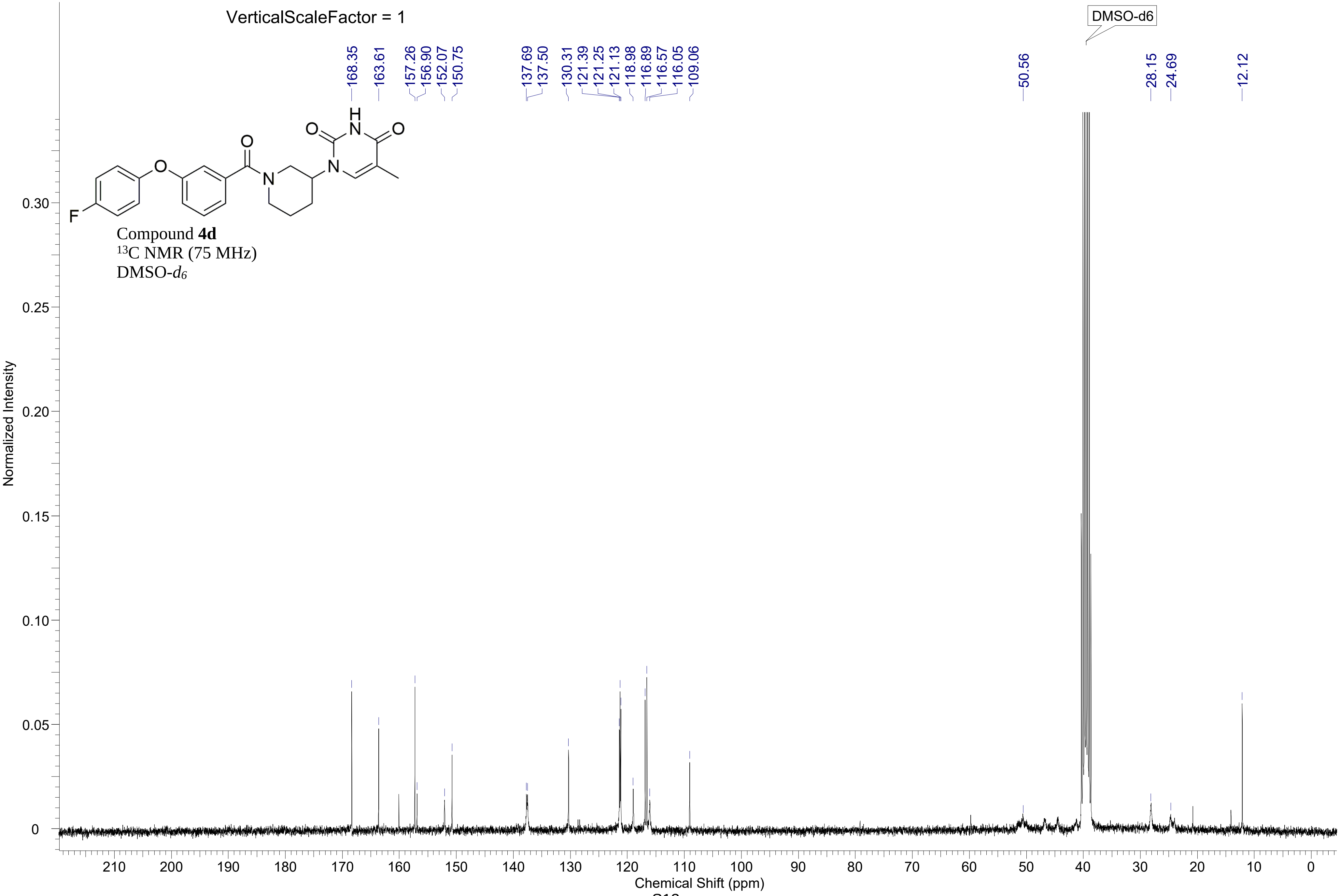

VerticalScaleFactor = 1
DMSO-d6
168.35
163.61
157.26
156.90
152.07
150.75
137.69
137.50
130.31
121.39
121.25
121.13
118.98
116.89
116.57
116.05
109.06
50.56
28.15
24.69
12.12
Compound 4d
13C NMR (75 MHz)
DMSO-d6
Normalized Intensity
Chemical Shift (ppm)

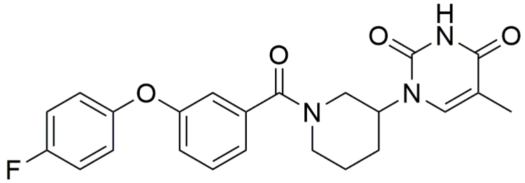

Compound **4d**
HSQC
DMSO-d_6

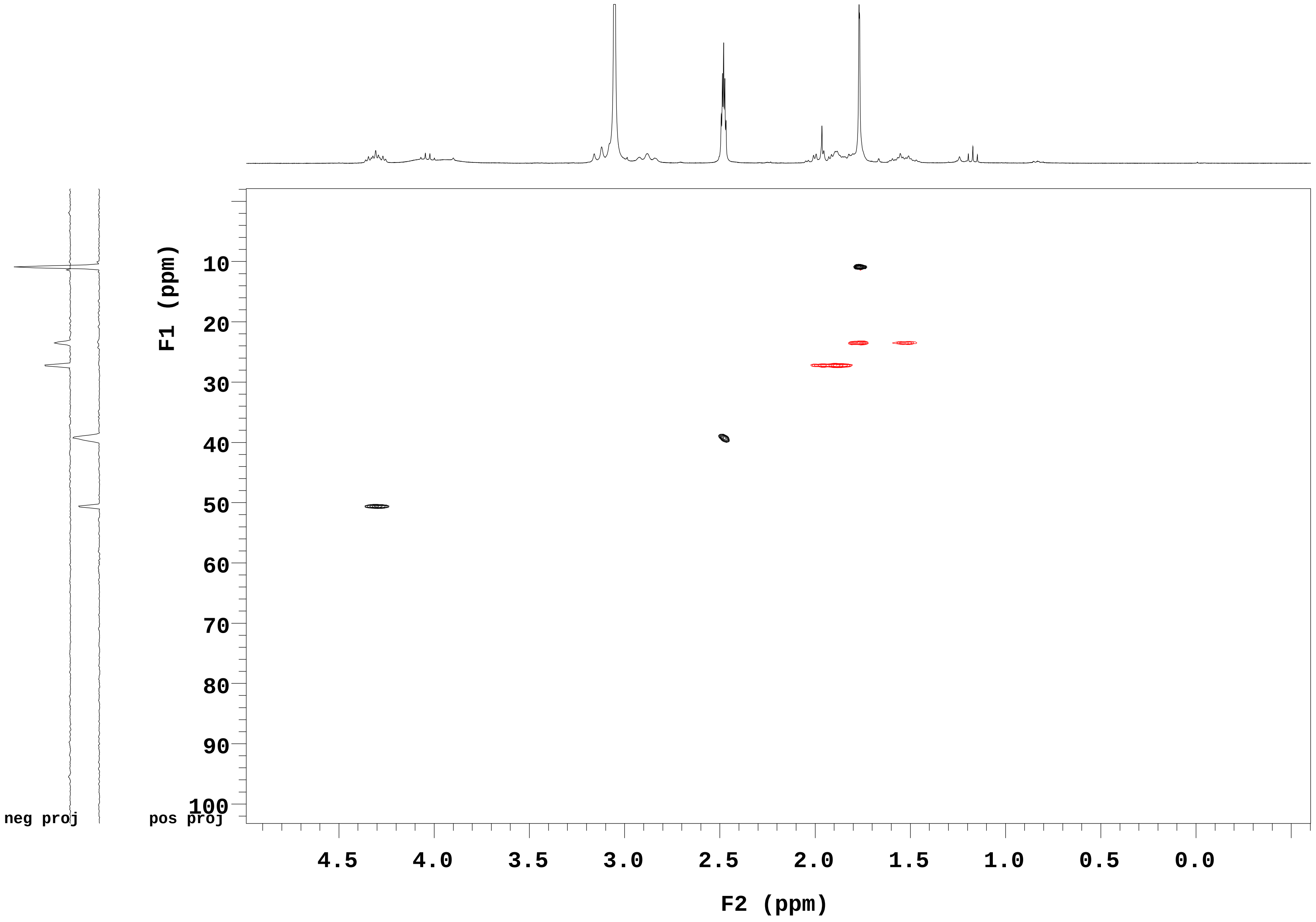

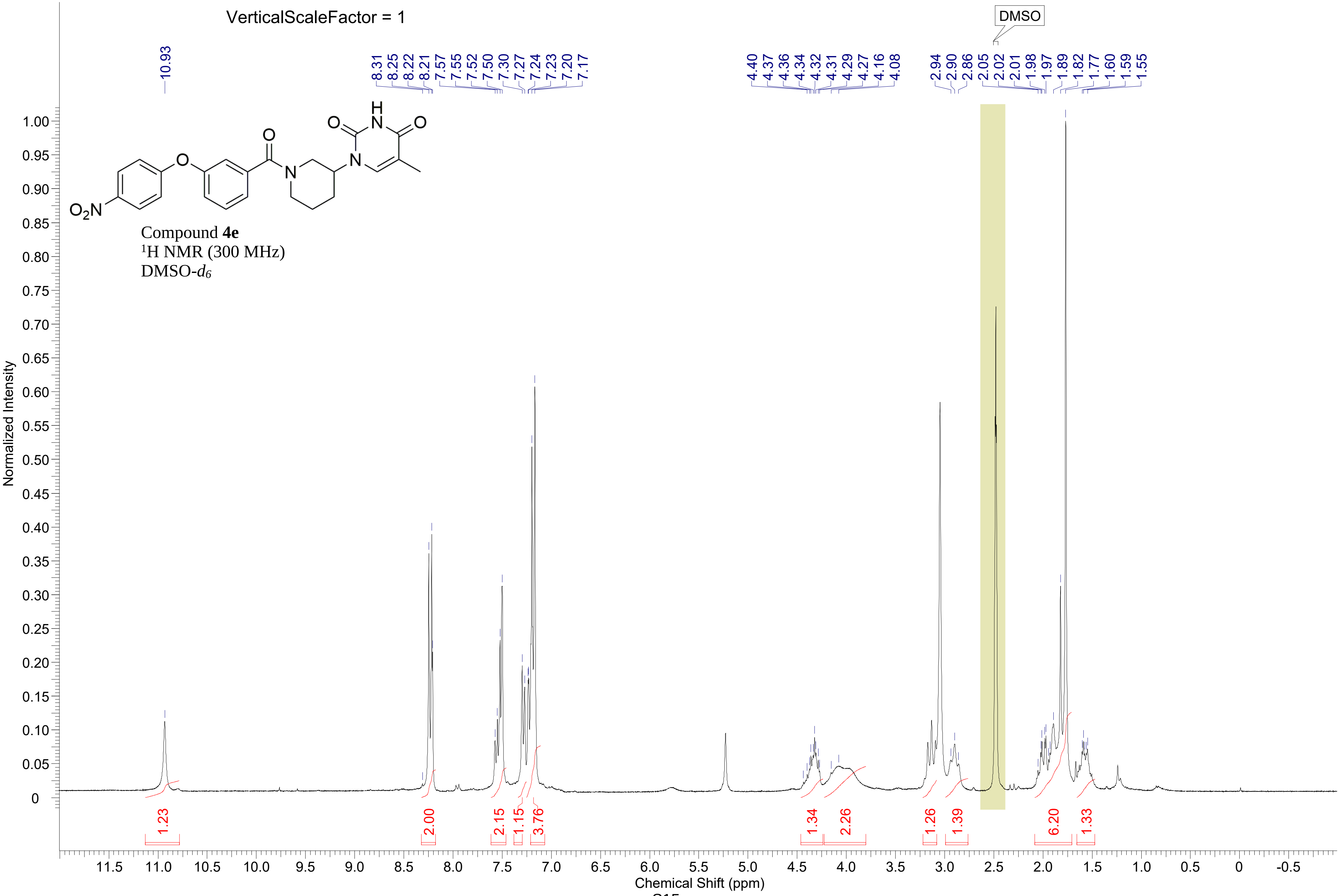
VerticalScaleFactor = 1
DMSO
Compound 4e
1H NMR (300 MHz)
DMSO-d6
Normalized Intensity
Chemical Shift (ppm)

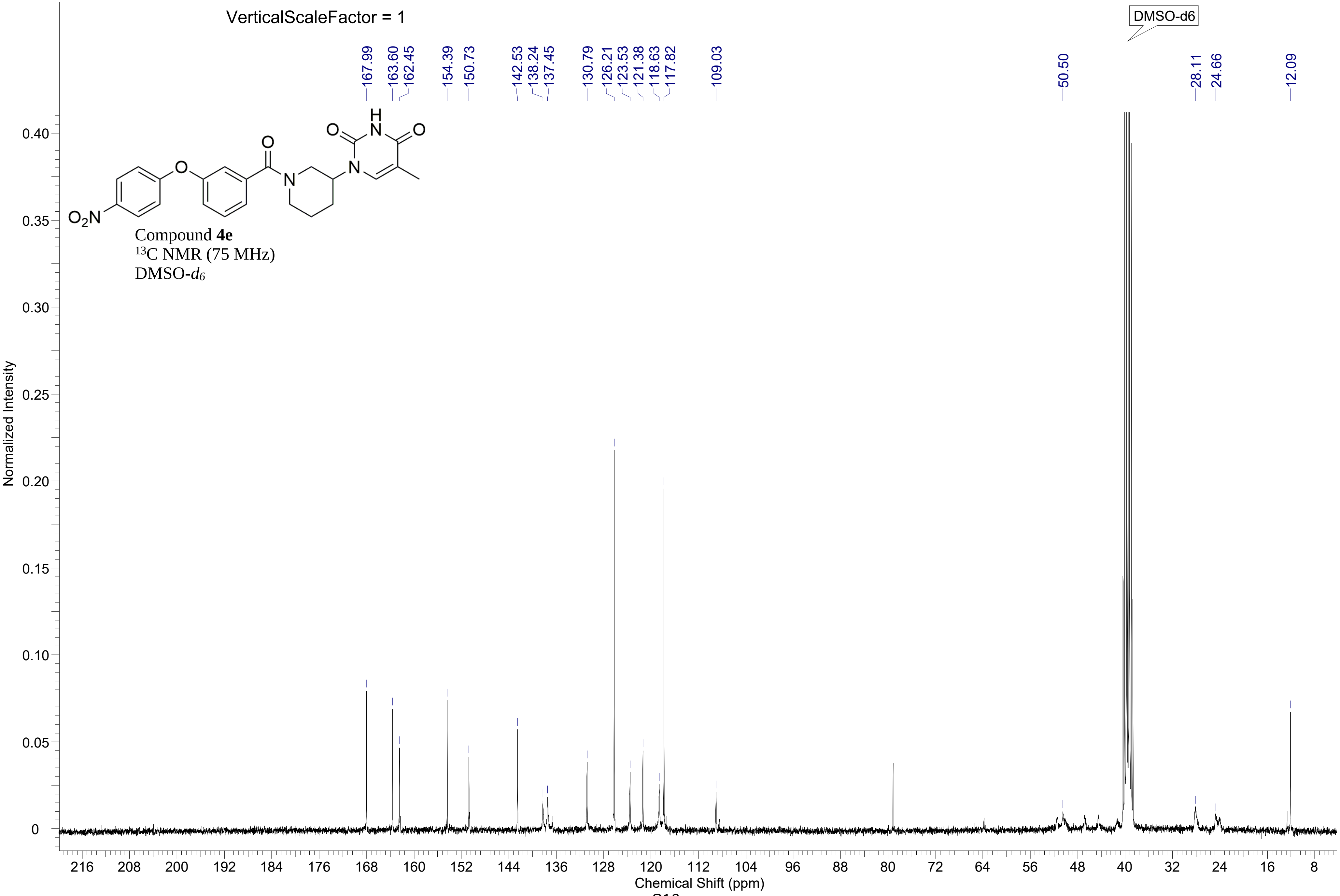
VerticalScaleFactor = 1
DMSO-d6
167.99
163.60
162.45
154.39
150.73
142.53
138.24
137.45
130.79
126.21
123.53
121.38
118.63
117.82
109.03
50.50
28.11
24.66
12.09
Compound 4e
13C NMR (75 MHz)
DMSO-d6
Normalized Intensity
Chemical Shift (ppm)

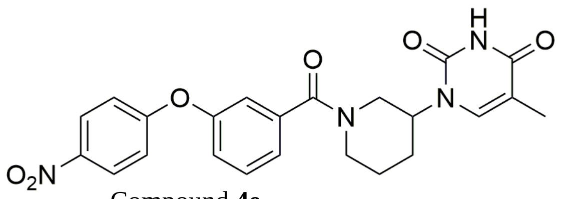

Compound **4e**
HSQC
DMSO-d_6

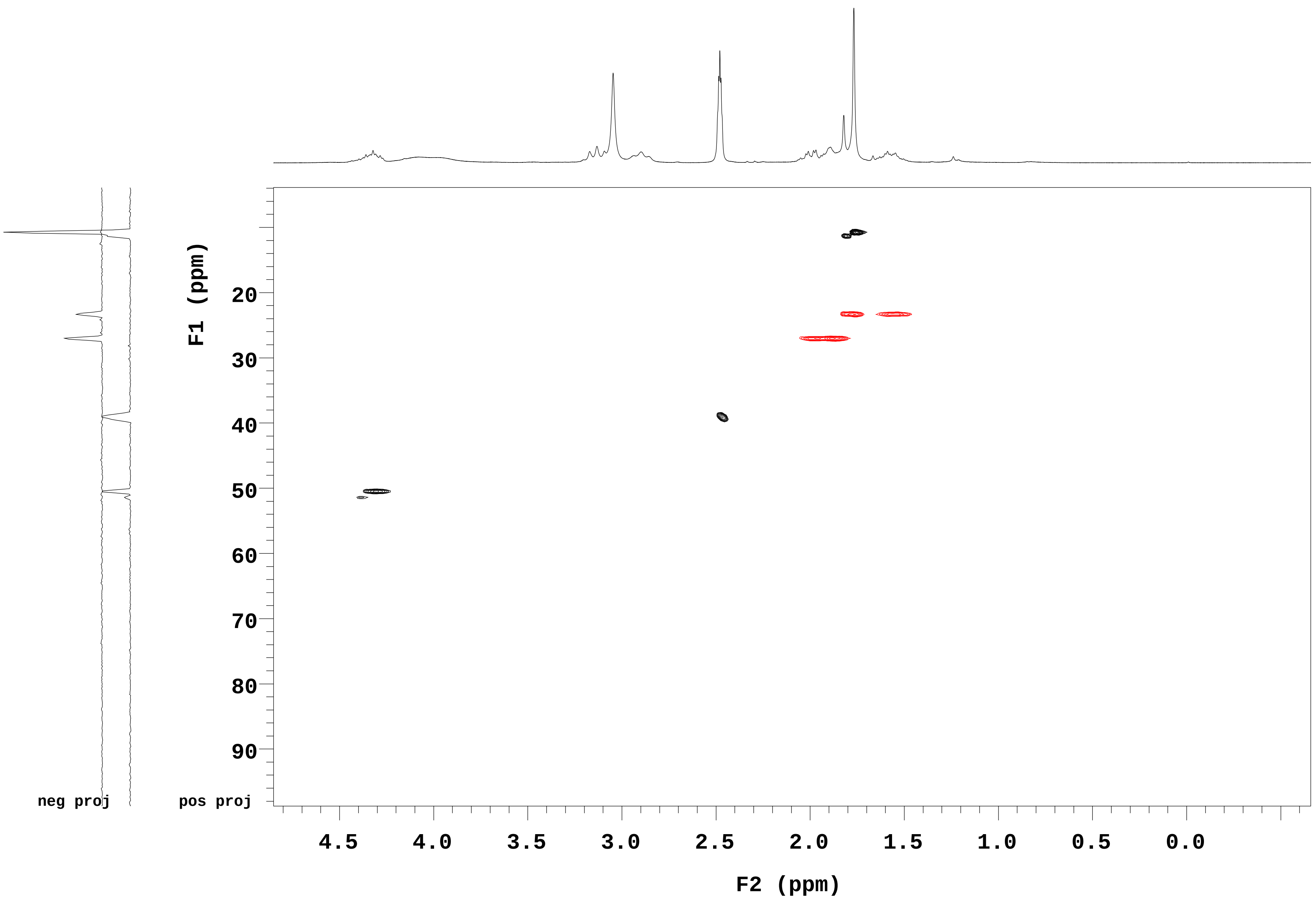

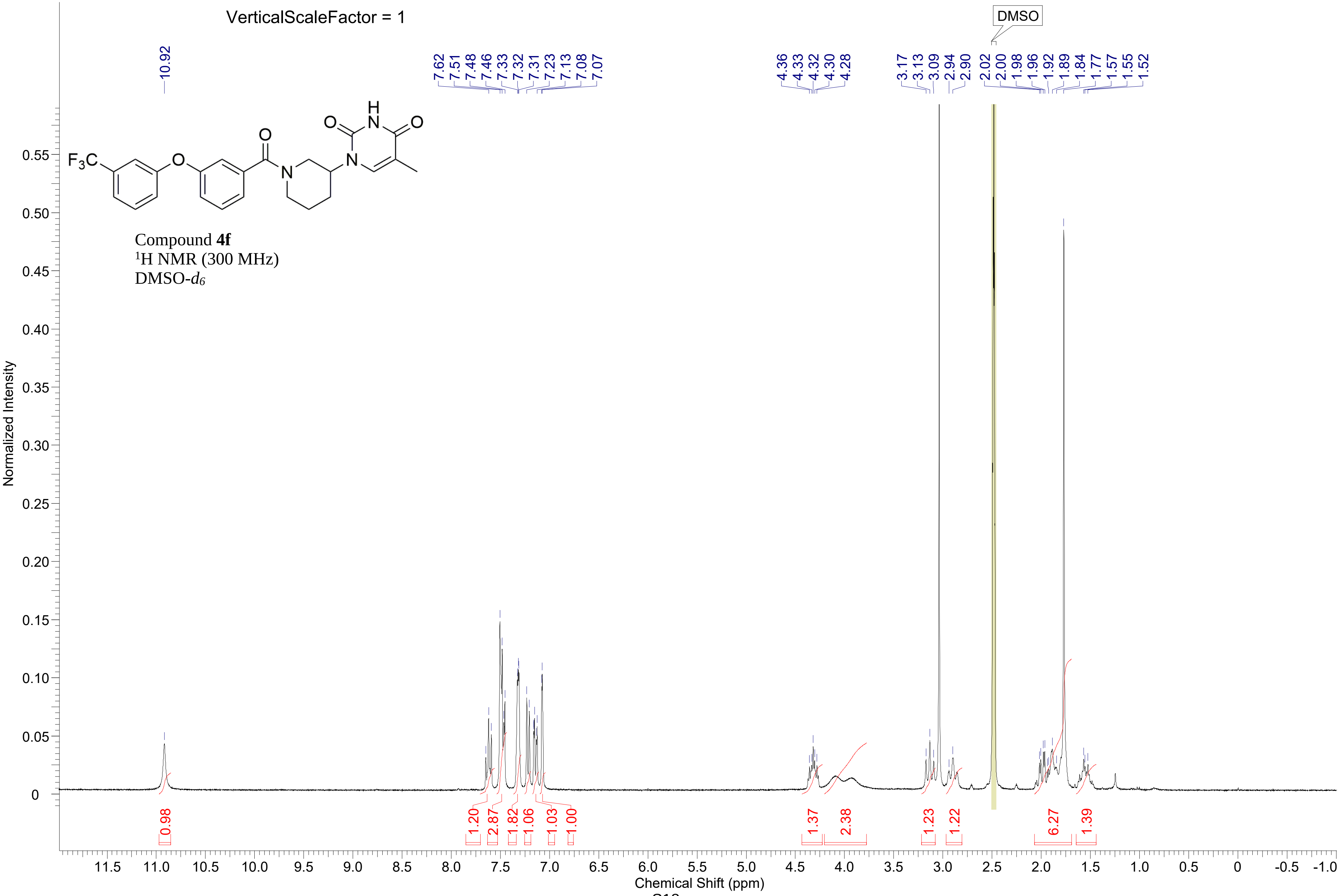
VerticalScaleFactor = 1
DMSO
10.92
7.62 7.51 7.48 7.46 7.33 7.32 7.31 7.23 7.13 7.08 7.07
4.36 4.33 4.32 4.30 4.28
3.17 3.13 3.09 2.94 2.90
2.02 2.00 1.98 1.96 1.92 1.89 1.84 1.77 1.57 1.55 1.52
Compound 4f
1H NMR (300 MHz)
DMSO-d6
Normalized Intensity
0.98
1.20 2.87 1.82 1.06 1.03 1.00
1.37 2.38
1.23 1.22
6.27 1.39
Chemical Shift (ppm)

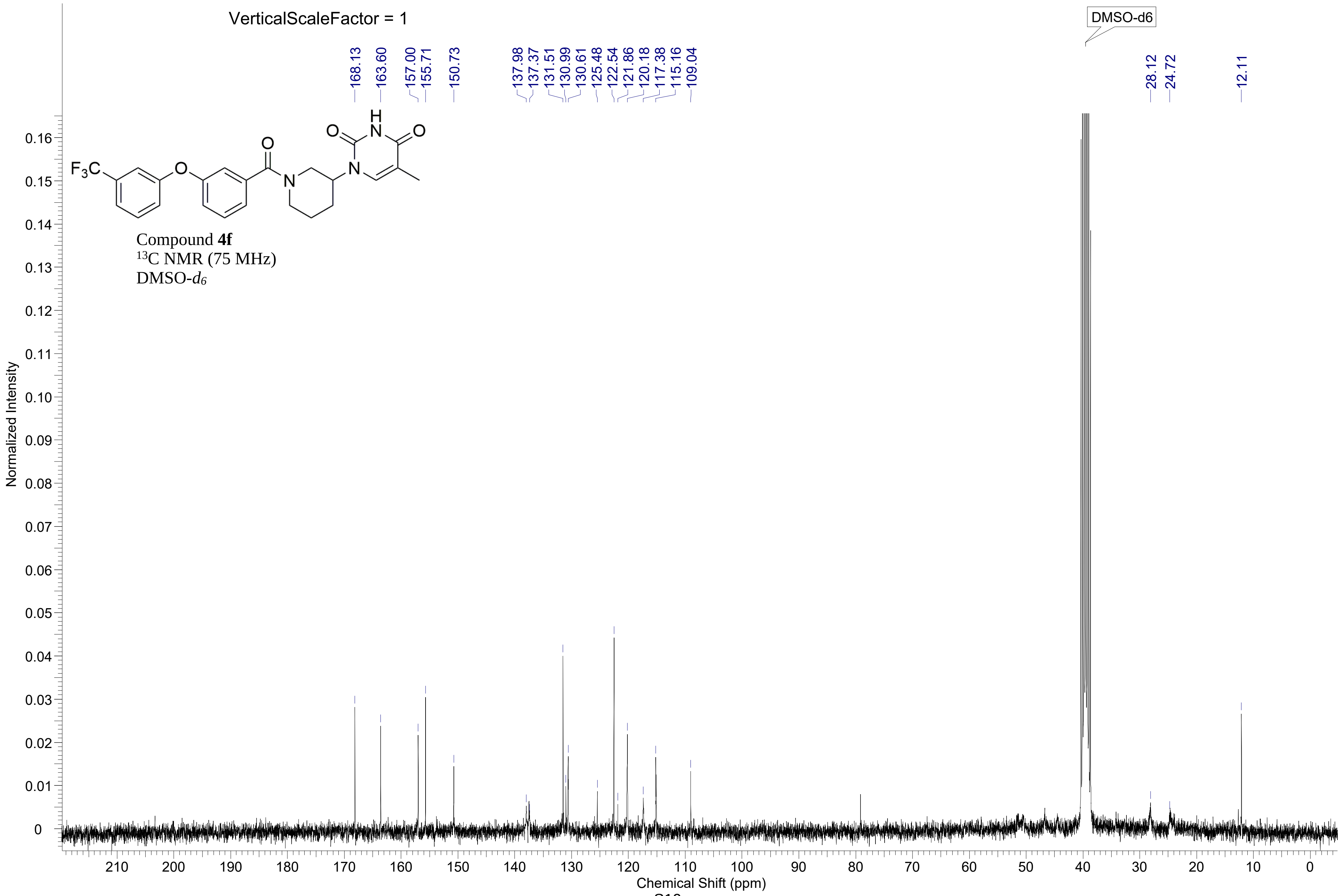
VerticalScaleFactor = 1
DMSO-d6
168.13
163.60
157.00
155.71
150.73
137.98
137.37
131.51
130.99
130.61
125.48
122.54
121.86
120.18
117.38
115.16
109.04
28.12
24.72
12.11
F3C
O
N
H
Compound 4f
13C NMR (75 MHz)
DMSO-d6
Normalized Intensity
Chemical Shift (ppm)

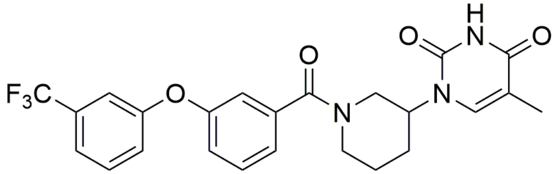

Compound **4f**
HSQC
DMSO-d_6

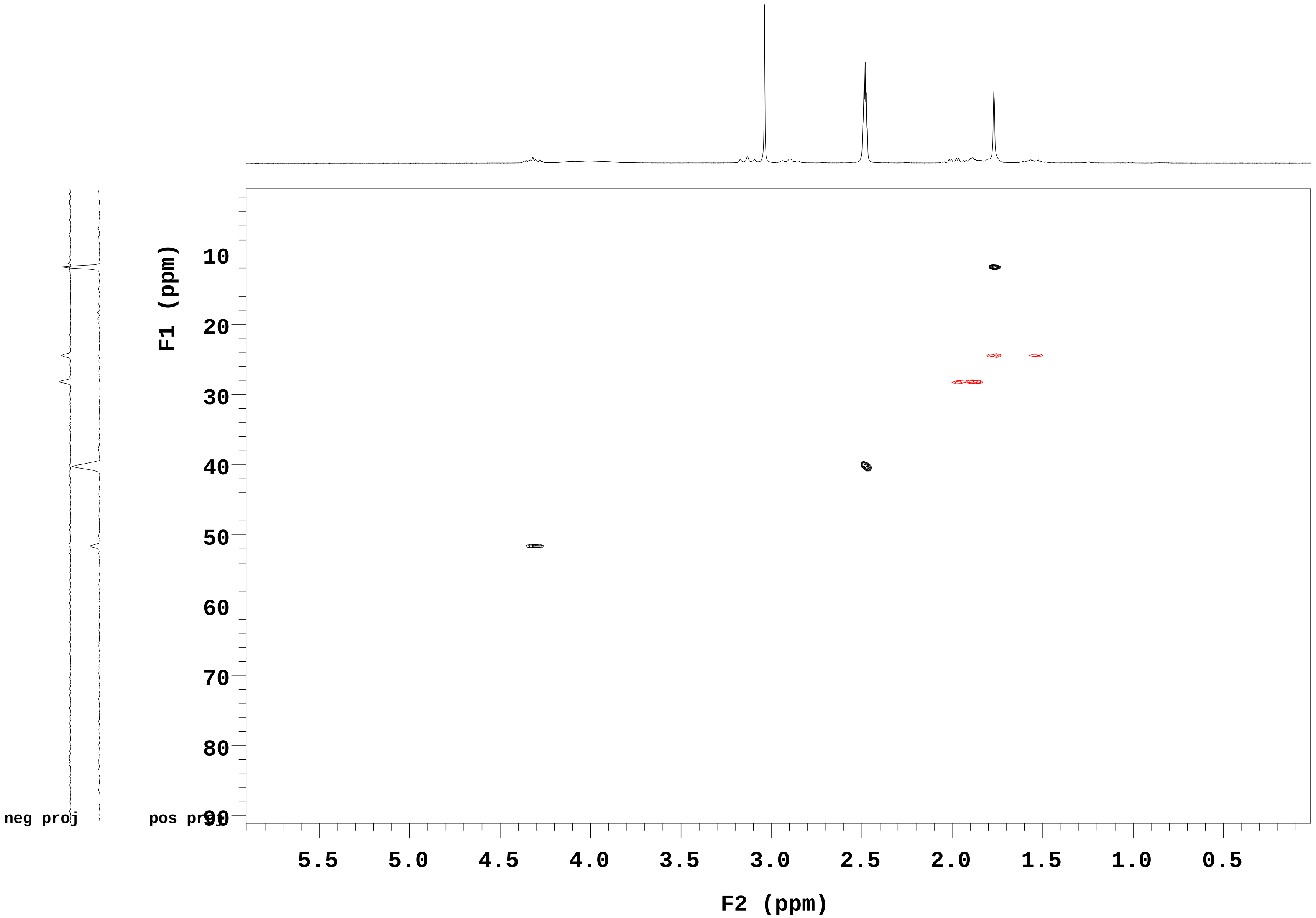

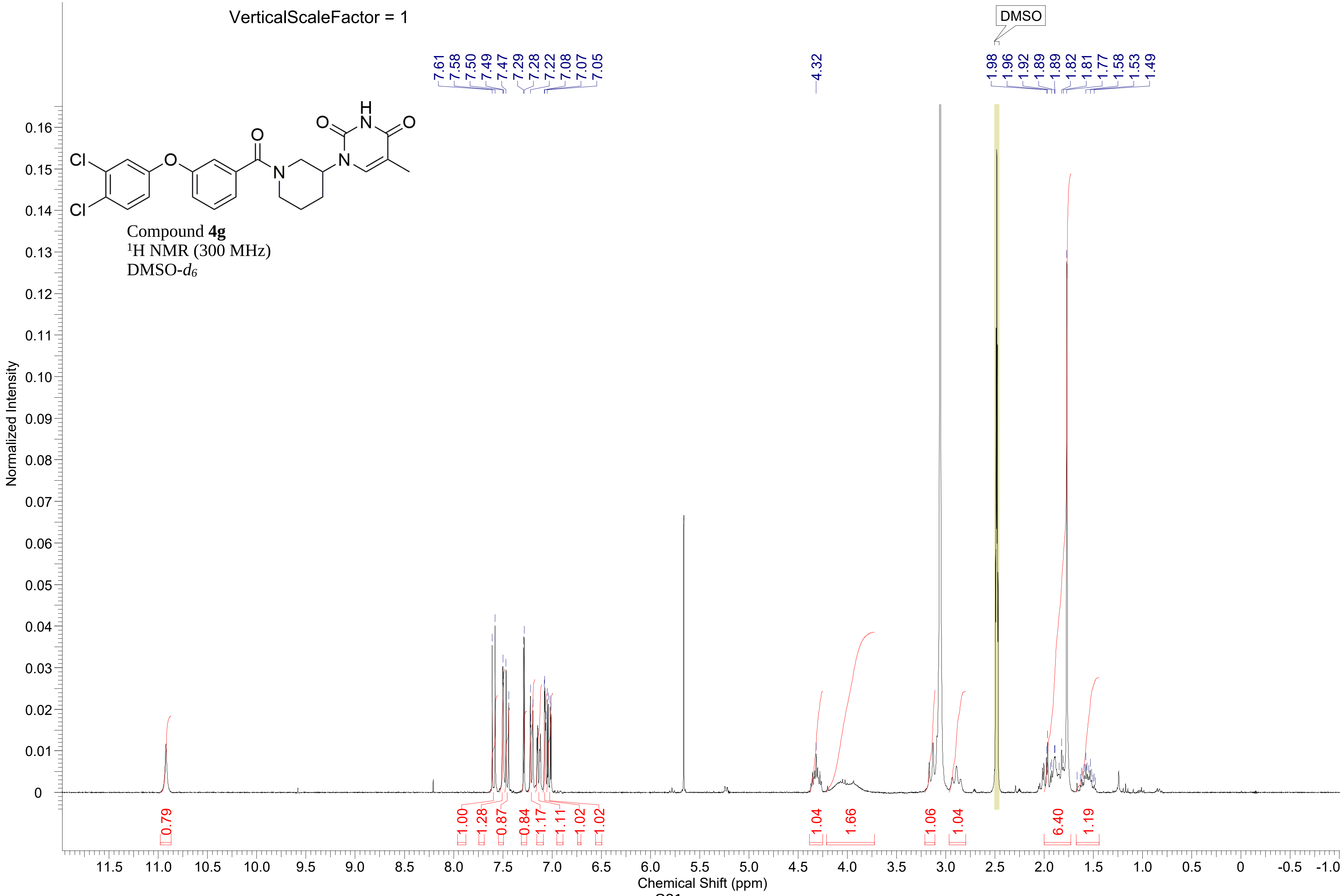
VerticalScaleFactor = 1
DMSO
7.61 7.58 7.50 7.49 7.47 7.29 7.28 7.22 7.08 7.07 7.05
4.32
1.98 1.96 1.92 1.89 1.89 1.82 1.81 1.77 1.58 1.53 1.49
Cl
Cl
O
O
N
N
H
N
O
O
Compound 4g
1H NMR (300 MHz)
DMSO-d6
Normalized Intensity
0.79
1.00 1.28 0.87 0.84 1.17 1.11 1.02 1.02
1.04 1.66
1.06 1.04
6.40 1.19
Chemical Shift (ppm)

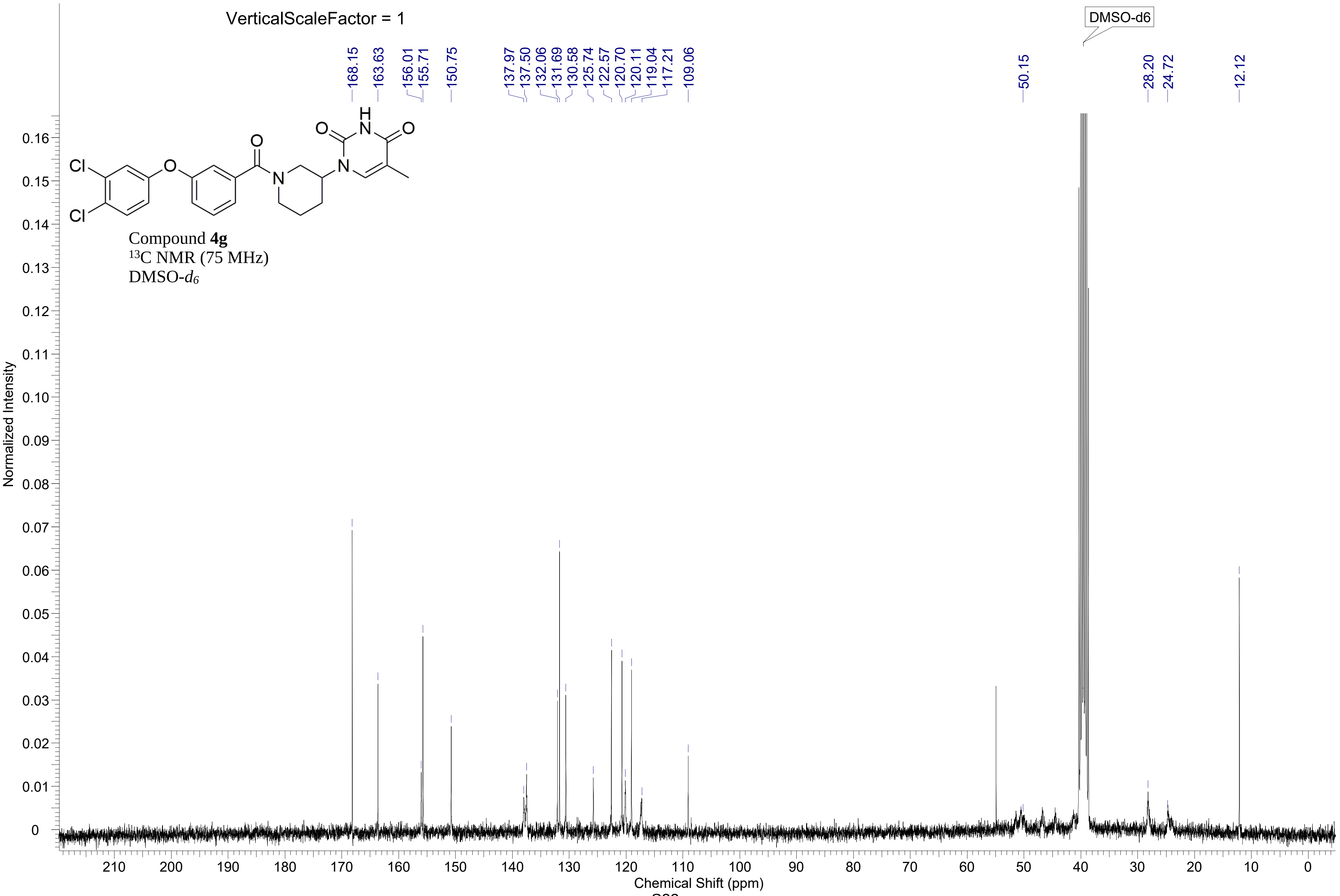
VerticalScaleFactor = 1
DMSO-d6
168.15
163.63
156.01
155.71
150.75
137.97
137.50
132.06
131.69
130.58
125.74
122.57
120.70
120.11
119.04
117.21
109.06
50.15
28.20
24.72
12.12
Cl
Cl
O
O
N
O
H
N
O
N
Compound 4g
13C NMR (75 MHz)
DMSO-d6
Normalized Intensity
Chemical Shift (ppm)

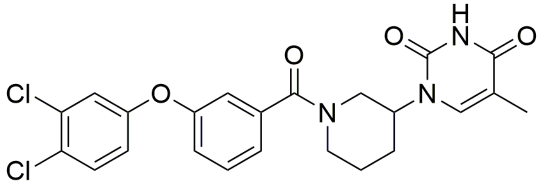

Compound **4g**
HSQC
DMSO-d_6

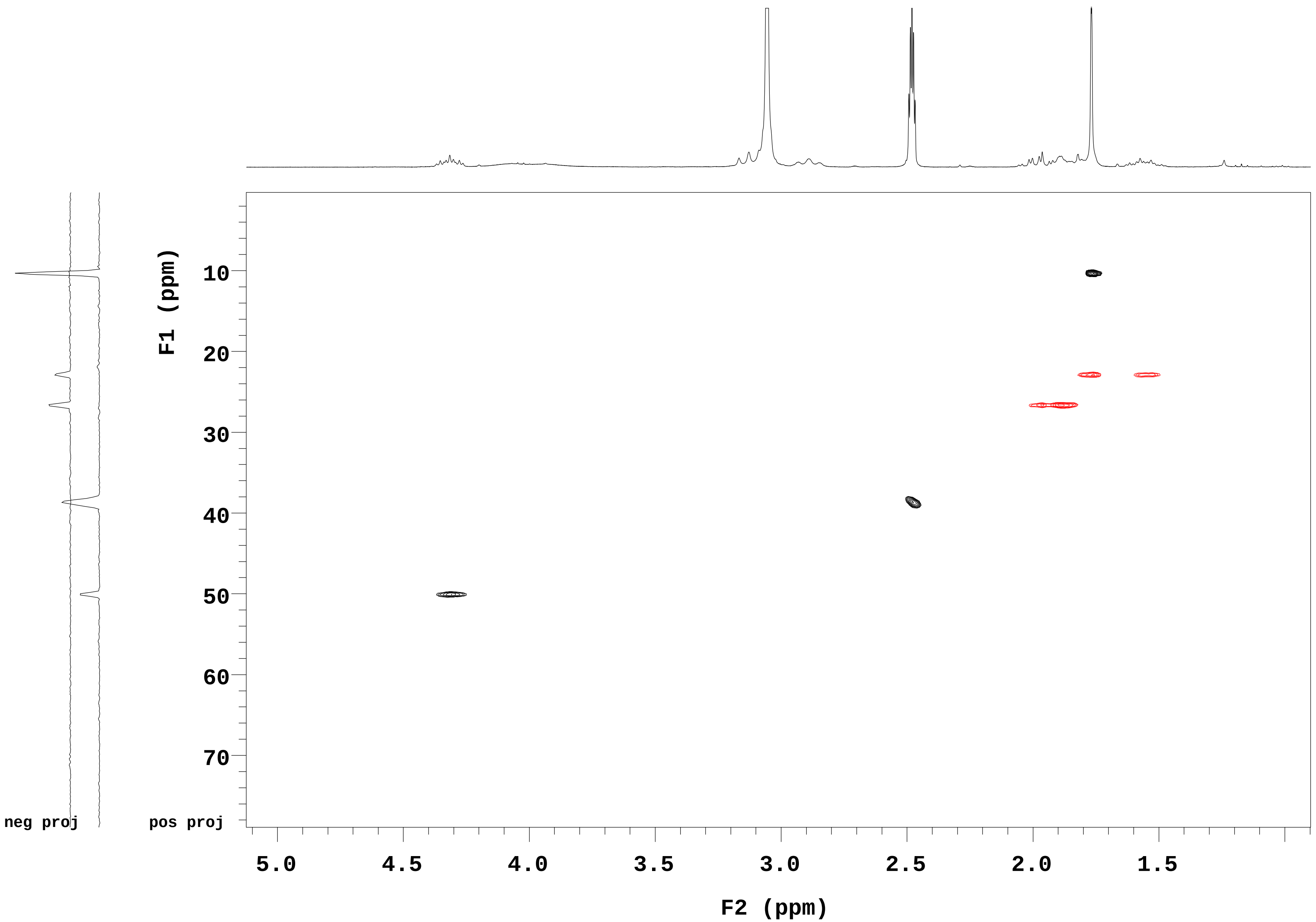

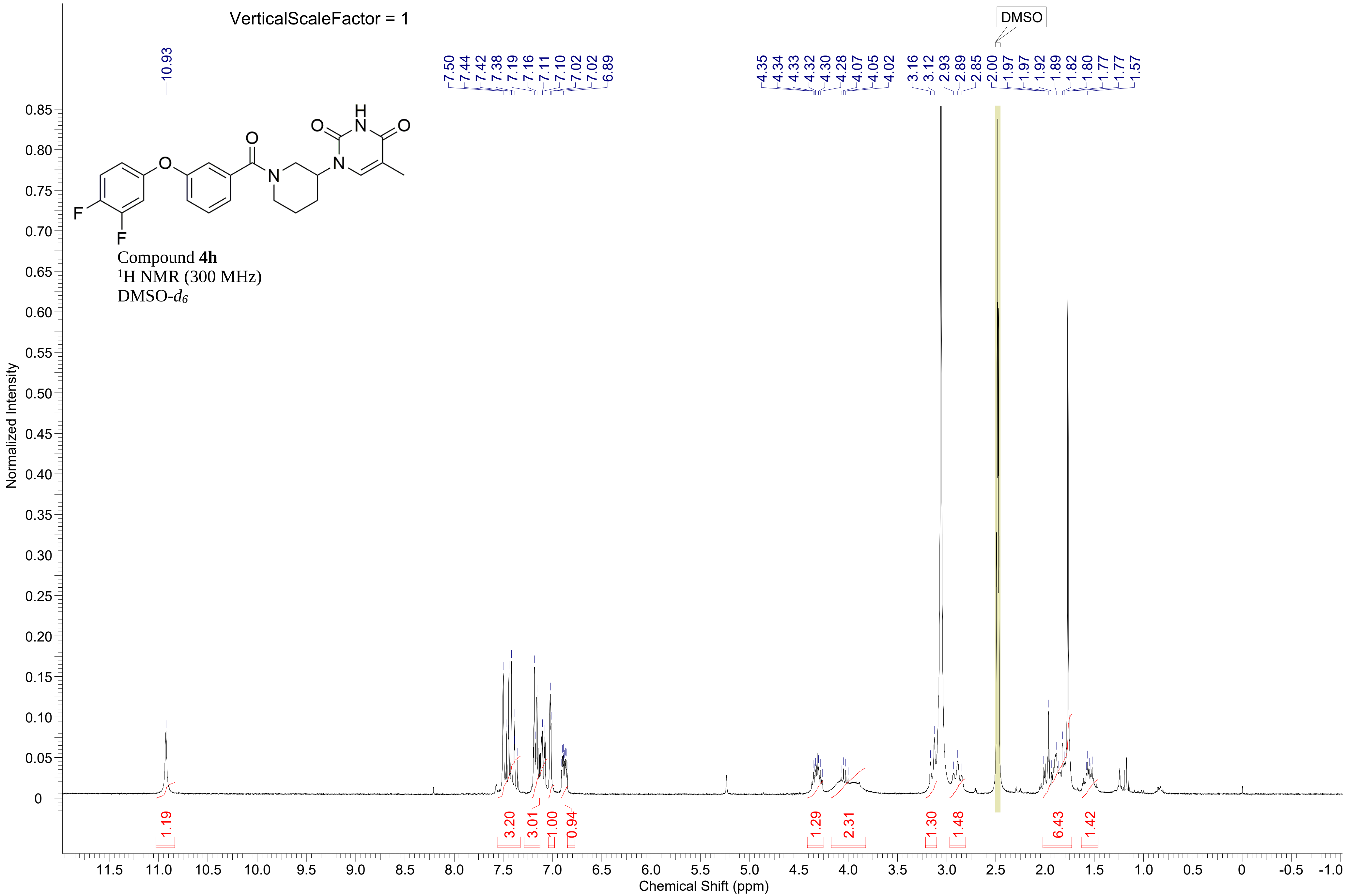
VerticalScaleFactor = 1
DMSO
10.93
7.50 7.44 7.42 7.38 7.19 7.16 7.11 7.10 7.02 7.02 6.89
4.35 4.34 4.33 4.32 4.30 4.28 4.07 4.05 4.02
3.16 3.12 2.93 2.89 2.85 2.00 1.97 1.97 1.92 1.89 1.82 1.80 1.77 1.77 1.57
O
H
N
O
O
N
O
N
F
F
Compound 4h
1H NMR (300 MHz)
DMSO-d6
Normalized Intensity
1.19
3.20
3.01
1.00
0.94
1.29
2.31
1.30
1.48
6.43
1.42
Chemical Shift (ppm)

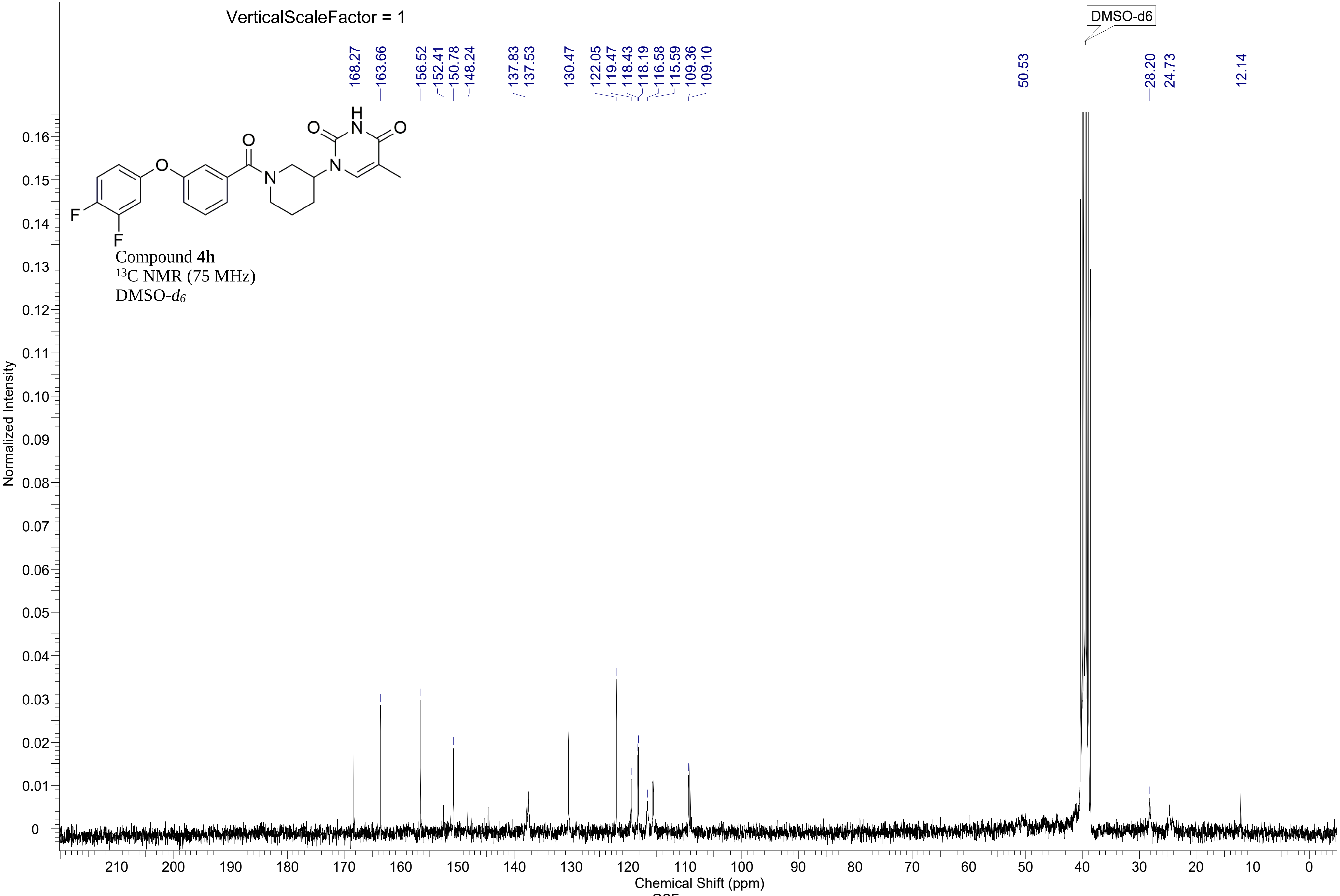
VerticalScaleFactor = 1
DMSO-d6
168.27
163.66
156.52
152.41
150.78
148.24
137.83
137.53
130.47
122.05
119.47
118.43
118.19
116.58
115.59
109.36
109.10
50.53
28.20
24.73
12.14
Compound 4h
13C NMR (75 MHz)
DMSO-d6
Normalized Intensity
Chemical Shift (ppm)

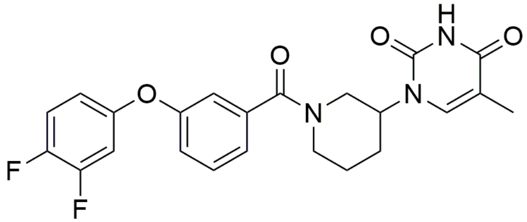

Compound **4h**
HSQC
DMSO-d_6

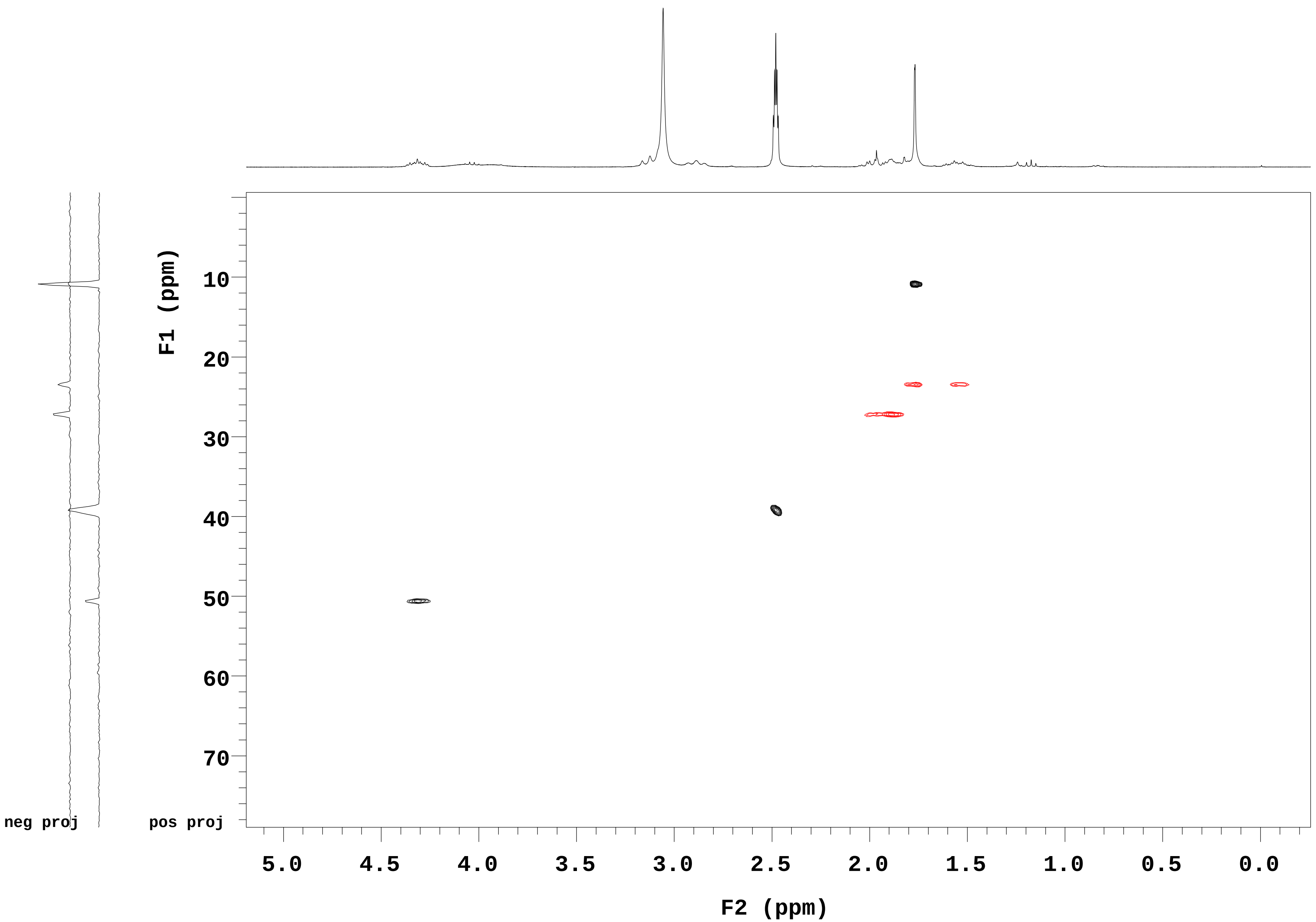

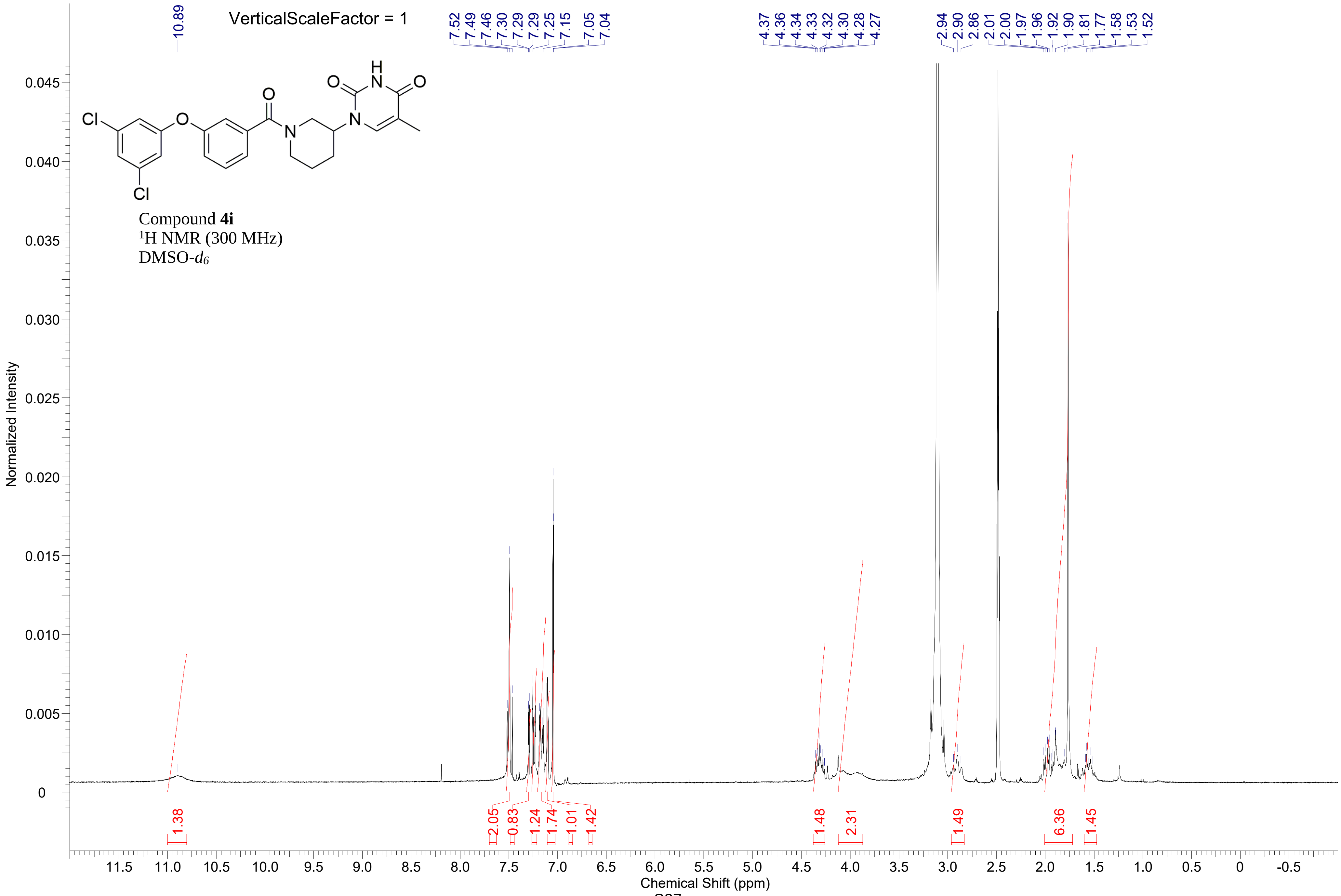
VerticalScaleFactor = 1
Compound 4i
1H NMR (300 MHz)
DMSO-d6
10.89
7.52 7.49 7.46 7.30 7.29 7.29 7.25 7.15 7.05 7.04
4.37 4.36 4.34 4.33 4.32 4.30 4.28 4.27
2.94 2.90 2.86 2.01 2.00 1.97 1.96 1.92 1.90 1.81 1.77 1.58 1.53 1.52
1.38 2.05 0.83 1.24 1.74 1.01 1.42 1.48 2.31 1.49 6.36 1.45
Normalized Intensity
Chemical Shift (ppm)

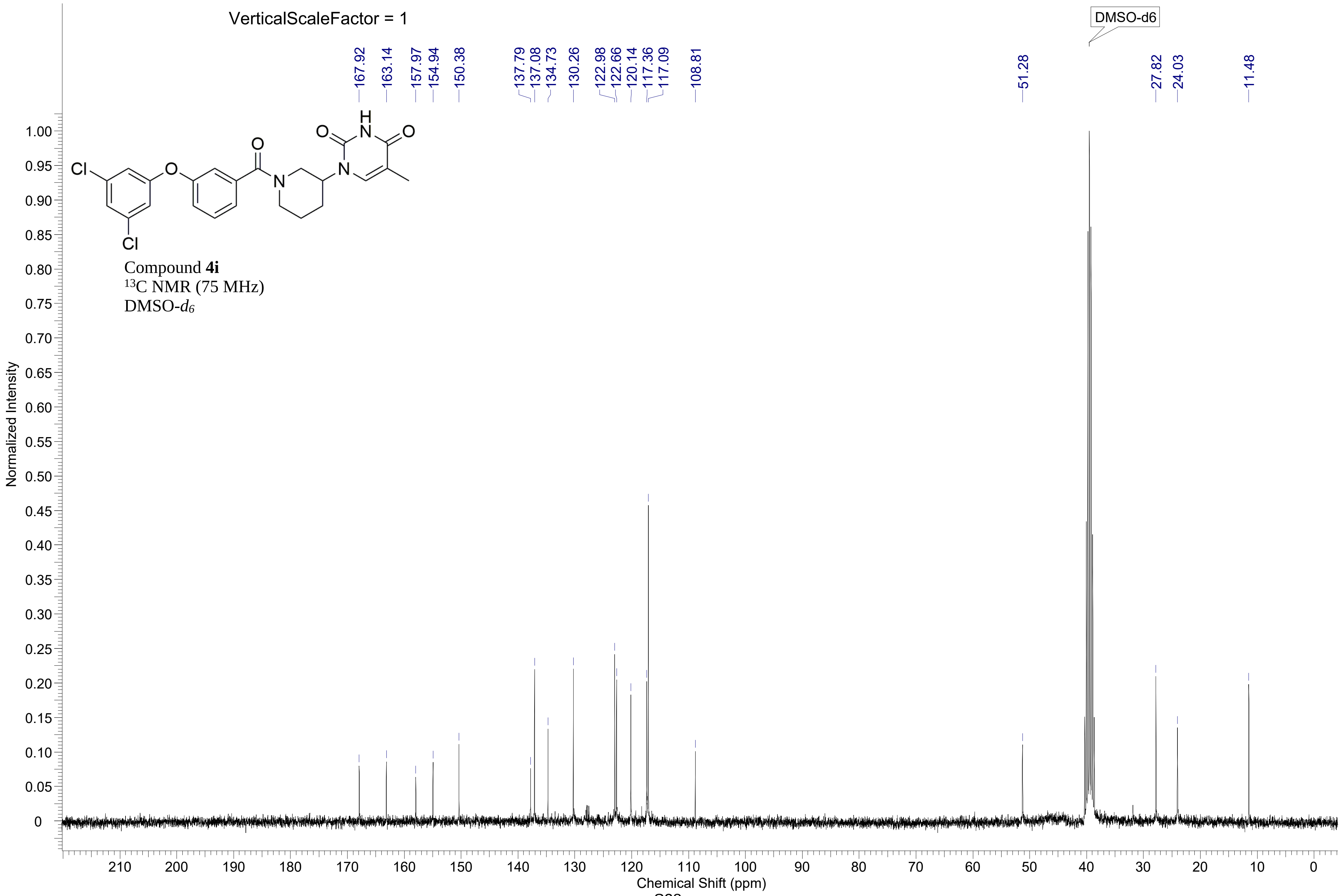
VerticalScaleFactor = 1
DMSO-d6
167.92
163.14
157.97
154.94
150.38
137.79
137.08
134.73
130.26
122.98
122.66
120.14
117.36
117.09
108.81
51.28
27.82
24.03
11.48
Compound **4i**
^{13}C NMR (75 MHz)
DMSO-d_6
Normalized Intensity
Chemical Shift (ppm)

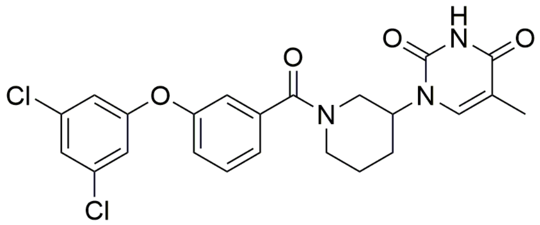

Compound **4i**
HSQC
DMSO-d_6

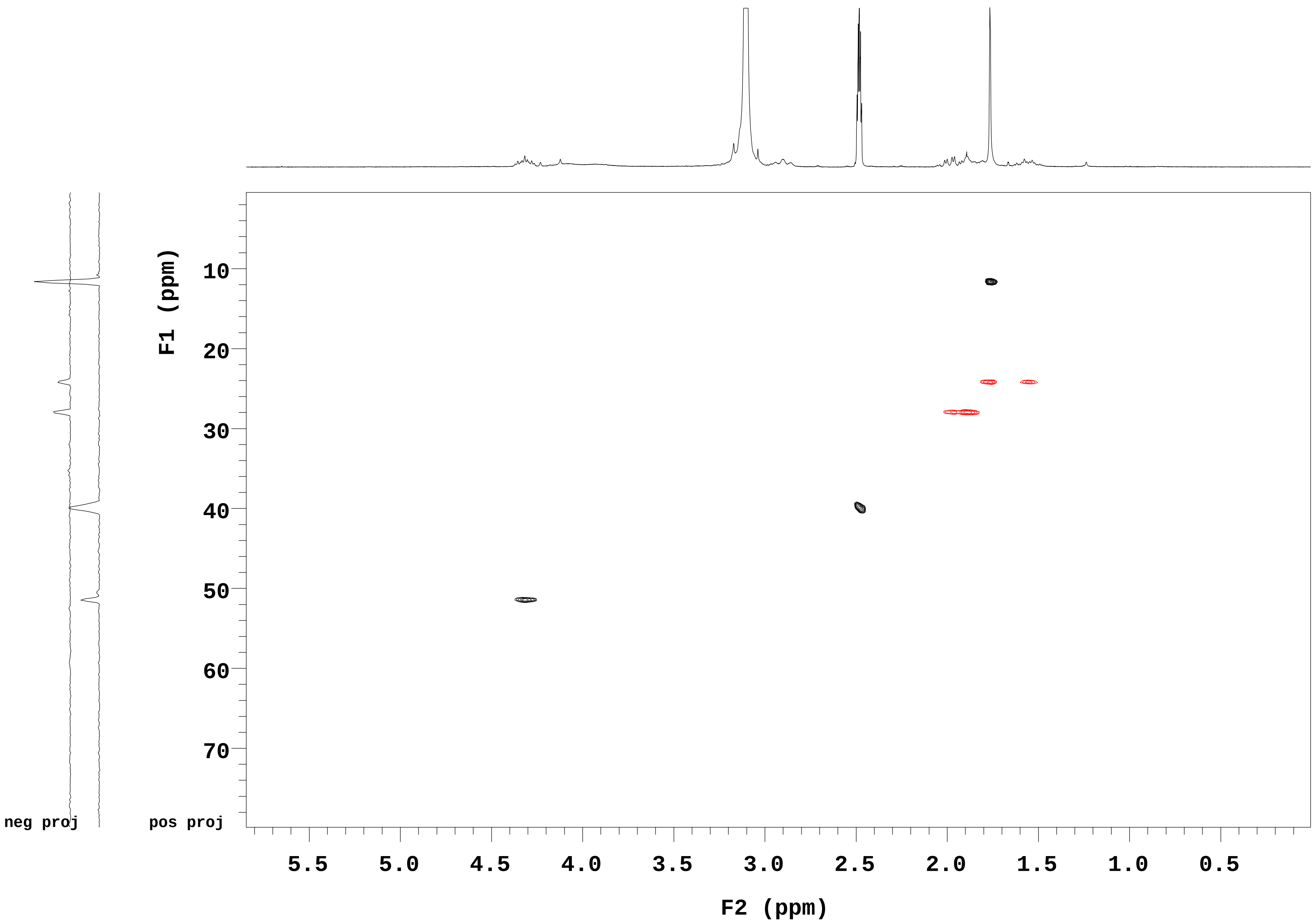

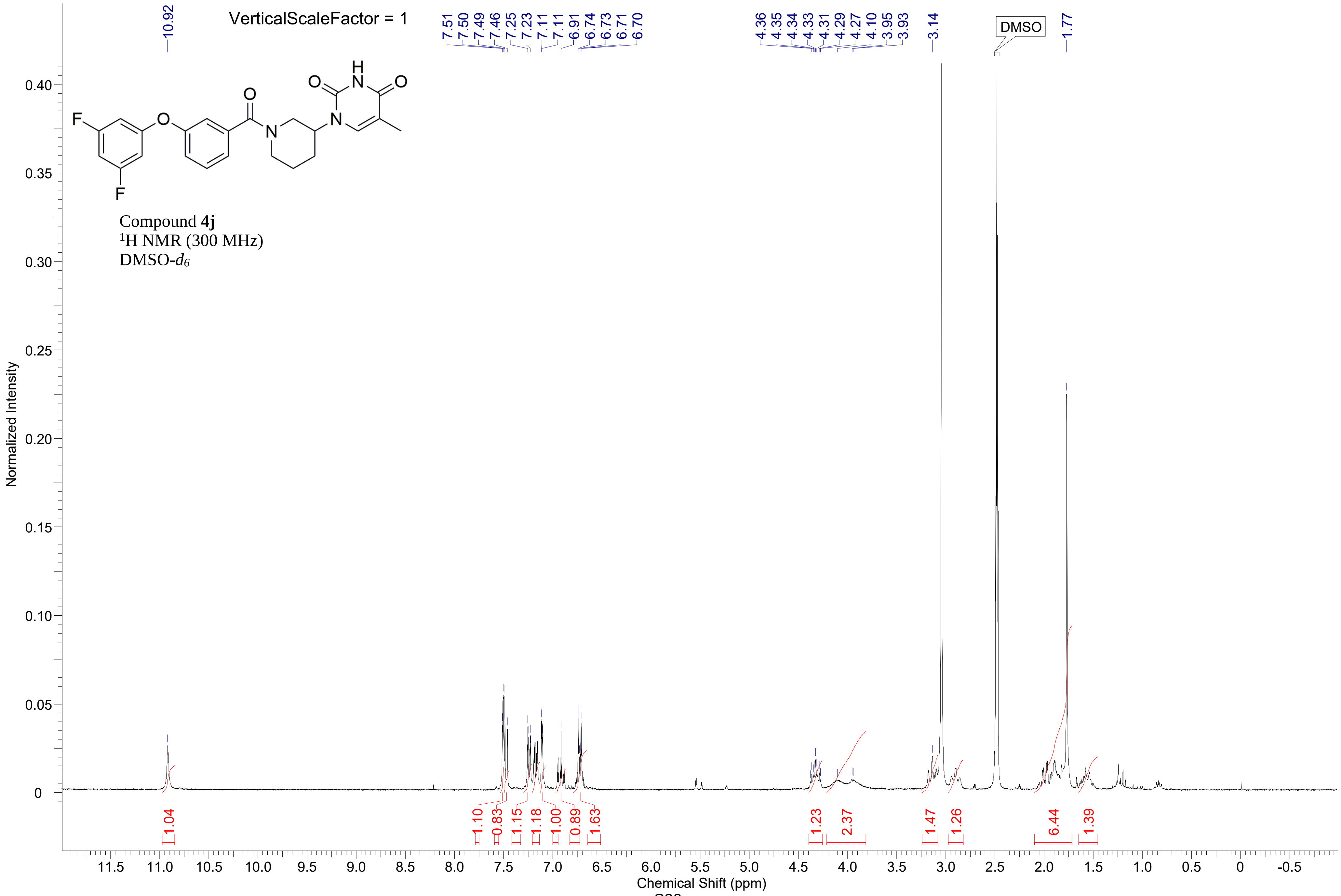
Compound 4j
1H NMR (300 MHz)
DMSO-d6
VerticalScaleFactor = 1
10.92
7.51 7.50 7.49 7.46 7.25 7.23 7.11 7.11 6.91 6.74 6.73 6.71 6.70
4.36 4.35 4.34 4.33 4.31 4.29 4.27 4.10 3.95 3.93
3.14
DMSO
1.77
1.04 1.10 0.83 1.15 1.18 1.00 0.89 1.63 1.23 2.37 1.47 1.26 6.44 1.39
Normalized Intensity
Chemical Shift (ppm)

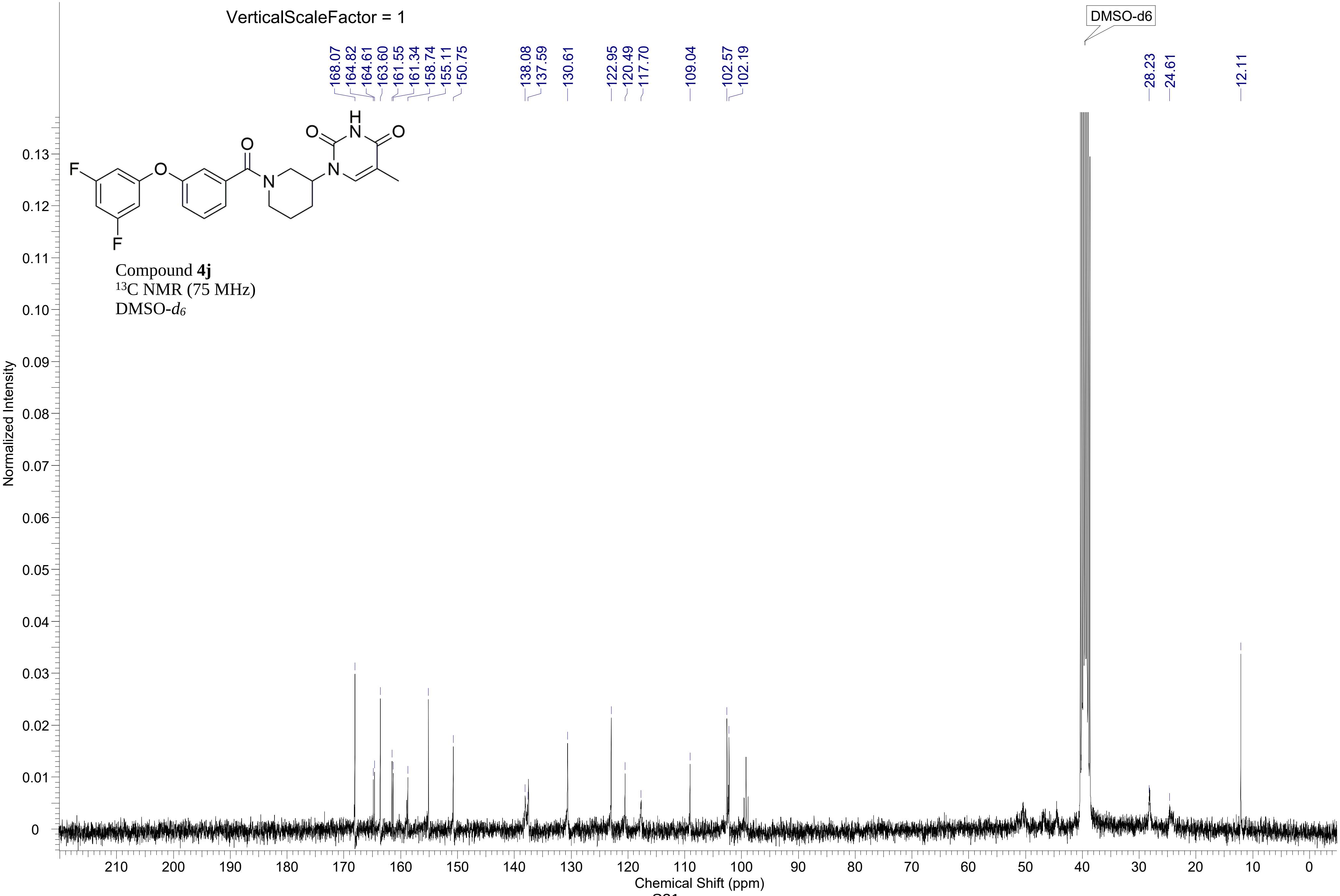

Compound **4j**
^{13}C NMR (75 MHz)
DMSO-d_6

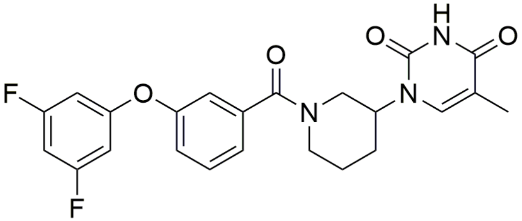

Compound **4j**
HSQC
DMSO-d_6

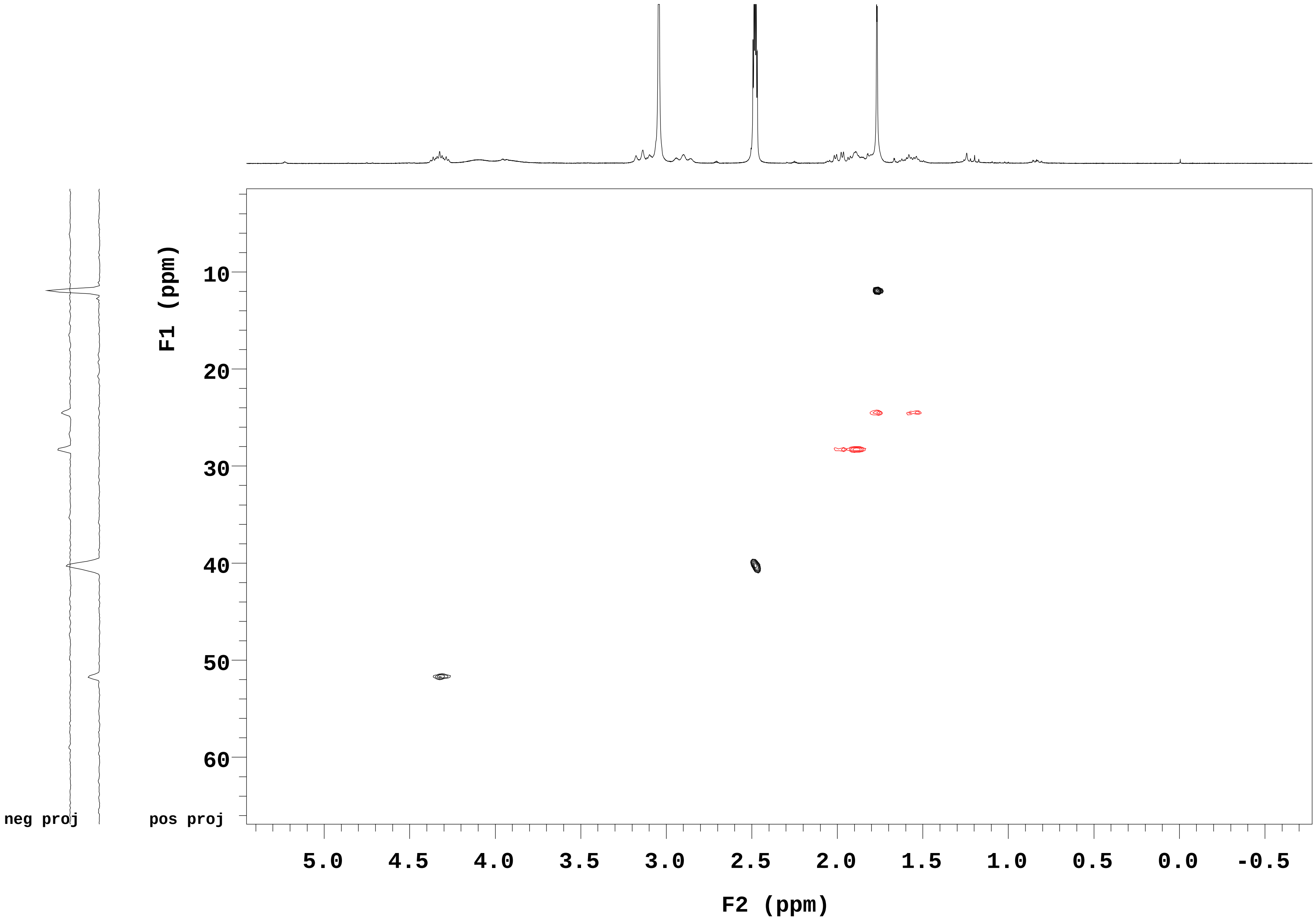

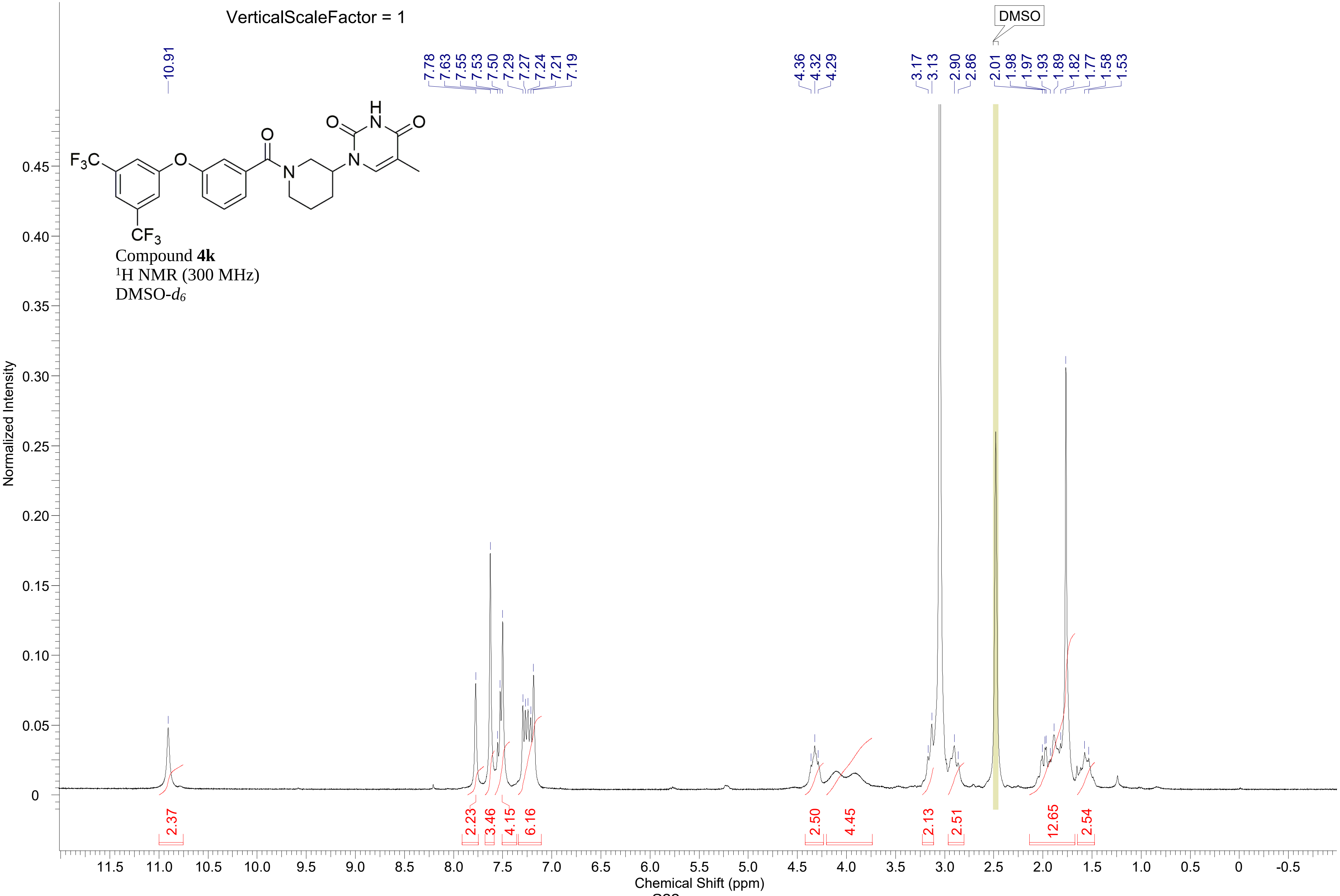
VerticalScaleFactor = 1
DMSO
10.91
7.78 7.63 7.55 7.53 7.50 7.29 7.27 7.24 7.21 7.19
4.36 4.32 4.29
3.17 3.13 2.90 2.86
2.01 1.98 1.97 1.93 1.89 1.82 1.77 1.58 1.53
Compound **4k**
^{1}H NMR (300 MHz)
DMSO-d_6
Normalized Intensity
2.37 2.23 3.46 4.15 6.16 2.50 4.45 2.13 2.51 12.65 2.54
Chemical Shift (ppm)

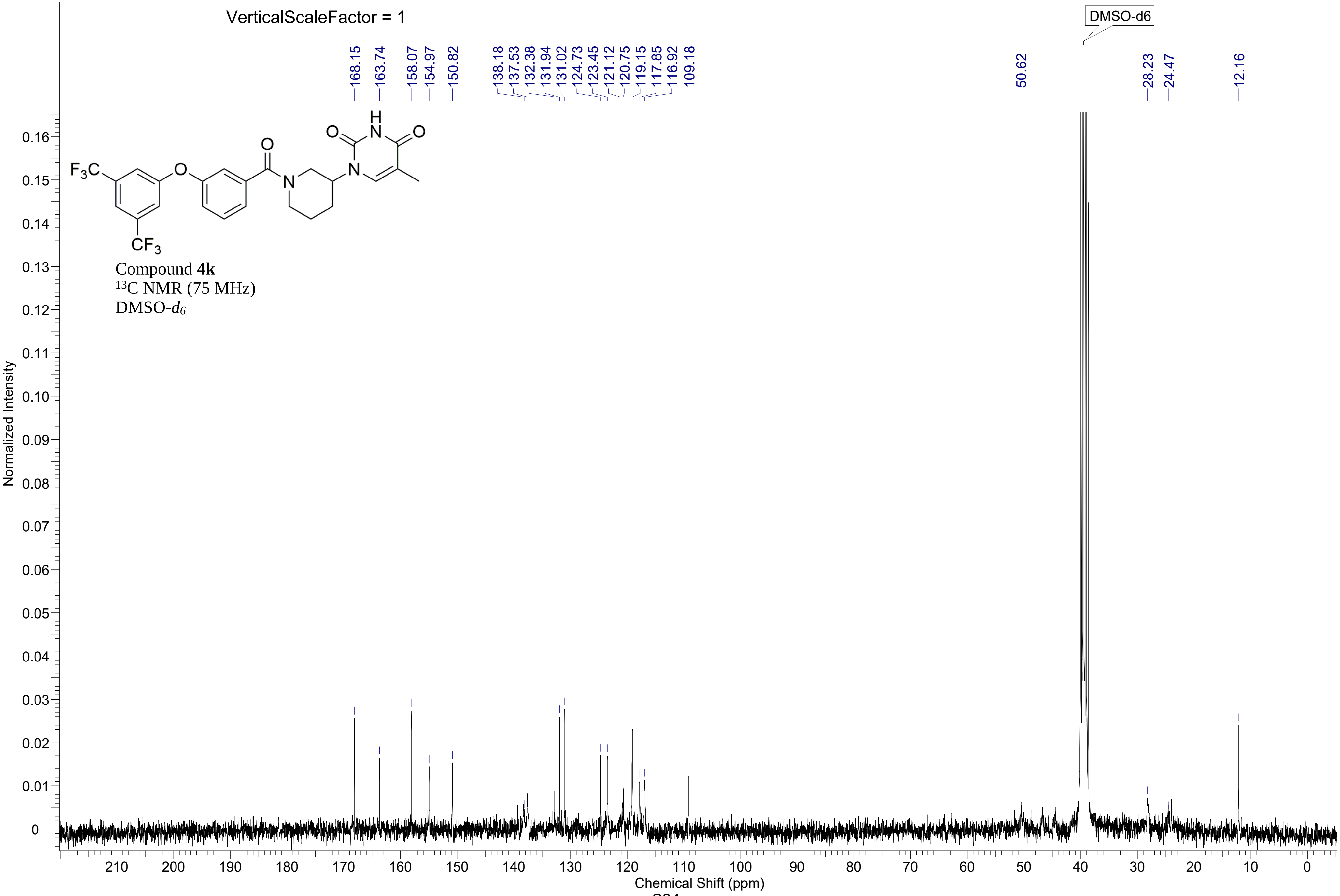
VerticalScaleFactor = 1
DMSO-d6
168.15
163.74
158.07
154.97
150.82
138.18
137.53
132.38
131.94
131.02
124.73
123.45
121.12
120.75
119.15
117.85
116.92
109.18
50.62
28.23
24.47
12.16
Compound **4k**
^{13}C NMR (75 MHz)
DMSO-d_6
Normalized Intensity
Chemical Shift (ppm)

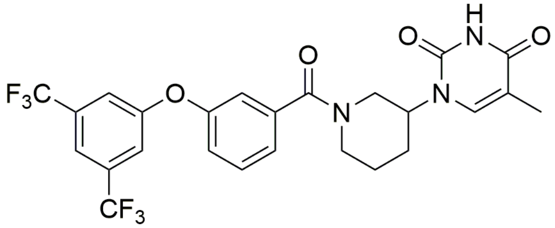

Compound **4h**
HSQC
DMSO-d_6

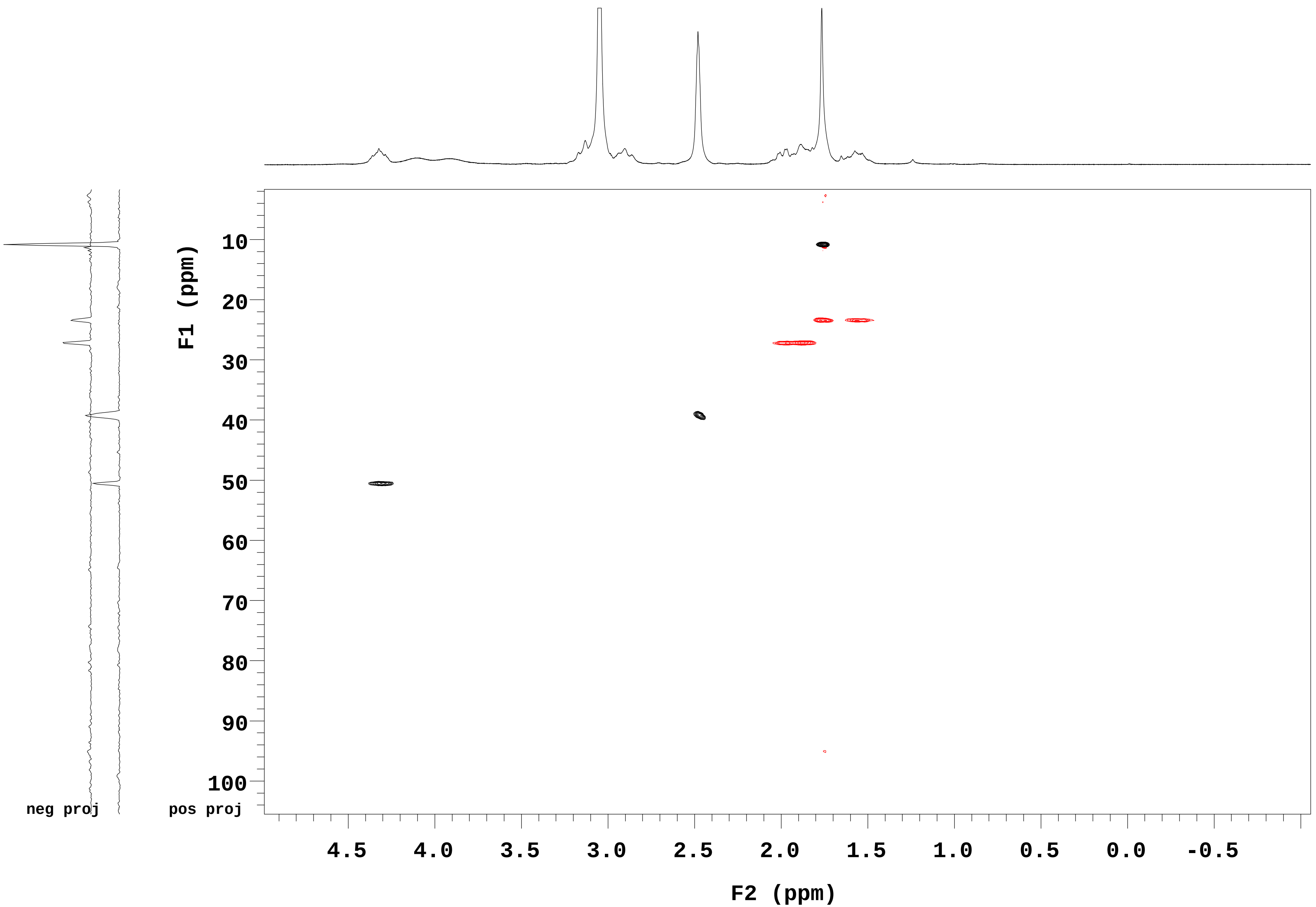

Supplement: Supplemental Material [file IENZ_A_1662790_SM6762.pdf]
